# Gut microbiota-derived TNF-α triggers dopaminergic neuron ferroptosis via TNFR1-NF-κB-ATF4 axis in Parkinson’s disease

**DOI:** 10.1038/s41420-026-03281-x

**Published:** 2026-08-03

**Authors:** Zixia Zhang, Sufang Zhong, Liangliang Gao, Baodong Chen, Peng Huang

**Affiliations:** 1 Department of Hematology, South China Hospital of Shenzhen University, Shenzhen, Guangdong PR China; 2 Department of Neurosurgery, Peking University Shenzhen Hospital, Shenzhen Peking University-The Hong Kong University of Science and Technology Medical Center, Shenzhen, Guangdong PR China; 3 Department of Neurosurgery, Peking University Shenzhen Hospital, Shenzhen, Guangdong PR China

**Keywords:** Neuroscience, Microbiology

## Abstract

Parkinson’s disease (PD) pathogenesis remains incompletely understood, with the causal link between gut microbiota dysbiosis and central neurodegeneration unclarified. This study aimed to unravel this complex interplay by integrating Mendelian randomization (MR) analysis, animal models, cellular experiments, and transcriptomic profiling. MR analysis identified *Clostridium sensu stricto 1* and *Candidatus Soleaferrea* as novel genetic risk factors for PD, providing causal evidence for gut microbiota-driven PD pathogenesis. Using a chronic MPTP-induced PD mouse model, we confirmed that gut microbiota dysbiosis is accompanied by elevated tumor necrosis factor-α (TNF-α) in the intestine, plasma, and midbrain, along with dopaminergic neuron loss and motor deficits. Fecal microbiota transplantation (FMT) from healthy mice reversed these phenotypes, restoring gut homeostasis, reducing TNF-α levels, and preserving dopaminergic neurons. Transcriptomic analysis of mouse midbrain and human PD substantia nigra (GEO datasets) revealed ferroptosis as a key enriched pathway, with activating transcription factor 4 (ATF4) significantly upregulated in both PD models and patients. Mechanistically, TNF-α induced ATF4 overexpression in a concentration- and time-dependent manner, triggering dopaminergic neuron ferroptosis. This effect was mediated by TNF-α binding to TNFR1 (not TNFR2), activating the NF-κB pathway, and subsequently upregulating ATF4. Knockdown of ATF4 or inhibition of NF-κB reversed TNF-α-induced ferroptosis, as evidenced by restored glutathione (GSH) levels, reduced malondialdehyde (MDA) accumulation, and improved cell viability. Our findings establish a novel gut-brain axis-mediated pathogenic pathway: gut microbiota dysbiosis drives TNF-α-TNFR1-NF-κB-ATF4 signaling, leading to dopaminergic neuron ferroptosis and PD progression. This study bridges the gap between peripheral microbial dysbiosis and central neurodegeneration, identifies potential therapeutic targets, and highlights FMT as a promising intervention for PD.

## Introduction

Parkinson’s disease (PD), a devastating neurodegenerative condition with a rising global prevalence, poses an unprecedented challenge to public health systems worldwide [1]. Beyond the well-characterized motor manifestations (e.g., bradykinesia, tremor, rigidity) driven by the selective degeneration of dopaminergic (DA) neurons in the substantia nigra pars compacta (SNpc) and pathological aggregation of α-synuclein (α-syn), PD patients often suffer from disabling non-motor symptoms—particularly gastrointestinal disturbances like constipation—that may manifest up to decades prior to motor onset [2, 3]. Despite remarkable advances in understanding PD pathology, the precise initiating events of neurodegeneration remain elusive, and current therapies only provide symptomatic relief without modifying the underlying disease course [4]. This therapeutic impasse highlights an urgent need to re-examine PD pathogenesis from alternative perspectives and identify novel, targetable pathways.

The traditional central nervous system (CNS)-centric paradigm of PD has been increasingly challenged by the emerging gut-brain axis hypothesis, which posits that peripheral perturbations—especially gut microbiota dysbiosis—may serve as upstream triggers of central neurodegeneration [5]. Clinical and preclinical studies have consistently documented alterations in gut microbial communities in PD, including shifts in the relative abundance of *Faecalibacterium, Roseburia, Akkermansiaceae*, and other key taxa [6]. However, these observations are largely correlational, and critical questions remain unresolved: Does gut microbiota dysbiosis actively drive PD pathogenesis, or is it a secondary consequence of neuronal dysfunction or systemic changes? Furthermore, the molecular messengers and signaling cascades that bridge peripheral microbial perturbations to central DA neuron damage have not been fully delineated, limiting the translation of gut microbiota research into actionable therapies [7].

Inflammation has emerged as a central mediator of gut-brain crosstalk in neurodegenerative diseases, yet its specific role in PD-associated gut-brain communication remains poorly defined [8]. Gut microbiota dysbiosis can disrupt intestinal mucosal barrier integrity, leading to the systemic release of pro-inflammatory cytokines that may infiltrate the CNS and amplify neuroinflammation [9]. Tumor necrosis factor-α (TNF-α), a key pro-inflammatory mediator, exhibits aberrant elevation in the serum and cerebrospinal fluid of patients with PD, making the TNF-α/soluble tumor necrosis factor receptors (TNF-α/sTNFRs) a promising peripheral candidate biomarker for the clinical diagnosis of PD [10]. Preclinical investigations have demonstrated that TNF-α inhibition confers a protective effect against DA neuronal loss in PD models [11]. Nevertheless, the specific modality of DA neuronal death selectively induced by TNF-α and the underlying regulatory mechanisms of the downstream signaling cascades remain largely elusive. Furthermore, the precise pathways governing TNF-α production upon gut microbiota dysbiosis and its translocation across the blood-brain barrier (BBB) to trigger midbrain DA neuronal demise have been poorly characterized in current research.

Ferroptosis, an iron-dependent, lipid peroxidation-driven form of regulated cell death, has recently gained attention as a potential mechanism of DA neuron loss in PD [12]. Pathological iron accumulation in the SNpc is a hallmark of PD, and genetic or pharmacological targeting of ferroptosis pathways protects DA neurons in preclinical models [13]. Activating transcription factor 4 (ATF4), a master regulator of cellular stress responses, has been reported to exert context-dependent effects on ferroptosis in various cell types [14, 15], but its role in PD-specific ferroptosis—particularly in DA neurons—has not been explored. A key unresolved question is whether ATF4 acts as a convergence point for inflammatory signals and ferroptosis pathways in DA neurons, and whether this crosstalk is uniquely dysregulated in PD. Notably, the quantitative relationship between ATF4 expression and ferroptosis sensitivity in DA neurons, as well as its relevance to human PD pathology, remains unestablished.

To address these critical knowledge gaps, the present study designed a multi-dimensional, translational approach to dissect the gut-brain axis in PD with three interrelated objectives: (1) Validate the causal role of gut microbiota dysbiosis in PD using Mendelian randomization (MR) analysis—an unbiased genetic tool that circumvents confounding and reverse causation, complemented by cross-validation with human clinical datasets; (2) Decipher the molecular mechanism linking gut microbiota dysbiosis to DA neuron ferroptosis, with a focus on the TNF-α-mediated signaling cascade and the regulatory role of ATF4, while exploring the specificity of this pathway to DA neurons; (3) Evaluate the therapeutic potential of fecal microbiota transplantation (FMT)—a direct and non-invasive strategy to restore gut microbiota homeostasis—in alleviating PD-related pathology, including α-syn aggregation and DA neuron loss, as well as motor and non-motor deficits.

To achieve these goals, we integrated MR analysis of human genetic data, chronic MPTP-induced PD mouse models with FMT intervention, transcriptomic profiling of mouse midbrain tissues and human PD SNpc samples, and cellular experiments using MPP⁺-induced DA neuron models and ferroptosis induction systems. Our findings identify a novel, targetable pathogenic pathway: gut microbiota dysbiosis drives selective TNF-α release, which translocates across the BBB and activates the TNFR1-NF-κB-ATF4 signaling axis in midbrain DA neurons, ultimately priming these cells for ferroptosis and accelerating PD progression. This study not only provides definitive causal evidence for the role of gut microbiota in PD pathogenesis but also uncovers a unique molecular crosstalk between inflammation and ferroptosis in DA neurons. Furthermore, our preclinical data support FMT as a promising therapeutic strategy for PD by disrupting this pathogenic cascade, offering a non-pharmacological alternative to modify disease progression.

## Results

### Exploring the causal effect of gut microbiota in the pathogenesis of PD

**Fig. 1:**
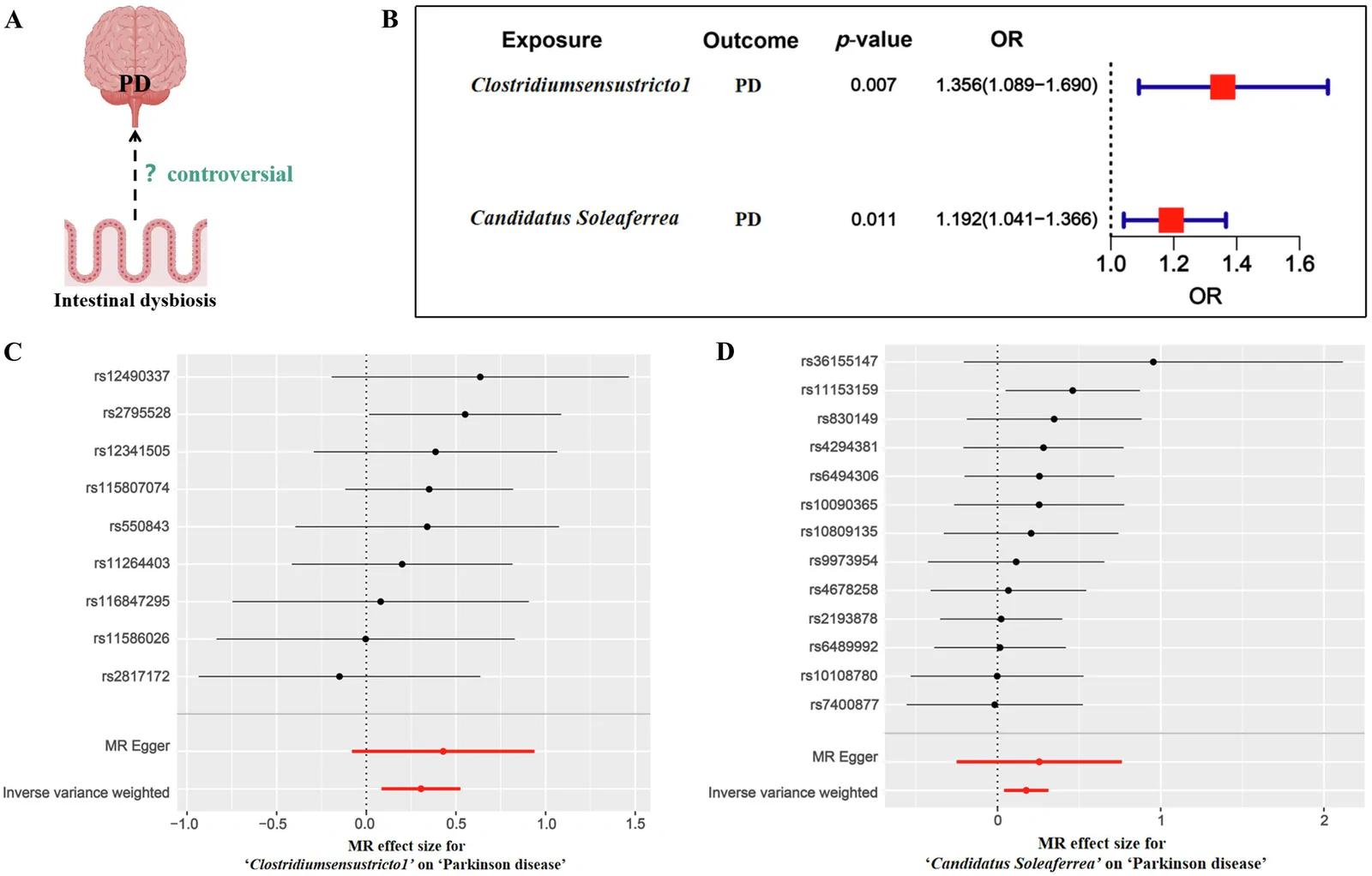
Exploring the causal effect of gut microbiota on PD. **A** Conceptual schematic diagram of the long-standing controversy regarding whether gut microbiota dysbiosis can induce PD. **B** Forest plot of causal relationships between *Clostridium sensu stricto 1* and *Candidatus Soleaferrea* in PD. **C** MR effect-related pathogenic risk analysis of *Clostridium sensu stricto 1* SNP loci in PD. **D** MR effect-related pathogenic risk analysis of *Candidatus Soleaferrea* SNP loci in PD.

The causal link between gut microbiota dysbiosis and PD remains elusive and controversial (Fig. 1A). Our Mendelian randomization (MR) analysis identifies gut microbiota dysbiosis as a risk factor for PD. Initially, we identified single nucleotide polymorphisms (SNPs) that were associated with the gut microbiome at the genus level. The screening criteria for these SNPs were a significance level of *P* < 1×10^5^ and should satisfy the conditions of *r*^2^ < 0.001 and a distance >10,000 kb, with an F-statistic parameter > 10. The analysis results revealed that there are two types of gut microbiota associated with PD, and the detailed information on the 22 SNPs concerning the two types of gut microbiota. As depicted in Fig. 1B, the MR analysis shows that the genetic prediction of two types of gut microbiota (*Clostridium sensu stricto 1* and *Candidatus Soleaferrea*) is associated with an increased risk of PD. The *Clostridium sensu stricto 1* (OR = 1.356, 95%CI = 1.089 ~ 1.69, *P* = 0.007), and the *Candidatus Soleaferrea* (OR = 1.192, 95%CI = 1.041 ~ 1.366, *P* = 0.011) significantly elevate the risk of developing PD (Fig. 1B). The *Clostridium sensu stricto 1* contains 9 SNPs sites and *Candidatus Soleaferrea* contains 13 SNPs sites. Through MR Egger and inverse variance weighted (IVW) analysis, it was found that the overall effect of the *Clostridium sensu stricto 1* and *Candidatus Soleaferrea* is a high-risk factor for PD pathogenesis (Fig. 1C, D). On this basis, we demonstrated that dysregulated gut microbiota raises the risk of developing PD.

### Sensitivity analysis of gut microbiota

**Fig. 2:**
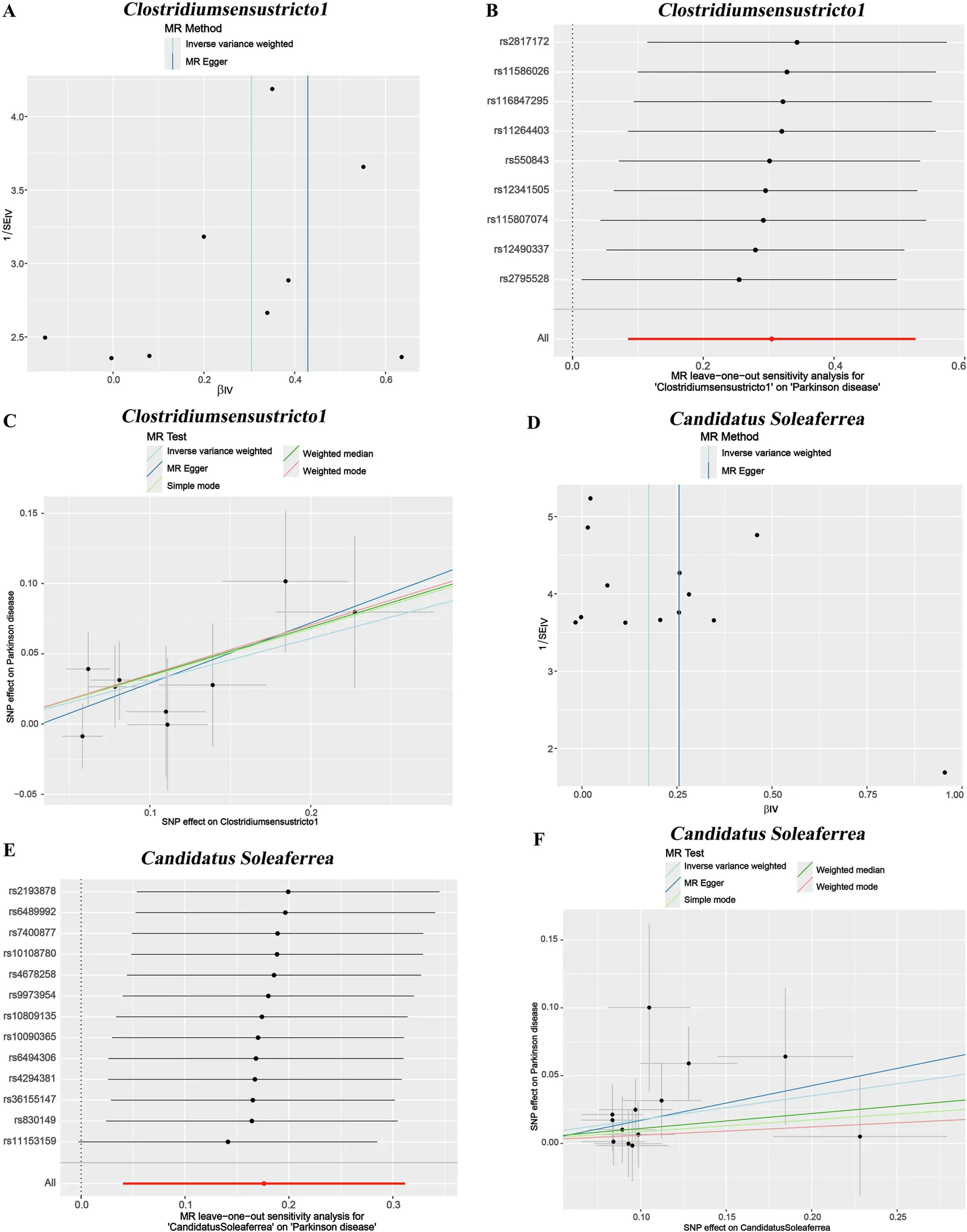
Sensitivity analysis for *Clostridium sensu stricto1* and *Candidatus Soleaferrea* on PD. **A** Funnel plots illustrating the causal association of *Clostridium sensu stricto 1* with PD pathogenicity. **B** Mendelian randomization leave-one-out sensitivity analyses for the causal effect of *Clostridium sensu stricto 1* on PD, evaluating the impact of individual SNPs on overall causal estimates. **C** Scatter plots demonstrating the consistency of regression lines for the causal association between *Clostridium sensu stricto 1* and PD pathogenic risk across distinct analytical methods. **D** Funnel plots illustrating the causal association of *Candidatus Soleaferrea* with PD pathogenicity. **E** Mendelian randomization leave-one-out sensitivity analyses for the causal effect of *Candidatus Soleaferrea* on PD. **F** Scatter plots demonstrating the consistency of regression lines for the causal association between *Candidatus Soleaferrea* and PD pathogenic risk across distinct analytical methods.

We further evaluated through MR-Egger-intercept and found that there is no significant horizontal pleiotropy in the SNPs contained in the microbiome. In Cochran’s Q test, we found no substantial heterogeneity, suggesting that the SNPs contained in the microbiome are not associated with outcomes through confounding factors. Moreover, the MR-PRESSO global test did not find any evidence of horizontal pleiotropy (*P* > 0.05), as detailed in Table 1. A leave-one-out sensitivity analysis was performed for *Clostridium sensu stricto 1*, which enabled the substantial elimination of biases arising from horizontal pleiotropy. The funnel plot results revealed a predominantly symmetrical distribution of the causal effects of microbiome-derived SNPs on PD pathogenicity, indicative of negligible pleiotropic bias (Fig. 2A). Furthermore, no individual SNP exerted a significant influence on the overall causal estimate, which further strengthened the reliability of the MR analysis results (Fig. 2B). The scatter plot demonstrated that the regression lines for the association between microbiome SNPs and PD pathogenic risk, as derived from distinct analytical approaches, were essentially consistent (Fig. 2C). Notably, consistent findings were obtained in the parallel sensitivity analysis for *Candidatus Soleaferrea* (Fig. 2D–F).

**Table 1:** Sensitivity analysis for gut microbiota on PD by MR analysis.

Exposure	Pleiotropy		Heterogeneity	MR-PRESSO global test
	MR-egger intercept	MR-Egger intercept *P* value	Cochran’s *Q* *P* value	*P* value
*Clostridium sensu stricto 1*	−0.0138	0.612	0.840	0.902
*Candidatus Soleaferrea*	−0.00853	0.755	0.824	0.898

### Gut microbiota dysbiosis and increased release of TNF-α in PD mice

**Fig. 3:**
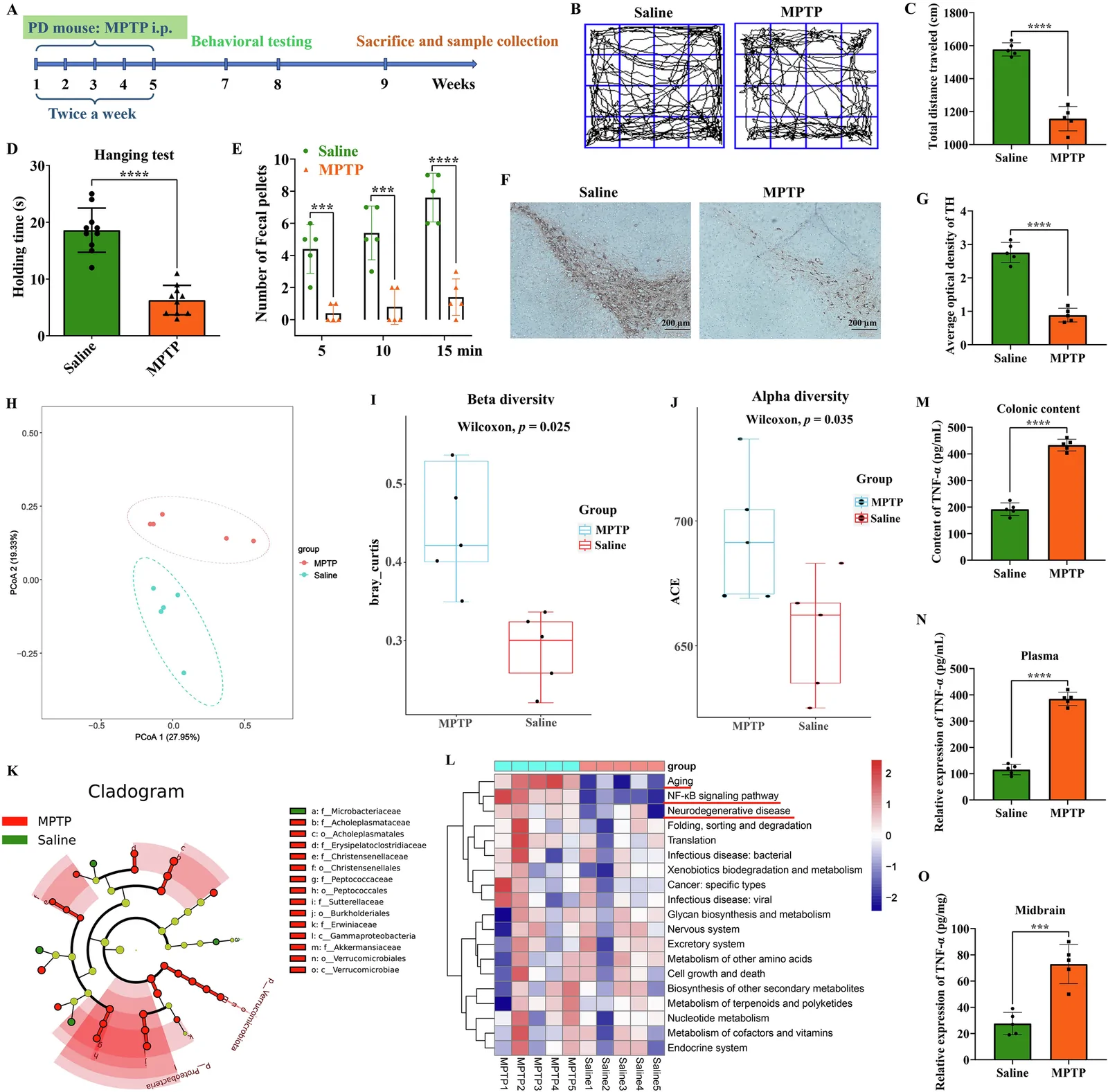
The gut microbiota of PD mice is dysregulated, with increased release of gut-derived inflammatory factors. **A** Schematic of the construction of the chronic PD mice model. *n* = 10/group. **B**, **C** Open field test and statistical graph. *n* = 5/group. **D** Hanging test and statistical analysis. *n* = 10/group. **E** Mouse feces collection and statistical analysis. *n* = 5/group. **F**, **G** IHC to detect the expression of TH in the midbrain and statistical graph; Scale bar: 200 µm. *n* = 5/group. **H** PCoA plots of the weighted UniFrac distances using the relative abundance of OTUs. Each point represents a sample from the control or MPTP. *n* = 5/group. **I** Beta-diversity comparison of the gut microbiota. **J** α-diversity comparison of the gut microbiota. **K** The cladogram displays the significantly different bacterial groups between the two groups, with red and green indicating the bacterial groups that are significantly higher in the PD and control, respectively. **L** Analysis of the functions of microbial communities. ELISA was used to detect the expression level of TNF-α in intestinal contents (**M**), plasma (**N**), and midbrain tissue (**O**), respectively. *n* = 5/group. Data are presented as mean ± SEM. NS indicates no statistical significance. The experiment used unpaired two-tailed Student’s *t* test as the statistical method. ***P < 0.001 and ****P < 0.0001.

We established a PD mouse model using the neurotoxicant 1-methyl-4-phenyl- 1,2,3,6-tetrahydropyridine (MPTP) (30 mg/kg i.p. over five weeks) and detected changes in the gut microbiota (Fig. 3A). After 7 days, behavioral tests were used to evaluate whether the PD mice had been successfully constructed. The open field test (OFT) results showed that the total distance traveled by the MPTP-induced PD mouse model (PD mice) was significantly lower than that of the normal saline-treated control mice (control mice) (Fig. 3B, C). The mouse hanging test results indicated that the hanging time of the control mice was significantly longer than that of the PD mice (Fig. 3D). In the same period of time, the number of fecal pellets excreted by PD mice is significantly less than that of control mice (Fig. 3E). Immunohistochemistry (IHC) staining demonstrated that PD mice had significantly lower TH-positive cells in the midbrain compared with control mice (Fig. 3F, G). The results indicated that we successfully established the PD mouse model.

We further detected changes in the gut microbiota of mice to verify whether the microbiota is dysregulated in PD mice. Therefore, we conducted 16S rRNA V3-V4 region sequencing analysis on the colonic contents of PD mice to examine the disrupted microbial communities. Through principal co-ordinates analysis (PCoA), the similarity of gut microbial between the PD mice and control mice was examined. As shown in Fig. 3H, the gut microbiota of these groups is distinctly separated, indicating that there is a difference in the overall similarity of bacterial between the PD mice and control mice. According to the Bray-Curtis algorithm, a beta-diversity analysis that estimates differences in microbial between different groups, there is an increase in beta-diversity in PD mice compared to the control mice (Fig. 3I). Interestingly, the number of OTUs estimated by the alternating conditional expectation (ACE) algorithm was used to assess alpha-diversity within samples. It was found that there was also a statistical difference in microbial community diversity between PD mice and control mice (Fig. 3J). These results demonstrate that the gut microbiota composition of PD mice undergoes significant aberrant alterations.

Furthermore, linear discriminant analysis effect size (LEfSe) analysis was conducted to determine the differential abundance of gut microbiota in PD mice. A significant shift in the microbial based on relative abundance is displayed in cladogram. In PD mice, a significant enrichment of the *Acholeplasmataceae, Acholeplasmatales, Erysipelatoclostridiaceae, Christensenellaceae, Christensenellales, Peptococcaceae, Peptococcales, Sutterellaceae, Burkholderiales, Erwiniaceae, Gammaproteobacteria, Akkermansiaceae, Verrucomicrobiales, Verrucomicrobiae* was identified, while in the microbiota of control mice, the family *Microbacteriaceae* was significantly more abundant (Fig. 3K). The phylogenetic investigation of communities by reconstruction of unobserved states (PICRUSt) based on OTUs was employed to forecast the abundances of functional categories. In the KEGG pathways, the microbial gene function related to Aging, NF-κB signaling pathway and Neurodegenerative disease were increased in the PD mice (Fig. 3L). Changes in these pathways indicate that the function of the gut microbiota has shifted toward the activation of aging and inflammation. Therefore, we further detected changes in intestinal inflammatory factors via enzyme-linked immunosorbent assay (ELISA). Surprisingly, the expression level of tumor necrosis factor-α (TNF-α) in the intestinal contents, plasma, and midbrain tissue of PD mice was significantly higher than that in control mice (Fig. 3M–O). We hypothesize that the inflammatory factors activated by the gut microbiota in PD mice enter the midbrain tissue through the blood circulation to exert their functions.

### Fecal microbiota transplantation (FMT) improves motor function and suppresses the release of TNF-α in PD mice

**Fig. 4:**
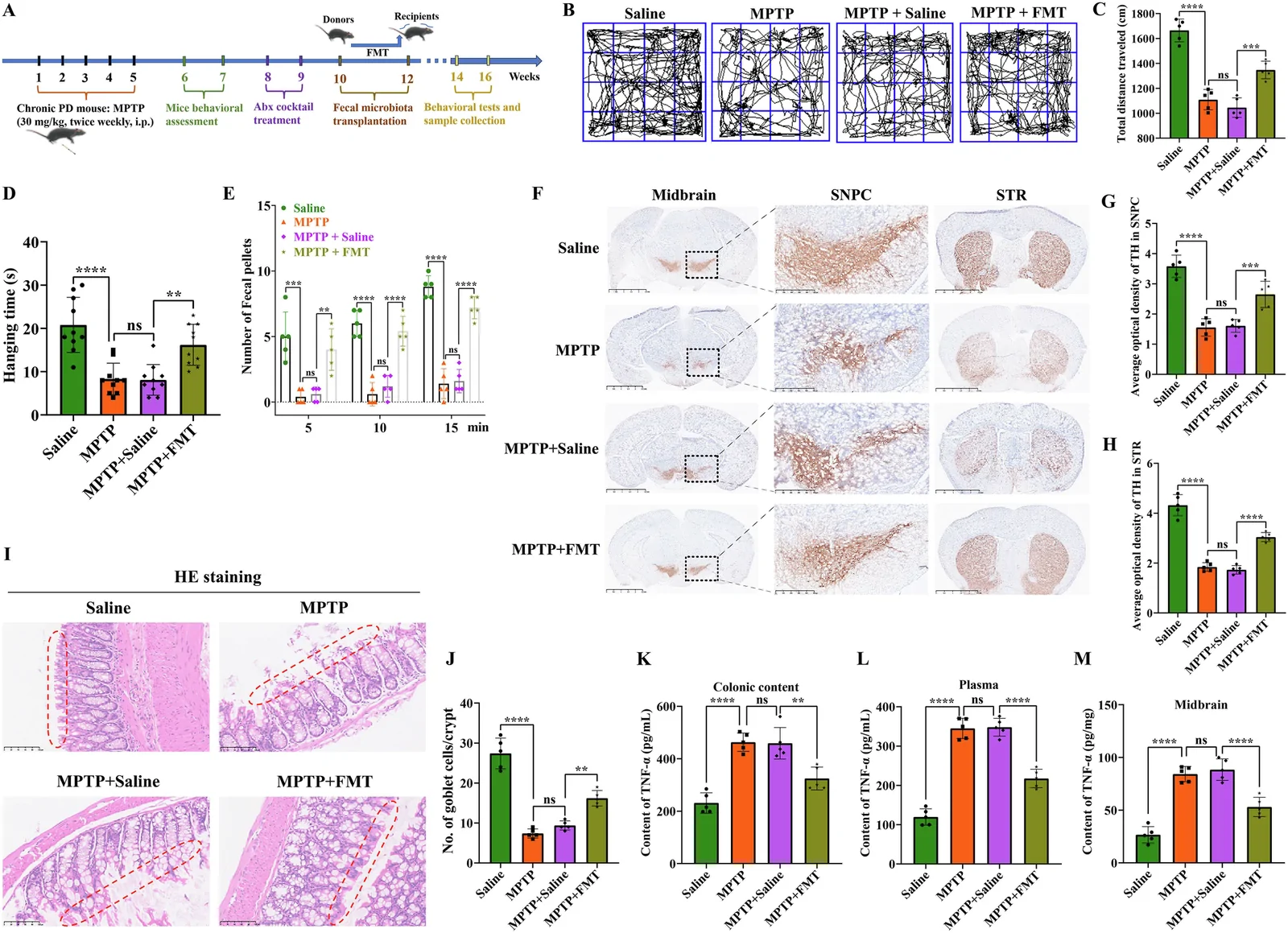
FMT improves motor function and enhances colonic mucosal integrity in PD mice. **A** Flowchart of PD mouse model establishment and FMT experiment. **B**, **C** Trajectory plots and statistical analysis graphs of the open field test. *n* = 5/group. **D** Statistical graph of the hanging test. *n* = 10/group. **E** Statistical graph of mouse feces collection. *n* = 5/group. **F**–**H** IHC staining and statistical analysis graphs of TH-positive cells in midbrain and striatum tissue. Scale bar: 2.5 mm or 500 µm. *n* = 5/group. **I**, **J** HE staining and statistical graphs of mouse colonic tissues. Scale bar: 100 µm. *n* = 5/group. Statistical graphs of TNF-α in mouse colonic contents (**K**), plasma (**L**), and midbrain tissues (**M**) detected by ELISA. *n* = 5/group. NS indicates no statistical significance. Data are represented as means ± SEM with ordinary one-way ANOVA. **P < 0.01, ***P < 0.001 and ****P < 0.0001.

To further elucidate the mechanistic role of gut microbiota dysbiosis in PD pathogenesis, we first established a chronic PD mouse model. Subsequently, we depleted the endogenous gut microbiota of these PD mice using an antibiotic cocktail and then performed fecal microbiota transplantation (FMT) to reconstitute their gut microbiota with that derived from healthy control mice (Fig. 4A). OFT assessment revealed that the total locomotor distance of PD mice was significantly reduced compared with that of control mice; notably, the total locomotor distance of PD mice receiving FMT (PD-FMT) was increased compared with the saline gavaged group (Fig. 4B, C). The results of the mouse hanging test showed that the hanging time of mice in the control group was significantly longer than that of PD mice, and the hanging time of PD-FMT was increased compared with that of the saline gavaged group (Fig. 4D). Meanwhile, the fecal pellet excretion of PD mice was significantly less than that of control mice, and the fecal pellet excretion of PD-FMT was higher than that of the saline gavaged group (Fig. 4E). IHC staining results indicated that compared with control mice, the number of TH-positive cells in the substantia nigra and striatum regions was significantly reduced in PD mice, while the number of TH-positive cells in PD-FMT was increased compared with that of the saline gavaged group (Fig. 4F–H). These results suggest that the motor ability of PD mice is improved and the number of dopaminergic neuron deaths is reduced after FMT.

PD mice exhibit constipation phenotypes that closely recapitulate those observed in clinical PD patients [16]. To investigate whether intestinal abnormalities exist in PD mice, we further performed Hematoxylin-Eosin (HE) staining on mouse colonic tissues. The results showed that the intestinal mucosal integrity and goblet cell abundance were more intact in control mice compared with PD mice, and PD-FMT mice had superior intestinal mucosal integrity and more abundant goblet cells than those in the saline gavage group (Fig. 4I, J). Additionally, ELISA results revealed that the expression of TNF-α was significantly increased in the colonic contents, plasma, and midbrain tissues of PD mice compared with control mice, while TNF-α expression was reduced in the colonic contents (Fig. 4K), plasma (Fig. 4L), and midbrain tissues (Fig. 4M) of PD-FMT mice compared with the saline gavage group. These findings suggest that PD mice receiving FMT have more intact intestinal mucosa and a greater number of goblet cells.

### Elevated expression of ATF4 in midbrain tissues of PD mice and ferroptotic PD cells

**Fig. 5:**
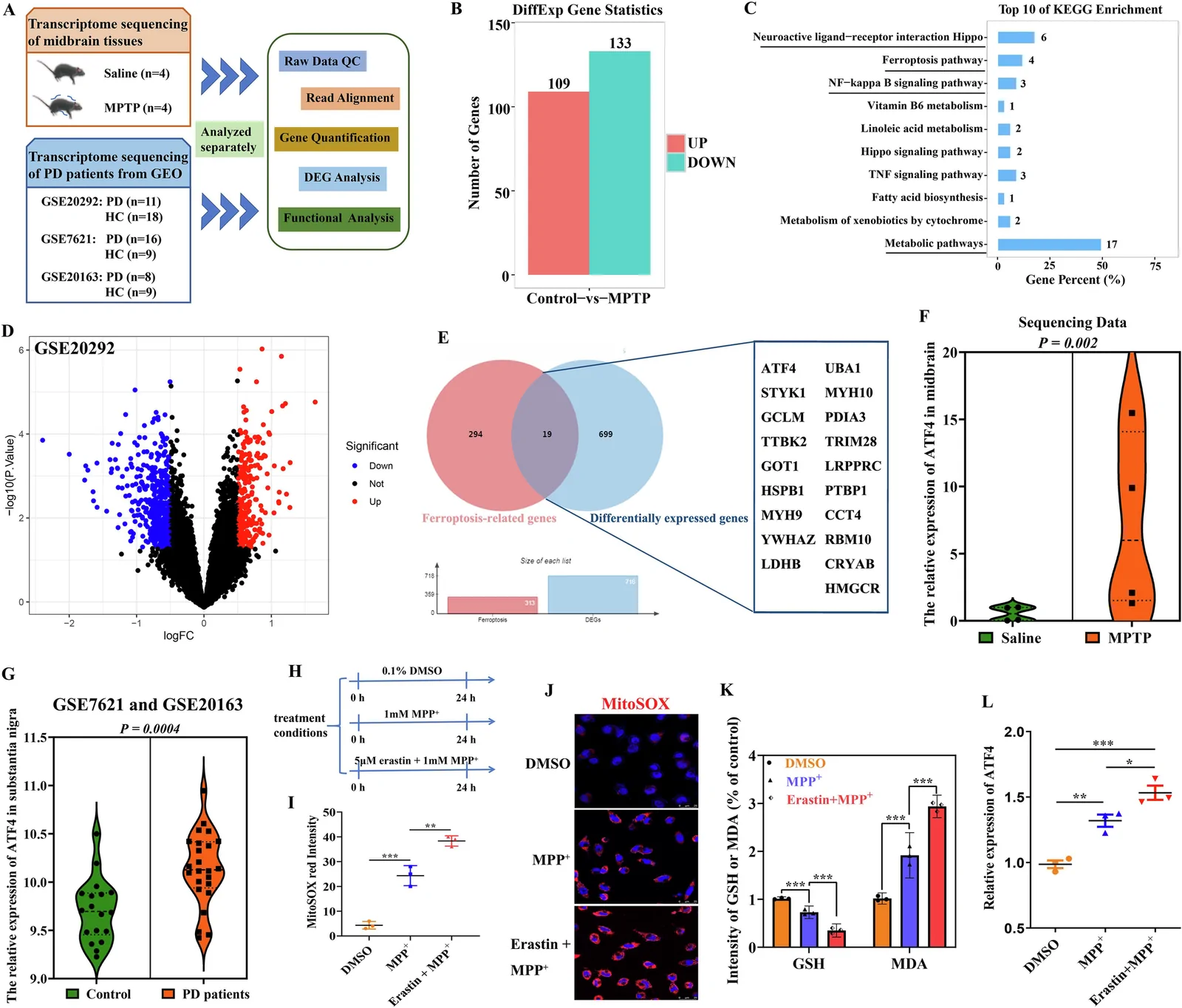
The high expression of *ATF4* gene in PD. **A** Schematic diagram of sequencing data and GEO dataset analysis. **B** Bar chart of differential gene analysis. **C** KEGG functional enrichment analysis of differential genes. **D** Volcano plot of differential gene analysis in the GSE20292 dataset. Transcripts annotated in red are upregulated, and those in blue are downregulated in PD patients relative to healthy controls. **E** Venn diagram showing ferroptosis-related differential genes. **F** Violin plot showing *ATF4* mRNA expression in sequencing data. **G** Expression analysis of *ATF4* mRNA in the GSE7621 and GSE20163 datasets. **H** Flowchart of cell treatment conditions. **I**, **J** Detection of mitochondrial oxidative levels in cells using the MitoSOX probe. Scale bar: 100 µm. **K** Analysis of GSH and MDA expression levels. **L** Detection of *ATF4* mRNA expression in cells from different treatment groups by RT-qPCR. Data are represented as means ± SEM with Student’s *t* test and ordinary one-way ANOVA (*n* = 3). *P < 0.05, **P < 0.01 and ***P < 0.001.

To elucidate the role of the gut-brain axis in PD pathogenesis, we first conducted transcriptomic sequencing on PD mouse midbrain tissues, and subsequently analyzed GEO database-retrieved transcriptomic data from the midbrain substantia nigra of clinical PD patients to identify dysregulated genes across animal and clinical PD samples (Fig. 5A). We performed transcriptomic sequencing analysis on midbrain tissues from mice, and differential expression gene (DEG) analysis revealed 109 upregulated and 133 downregulated genes in midbrain tissues of PD mice relative to control mice (Fig. 5B). KEGG functional enrichment analysis of these DEGs revealed that the main differential pathways included the ferroptosis pathway, NF-κB pathway (Fig. 5C). Numerous studies have demonstrated pathological iron deposition in midbrain tissues donated by PD patients, which is consistent with and corroborates the ferroptosis pathway enriched by DEGs from our transcriptome sequencing analysis [17]. Further analysis of the sequencing data of the substantia nigra in clinical patients from dataset GSE20292 identified 720 DEGs, including 260 upregulated and 460 downregulated genes (Fig. 5D). Subsequently, 313 ferroptosis-related genes (FRGs) were retrieved from the GeneCards database. Venn diagram intersection analysis of these 720 DEGs and 313 FRGs yielded 19 valid overlapping genes (Fig. 5E).

Among these genes, we found that the expression of activating transcription factor 4 (ATF4) was significantly different. Furthermore, extensive literature screening revealed that ATF4 plays a controversial role in ferroptosis regulation, as it exerts bidirectional effects, namely both ferroptosis-inhibitory and ferroptosis-promoting activities. In some degenerative osteoarthrosis, it has been demonstrated that ATF4 promotes ferroptosis and osteoarthritis (OA) progression by transactivating NUPR1, while ATF4 knockdown alleviates cartilage damage [18, 19]. However, no clear quantitative linear relationship between ATF4 expression levels and ferroptosis occurrence has been established, and research on ATF4-mediated ferroptosis in PD remains scarce. First, transcriptomic sequencing analysis of mouse midbrain tissues revealed elevated *ATF4* mRNA expression in PD mice relative to normal controls (Fig. 5F). Further analysis of the GSE7621 and GSE20163 datasets confirmed the upregulation of *ATF4* mRNA in the substantia nigra of clinical PD patients (Fig. 5G). Therefore, we further established a PD cell model using 1-Methyl-4-phenylpyridinium ion (MPP^+^), and treated MPP^+^ with erastin to induce further ferroptosis (Fig. 5H). Detection of mitochondrial oxidative levels using the MitoSOX fluorescent probe revealed that the PD cell model exhibited higher oxidative levels compared to the DMSO control group, while the PD cell + erastin treatment group showed even higher mitochondrial oxidation than MPP^+^ alone (Fig. 5I, J and Figure S1A, B). Further measurements of glutathione (GSH) and malondialdehyde (MDA) demonstrated that the PD cell had decreased GSH content and increased MDA expression level relative to the DMSO control group; similarly, the PD cell + erastin treatment group displayed a further reduction in GSH content and a further elevation in MDA expression level compared to the PD cell (Fig. 5K). RT-qPCR validation confirmed significantly higher *ATF4* mRNA expression in the PD cell model relative to the DMSO control group, with a further upregulation of this transcript observed in the PD cell model treated with erastin (Fig. 5L). These findings are intriguing, indicating that ATF4 exerts a fascinating regulatory mechanism in ferroptosis of PD cells.

### TNF-α induces ATF4 overexpression and DA neuronal ferroptosis in a concentration-and time-dependent manner

**Fig. 6:**
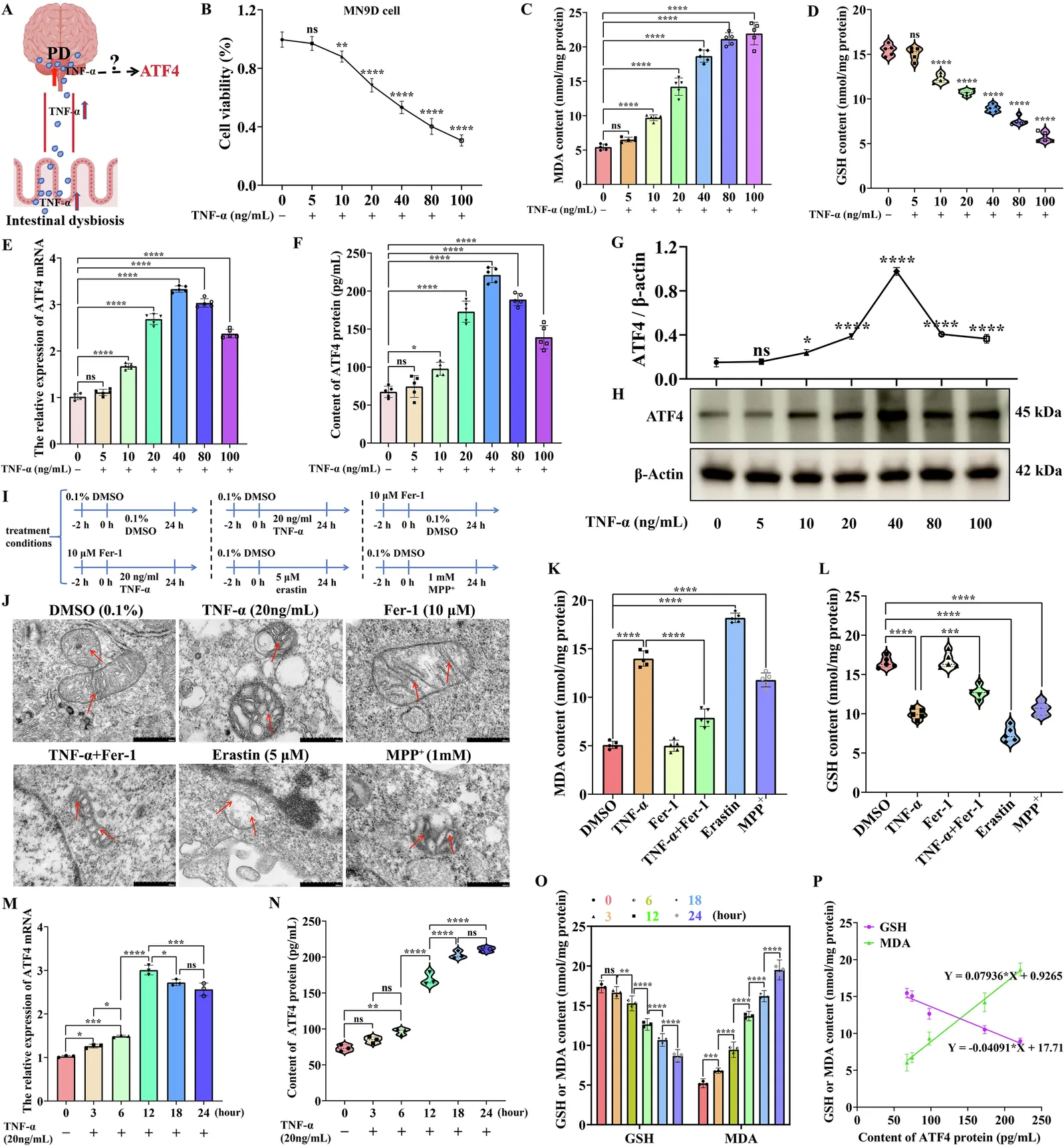
TNF-α promotes neuronal ferroptosis and induces the overexpression of ATF4. **A** Schematic diagram illustrating whether TNF-α upregulates ATF4 overexpression. **B** CCK-8 assay for evaluating the effect of TNF-α concentration gradient on neuronal viability. **C** Bar graph showing the effect of TNF-α concentration gradient on MDA content in dopaminergic neurons. **D** Violin plot depicting the effect of TNF-α concentration gradient on GSH content in dopaminergic neurons. **E** RT-qPCR analysis to detect the effect of TNF-α concentration gradient on *ATF4* mRNA levels in dopaminergic neurons. **F** ELISA for measuring the effect of TNF-α concentration gradient on ATF4 protein levels in dopaminergic neurons. **G**, **H** WB analysis to determine the effect of TNF-α concentration gradient on ATF4 protein levels in dopaminergic neurons. **I** Flowchart of cell treatment conditions. **J** TEM images showing mitochondrial morphological changes during TNF-α-induced neuronal ferroptosis. Scale bar: 500 nm. **K** Bar graph showing MDA content in dopaminergic neurons. **L** Violin plot depicting GSH content in dopaminergic neurons. **M** Bar graph showing the effect of 20 ng/mL TNF-α treatment at different time points on ATF4 mRNA levels in dopaminergic neurons. **N** Violin plot illustrating the effect of 20 ng/mL TNF-α treatment at different time points on ATF4 protein levels in dopaminergic neurons. **O** Line graphs showing the effects of TNF-α treatment at different time points on MDA and GSH contents. **P** Linear correlation analysis between ATF4 protein expression and MDA/GSH contents in dopaminergic neurons. Data are represented as means ± SEM with ordinary one-way ANOVA. *P < 0.05, **P < 0.01, ***P < 0.001 and ****P < 0.0001.

Based on our findings that the NF-κB pathway and ferroptosis pathway are activated in the midbrain of PD mice (Fig. 5C), combined with the elevated expressions of TNF-α and ATF4 observed in the midbrain tissues of these mice, we propose an intriguing question: Whether the increase in TNF-α induced can further promote the expression of ATF4 in PD (Fig. 6A). We investigated whether TNF-α-induced ferroptosis in dopaminergic neurons occurs in a concentration- dependent manner. Cell viability assays revealed a distinct concentration-dependent effect of TNF-α on MN9D cell viability: no significant change in cell survival rate was observed following treatment with 5 ng/mL TNF-α, a slight decrease at 10 ng/mL, a significant decline at 20 ng/mL, further reductions at 40 and 80 ng/mL, and the lowest survival rate at 100 ng/mL (Fig. 6B). Lipid peroxidation assays revealed that MDA content increased in a dose-dependent manner with escalating TNF-α concentrations: the MDA level in the control group was 5.8 ± 0.6 nmol/mg protein, elevated to 12.3 ± 1.2 nmol/mg protein following 20 ng/mL TNF-α treatment, peaked at 18.6 ± 1.7 nmol/mg protein at 40 ng/mL, and was further increased at 80 and 100 ng/mL TNF-α (Fig. 6C). GSH detection assays demonstrated that TNF-α treatment caused a significant dose-dependent reduction in intracellular GSH content: the GSH level in the control group was 15.2 ± 1.8 nmol/mg protein, decreased to 12.3 ± 1.2 nmol/mg protein at 20 ng/mL TNF-α, dropped to 9.7 ± 2.4 nmol/mg protein at 40 ng/mL, and declined further at 80 and 100 ng/mL (Fig. 6D). Furthermore, concentration-gradient experiments were performed to systematically investigate the inductive effect of TNF-α on ATF4 expression over a concentration range of 0–100 ng/mL, which revealed a distinct dose-dependent pattern of TNF-α-induced ATF4 expression. A slight upregulation of *ATF4* mRNA expression was observed at 5 ng/mL TNF-α; expression was significantly elevated at 10 ng/mL, reaching 1.8-fold that of the 0 ng/mL control group. *ATF4* mRNA expression was further increased to 2.7-fold the control level at 20 ng/mL and peaked at 40 ng/mL (3.2-fold vs. control), after which it slightly decreased at 80 and 100 ng/mL yet remained significantly higher than the control (Fig. 6E). The change trend of ATF4 protein expression was basically consistent with that of mRNA. ELISA quantification confirmed this finding, showing that ATF4 protein concentration was significantly higher than the control group after 24-hour treatment with 20 ng/mL TNF-α (Fig. 6F). Western blot results also demonstrated that TNF-α induced a gradual increase in ATF4 protein expression within the concentration range of 10–40 ng/mL, with the expression levels at 20 ng/mL and 40 ng/mL being 2.5-fold and 3.0-fold that of the control group, respectively (Fig. 6G, H).

To confirm that TNF-α-induced death of dopaminergic neurons occurs via ferroptosis rather than other forms of cell death, we performed ferroptosis-specific validation assays by supplementing with a ferroptosis inhibitor and a ferroptosis positive control (Fig. 6I). Transmission electron microscopy (TEM) analysis revealed intact cellular structure and normal mitochondrial morphology in dopaminergic neurons from the control group and the group pretreated with 10 μM Fer-1 for 2 hours alone. In contrast, mitochondria in dopaminergic neurons from the TNF-α-treated group, erastin-positive control group, and MPP⁺-induced PD cell model group displayed reduced volume, increased membrane electron density, and diminished cristae structure. Notably, Fer-1 pretreatment significantly attenuated the TNF-α-induced ferroptotic morphological phenotypes in dopaminergic neurons (Fig. 6J). MDA content assay revealed that the intracellular MDA level in the Fer-1 + TNF-α group was significantly lower than that in the TNF-α alone group (Fig. 6K). Furthermore, Fer-1 pretreatment remarkably elevated the GSH content reduced by TNF-α monotherapy (Fig. 6L). To investigate the temporal dynamics of TNF-α-induced ATF4 expression and ferroptosis in dopaminergic neurons, we conducted time-course kinetic experiments. Our results demonstrated that TNF-α-induced *ATF4* mRNA expression was detectable as early as 3 h, peaked at 12 h, and remained significantly elevated at 24 h (Fig. 6M). The upregulation of ATF4 protein was slightly delayed relative to its mRNA expression, with a significant increase starting at 12 h and peaking at 24 h (Fig. 6N). For ferroptosis-related markers, intracellular MDA content began to increase at 6 h and reached a maximum at 24 h, whereas intracellular GSH content started to decrease at 6 h with a significant reduction observed at 24 h (Fig. 6O). These findings demonstrate that TNF-α-induced ATF4 expression precedes the onset of ferroptosis, supporting an upstream regulatory role of ATF4 in the ferroptotic process. Linear correlation analysis revealed a significant correlation between ATF4 protein expression and ferroptosis-related markers within the TNF-α treatment concentration range of 0–40 ng/mL. Specifically, ATF4 expression was positively correlated with intracellular MDA content (*r* = 0.96, *P* < 0.0001) and negatively correlated with intracellular GSH content (*r* = 0.91, *P* < 0.0001). Further analysis showed that when ATF4 expression exceeded 2-fold that of the control group, significant alterations in ferroptosis-related markers of dopaminergic neurons were observed: intracellular MDA content increased to more than 3-fold the control level, while intracellular GSH content decreased to one-third of that in the control group (Fig. 6P). These findings suggest that the threshold concentration of ATF4 expression is approximately 2-fold higher than the control, beyond which dopaminergic neurons undergo significant ferroptosis.

### ATF4 activation induced by TNF-α further elicits ferroptosis in dopaminergic neurons

**Fig. 7:**
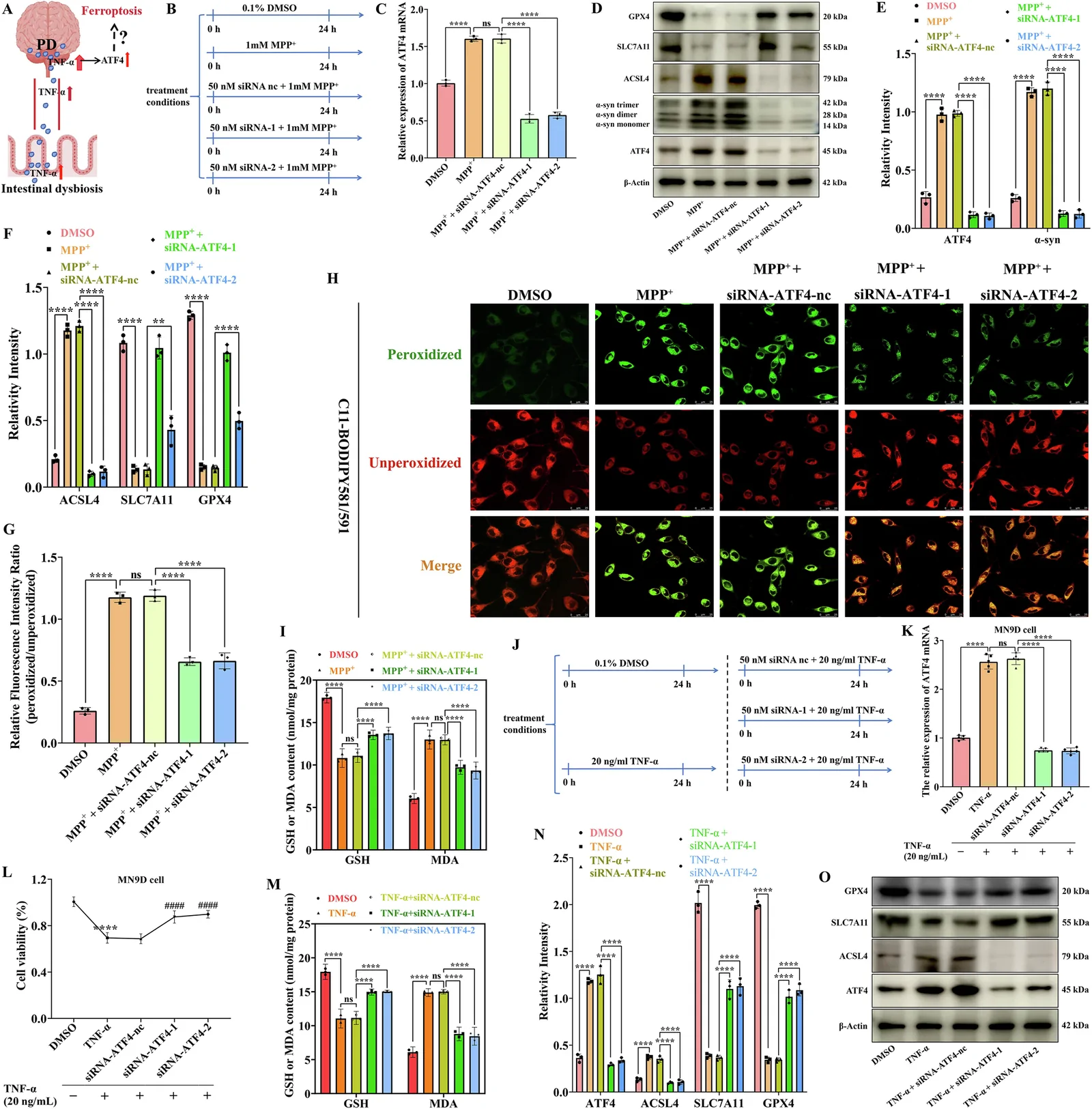
ATF4 is involved in the regulation of ferroptosis. **A** Schematic diagram illustrating TNF-α-mediated regulation of ferroptosis via ATF4. **B** Flow diagram of siRNA knockdown treatment conditions. **C** The expression of *ATF4* mRNA were analyzed by qRT-PCR. **D**–**F** WB analysis of the protein expression levels of GPX4, SLC7A11, ACSL4, α-syn and ATF4, along with the corresponding statistical graphs. **G**, **H** Representative fluorescence microscopy images and quantitative analysis depict intracellular lipid peroxidation dynamics using the C11-BODIPY581/591 probe. Unperoxidized lipids exhibit red fluorescence, whereas peroxidized lipids emit green fluorescence. Scale bar: 25 μm. **I** Statistical graphs of GSH and MDA content detection. **J** Flow diagram of siRNA-mediated knockdown combined with TNF-α treatment. **K** The expression of *ATF4* mRNA were analyzed by qRT-PCR. **L** CCK-8 assay for the evaluation of cell viability. **M** Statistical graphs of GSH and MDA content detection. **N**, **O** WB analysis of the protein expression levels of GPX4, SLC7A11, ACSL4 and ATF4, along with the corresponding statistical graphs. Data are represented as means ± SEM with ordinary one-way ANOVA. **P < 0.01 and ****P < 0.0001.

To investigate whether TNF-α promotes ferroptosis in dopaminergic neurons by upregulating ATF4 protein expression (Fig. 7A), we first performed ATF4 knockdown experiments using small interfering RNA (siRNA) specifically targeting ATF4 (Fig. 7B). RT-qPCR analysis revealed markedly elevated *ATF4* mRNA expression in the MPP⁺-induced PD cell model relative to the DMSO control group. When siRNA-ATF4-1/2 transfection was performed concomitantly with MPP⁺ treatment for 24 h, *ATF4* mRNA expression was significantly reduced compared with the siRNA-ATF4-NC group (Fig. 7C). Consistent with this, WB analysis revealed that ATF4 protein was remarkably reduced in cells transfected with siRNA-ATF4-1/2 relative to the siRNA-ATF4-NC group (Fig. 7D, E). Meanwhile, the expression of SLC7A11 and GPX4 proteins was elevated, while α-syn and ACSL4 protein expression was decreased in the siRNA-ATF4-1/2-transfected group compared with the siRNA-ATF4-NC group (Fig. 7D–F). Furthermore, we detected the intracellular lipid oxidation level of dopaminergic neurons using the C11- BODIPY581/591 fluorescent probe. The results demonstrated that the oxidation level was significantly higher in the PD cell than in the DMSO group, and was reduced in the siRNA-ATF4-1/2 compared with the siRNA-ATF4-nc group (Fig. 7G, H; Figure S1C, D). Detection of intracellular GSH content showed a decrease in the PD cell relative to the DMSO group, while siRNA-ATF4-1/2 significantly increased GSH content compared with the siRNA-ATF4-nc group. In contrast, the intracellular MDA content exhibited an opposite trend to that of GSH (Fig. 7I).

Previously, our concentration-gradient screening identified that treatment with 20 ng/mL TNF-α for 24 hours effectively activates ATF4 expression and triggers ferroptosis in dopaminergic neurons. Building on this finding, we transfected dopaminergic neurons with siRNA-ATF4-1/2 to knockdown ATF4 while exposing them to 20 ng/mL TNF-α (Fig. 7J). RT-qPCR analysis revealed that *ATF4* mRNA expression was significantly elevated in TNF-α-treated cells relative to the DMSO control group; notably, in TNF-α-stimulated cells, transfection with siRNA-ATF4-1/2 markedly reduced *ATF4* mRNA levels compared with the siRNA-ATF4-NC group (Fig. 7K). CCK-8 cell viability assay showed that TNF-α treatment diminished cell viability compared with DMSO, while transfection with siRNA-ATF4-1/2 significantly restored cell viability in TNF-α-activated cells relative to siRNA-ATF4-NC (Fig. 7L). Assay kit-based detection revealed that TNF-α-stimulated cells exhibited significantly higher intracellular MDA content than the DMSO control group, and this elevation was attenuated by ATF4 knockdown via siRNA-ATF4-1/2 compared with the siRNA-ATF4-NC group. In contrast, intracellular GSH content showed an opposite trend to that of MDA (Fig. 7M). Consistent with these findings, WB analysis demonstrated a marked reduction in ATF4 protein levels in TNF-α-treated cells transfected with siRNA-ATF4-1/2 relative to the siRNA-ATF4-NC group. Concomitantly, in siRNA-ATF4-1/2-transfected TNF-α-stimulated cells, SLC7A11 and GPX4 protein expression was upregulated, while ACSL4 protein expression was downregulated compared with the siRNA-ATF4-NC group (Fig. 7N, O). Taken together, these results demonstrate that TNF-α promotes ferroptosis in dopaminergic neurons by upregulating ATF4 expression.

### TNF-α drives ATF4 overexpression through activating the TNFR1-NF-κB axis

**Fig. 8:**
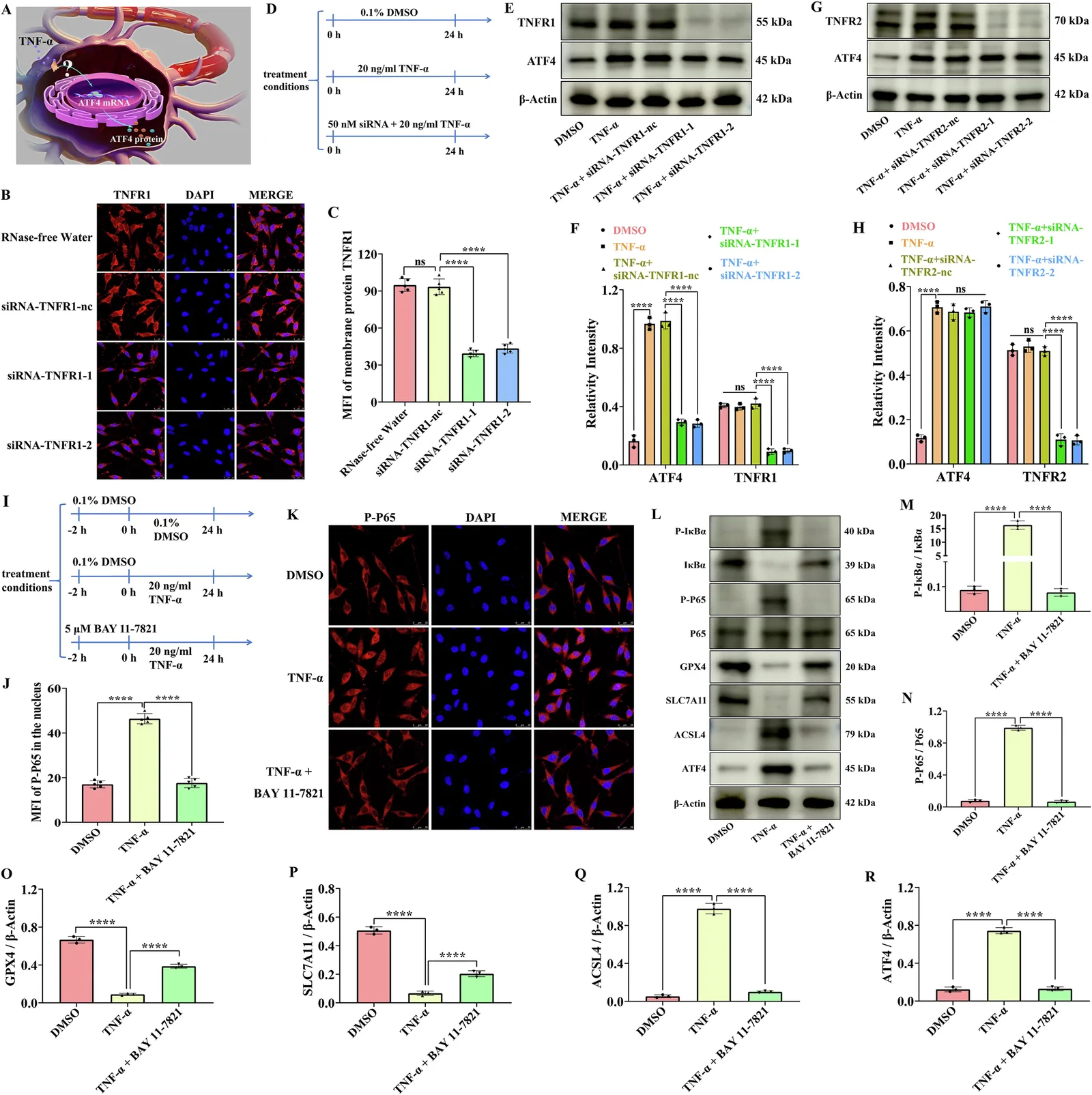
TNF-α upregulates high ATF4 expression via the NF-κB signaling pathway. **A** Schematic diagram of the TNF-α regulatory mechanism. **B**, **C** IF detection of TNFR1 protein expression on the cell membrane and statistical analysis of its fluorescent signal. Scale bar: 25 μm. **D** Flow diagram of siRNA knockdown treatment conditions. **E**, **F** WB analysis of ATF4 protein expression changes in TNFR1-knockdown cells and statistical analysis of the protein level. **G**, **H** WB analysis of ATF4 protein expression changes in TNFR2-knockdown cells and statistical analysis of the protein level. **I** Flow diagram of NF-κB inhibitor combined with TNF-α treatment conditions. **J**, **K** IF detection of P-P65 nuclear translocation in cells and statistical analysis of nuclear P-P65 protein level. Scale bar: 25 μm. **L**–**R** WB analysis of protein expression changes in the NF-κB and ferroptosis signaling pathways and statistical analysis of related proteins. Data are represented as means ± SEM with ordinary one-way ANOVA. ****P < 0.0001.

We further elucidated the mechanism by which TNF-α upregulates the high expression of ATF4, thereby inducing ferroptosis in dopaminergic neurons (Fig. 8A). Given that secreted TNF-α exerts its biological functions primarily through activating the membrane receptor tumor necrosis factor receptor 1 (TNFR1) [20], we first performed receptor knockdown via siRNA transfection. RT-qPCR analysis revealed that siRNA-1/2 significantly reduced the mRNA expression levels of both *TNFR1* and tumor necrosis factor receptor 2 (*TNFR2*) compared with the siRNA-NC group (Figure S2A, B). Consistent with this transcriptional downregulation, WB analysis confirmed that siRNA-1/2 transfection also decreased the protein expression of TNFR1 and TNFR2 (Figures S2C–F). Immunofluorescence (IF) assays further confirmed that membrane-localized TNFR1 protein was markedly downregulated in siRNA-TNFR1-1/2-transfected cells relative to the siRNA-TNFR1 -NC group (Fig. 8B, C). To identify the specific receptor mediating ATF4 upregulation, we examined ATF4 protein expression in TNFR1- or TNFR2- knockdown cells (Fig. 8D). WB analysis revealed that ATF4 protein expression was significantly reduced in TNFR1-knockdown cells relative to the control group (Fig. 8E, F); in contrast, no notable changes in ATF4 protein expression were observed in TNFR2-knockdown cells (Fig. 8G, H). These data collectively demonstrate that TNFR1, but not TNFR2, is required for TNF-α-induced ATF4 upregulation in dopaminergic neurons. It is well established that TNF-α binding to TNFR1 activates multiple intracellular signaling pathways, among which the NF-κB and MAPK pathways are the most extensively characterized [21]. In our previous DEG enrichment analysis, functional annotation revealed a significant enrichment of DEG in the NF-κB signaling pathway (Fig. 5C), which prompted us to investigate the activation status of the NF-κB pathway. Therefore, we added an NF-κB pathway inhibitor (BAY 11-7821) 2 h prior to TNF-α treatment to block the pathway, and detected changes in downstream signaling pathways and protein expression (Fig. 8I). IF staining analysis revealed that phosphorylated P65 (p-P65)—a key phosphorylated effector of NF-κB pathway activation—exhibited a marked increase in expression and pronounced nuclear translocation upon TNF-α treatment. These TNF-α-induced effects were completely abrogated by pretreatment with BAY 11-7821 (Fig. 8J, K and Figure S2G, H). To further confirm the role of NF-κB signaling in TNF-α-mediated ferroptosis, we analyzed the expression of key pathway components and ferroptosis-related proteins via WB. As shown in Fig. 8L, TNF-α treatment significantly upregulated the protein expression of P-IκBα, P-P65, ACSL4, and ATF4 in dopaminergic neurons compared with the control group. In contrast, addition of BAY 11-7821 led to a marked decrease in the expression of P-IκBα, P-P65, ACSL4, and ATF4, accompanied by a concurrent increase in the expression of GPX4 and SLC7A11 (Fig. 8L–R). These results indicate that the NF-κB pathway acts as a critical mediator linking TNF-α-TNFR1 signaling to ATF4 upregulation and subsequent ferroptosis in dopaminergic neurons.

We further investigated alterations in the ferroptosis pathway in the midbrain tissues of mice. WB analysis revealed that, compared with the control group, the MPTP group exhibited decreased protein expression of TH, GPX4 and SLC7A11, accompanied by a significant upregulation of α-syn, ACSL4 and ATF4 in the midbrain. In contrast, MPTP-induced PD mice receiving FMT showed elevated expression of TH, GPX4 and SLC7A11, as well as reduced expression of α-syn, ACSL4 and ATF4 in the midbrain relative to PD mice treated with normal saline (Figure S3A–D). Additionally, measurements of GSH and MDA levels in the midbrain demonstrated that the MPTP group had lower GSH content and significantly higher MDA content than the control group, whereas FMT treatment increased GSH content and decreased MDA content in PD mice (Figure S3E). Collectively, these in vivo findings confirm that FMT ameliorates MPTP-induced ferroptosis in the mouse midbrain by modulating the expression of key ferroptosis-related molecules and rectifying abnormal redox homeostasis.

## Discussion

For decades, research into the pathogenesis of PD has been predominantly centered on the CNS [22]. However, with breakthroughs in research technologies and the evolution of conceptual frameworks, an increasing number of investigations have shifted their focus from the CNS to peripheral tissues and systems, with dysregulation of the gut microbiota and its metabolites emerging as a highly promising and intensely studied new frontier in PD pathogenesis research [23, 24]. This paradigm shift is strongly supported by compelling clinical evidence: a symptomatic analysis of clinical PD patients has revealed that gastrointestinal symptoms such as constipation occur a decade or even longer prior to the onset of motor symptoms, accompanied by pathological alterations of α-synuclein in the enteric vagal nervous system—a finding that provides robust evidence for a gut-originated pathogenic model of PD [25]. This study, via a multi-dimensional investigation encompassing genetics, microbiota, and cell biology, unravels a distinct pathogenic cascade: gut microbiota dysbiosis, as a peripheral perturbation, transmits signals across the gut-brain axis to induce ferroptosis in dopaminergic neurons through the TNF-α-NF-κB-ATF4 pathway. In addition to identifying a novel molecular mechanism underlying this pathological process, these findings also prompt critical reflections on the interconnectedness of biological systems, the dialectics of physiological balance and imbalance, and the epistemological shift in the understanding of complex multifactorial diseases.

In this study, using rigorous GWAS data and stringent SNP screening, we identified two gut microbiota taxa, *Clostridium sensu stricto 1* and *Candidatus Soleaferrea*, as critical in PD pathogenesis. Reliability was ensured by high-quality GWAS data and comprehensive sensitivity analyses (no pleiotropy, low heterogeneity, no publication bias). This aligns with the holistic philosophical view that living organisms are not just simple collections of individual parts, but integrated whole systems, where changes in one peripheral part can spread through interconnected networks and affect the entire body [26]. These taxa have well-documented regulatory functions supporting their PD relevance. *Clostridium* species typically maintain intestinal homeostasis and mitigate inflammation via cellular components and metabolites [27]. However, our MR analysis reveals that genetic variants of *Clostridium sensu stricto 1* constitute high-risk factors for PD. The pathogenic mechanism of this bacterium may trigger intestinal pro-inflammatory responses in PD models, indicating PD-linked functional dysregulation. *Candidatus Soleaferrea* is a differential genus in Alzheimer’s disease (AD)-related mild cognitive impairment (MCI), with higher abundance in MCI subjects; it regulates neuroinflammation and cognitive dysfunction in AD, a neurodegenerative disease with pathological similarities to PD, implying analogous roles in PD [28]. The gut microbiota-PD bidirectional interaction further emphasizes these taxa’s core roles. A landmark *Cell* study confirmed gut microbiota are essential for PD-related motor deficits, microglial activation, and α-synuclein pathology, supporting their primary causal role in PD [29]. While PD can alter gut microbiota (e.g., reduced *Faecalibacterium* and *Ruminococcus* in patients), this reverse regulation is secondary to gut microbiota’s dominant causal effect on PD progression [30].

Combined with our core discoveries, this result further clarifies that gut microbial dysbiosis turns the gut into a critical pathogenic node in PD. The gut does not act in isolation; instead, it acts as a peripheral perturbation that initiates signals across the gut-brain axis, ultimately triggering dopaminergic neuron ferroptosis via the TNF-α-NF-κB-ATF4 pathway. This all reminds us that health and disease are not just the result of isolated defects in a single organ, but emergent properties shaped by the complex interactions of the entire bodily system. The gut-brain axis facilitates communication between the gut and the CNS via inflammatory mediators, microbial metabolites, and neural pathways [31]. Our study identified TNF-α as a critical messenger in this axis: gut microbiota dysbiosis induces TNF-α elevation in the intestine, plasma, and midbrain, which subsequently triggers dopaminergic neuron ferroptosis. A key limitation of our study is the lack of direct evidence that midbrain TNF-α in PD mice is gut-derived and translocates to the CNS. We further identified that FMT normalized TNF-α levels in the intestine, plasma, and midbrain. This correction strongly suggests the gut microbiota drives midbrain TNF-α accumulation, implying gut-to-brain translocation. The feasibility of TNF-α crossing the blood-brain barrier (BBB) to the midbrain is supported by literature. Inflammation disrupts BBB integrity, enabling circulating TNF-α to infiltrate the CNS. Hammed et al. showed that intestinal injury impairs intestinal barrier function, allowing intestinal-derived TNF-α to enter circulation, disrupt the BBB, and reach the brain [32]. Gutierrez et al. used ¹²⁵I-mTNFα labeling to demonstrate murine TNF-α crosses the BBB intact via a saturable transport system [33]. Doney et al. found intestinal dysbiosis-induced inflammation releases TNF-α, which translocates to the brain via increased BBB permeability [34]. These studies support our hypothesis that gut-derived TNF-α contributes to midbrain TNF-α accumulation in PD. We do not exclude local brain TNF-α production; however, FMT-mediated midbrain TNF-α correction strongly supports gut microbiota contribution to central TNF-α accumulation. To confirm midbrain TNF-α’s gut origin, future studies will use direct tracing (e.g., isotope labeling) to track gut-derived TNF-α translocation to the midbrain, quantify its contribution, and validate the gut-brain axis in PD-related neuroinflammation.

This finding aligns with previous research showing elevated TNF-α levels in PD patients and animal models [35], but extends these observations by elucidating the downstream molecular cascade linking TNF-α to neurodegeneration. Our study builds on this by identifying ATF4 as a novel downstream target of TNF-α-NF-κB signaling in PD. We would like to clarify that ATF4 is a multifunctional transcription factor whose biological effects are highly context-dependent—its role can vary with cell type, disease model, and activation level, which may explain the differences between our findings and some existing studies [14, 36]. For example, we acknowledge that ATF4 has been reported to exert protective effects in some tumor research fields, such as its role in the ATF4/CEMIP/PKCα axis that promotes anoikis resistance by enhancing protective autophagy in prostate cancer cells [37]. However, in PD, especially under persistent TNF-α activation, ATF4 is abnormally overexpressed (3.2-fold and 3.0-fold of control at mRNA and protein levels, respectively), shifting its function from protective to harmful. Specifically, other relevant studies have indicated that ATF4 is upregulated in osteoarthritis (OA) treated with interleukin-1β (IL-1β) and promotes ferroptosis, while ATF4 knockdown ameliorates cartilage damage in both cell and mouse models, thereby identifying the ATF4-NUPR1 axis as a critical therapeutic target for OA [18]. Another study showed sevoflurane induces glioma cell ferroptosis via the ATF4-CHAC1 pathway [38], confirming ATF4 as a pro-ferroptotic regulator across cell types and contexts, which supports our conclusion in PD. This study reveals that sustained ATF4 activation downstream of the integrated stress response (ISR) triggered by PD-related neurotoxins and α-synuclein fibrils upregulates pro-apoptotic genes to facilitate dopaminergic neuronal death, whereas inhibiting ATF4-related signaling alleviates such neuronal injury, identifying ATF4 as a promising therapeutic target for PD [39].

Our experimental data confirm that ATF4 mediates TNF-α-induced ferroptosis in dopaminergic neurons. Time-course experiments showed TNF-α-induced ATF4 expression (detectable at 3 h, peaking at 12–24 h) precedes ferroptosis (MDA increase and GSH decrease starting at 6 h, peaking at 24 h), indicating ATF4 acts upstream of ferroptosis. Importantly, we identified an ATF4 threshold effect: expression >2-fold of control caused ferroptosis (MDA > 3-fold, GSH < 1/3 of control). Loss-of-function experiments with ATF4-specific siRNA showed that ATF4 knockdown reversed TNF-α-induced ferroptosis, confirming ATF4 as a critical link between TNF-α signaling and ferroptosis in dopaminergic neurons.

We acknowledge a limitation: the lack of direct proof that ATF4 controls the mentioned ferroptosis-related genes. Our study confirms a strong correlation between ATF4 and ferroptosis, supported by associations with markers (GPX4, SLC7A11, ACSL4, GSH, MDA), temporal sequence, and concentration-dependent upregulation of ATF4 and ferroptosis. However, direct regulation of downstream genes by ATF4 (e.g., via promoter binding) requires further investigation, which we plan to supplement in future studies. This finding highlights the complexity of ATF4 function in neurodegeneration and provides a new perspective on cell death mechanisms in PD. Furthermore, our study demonstrates that TNF-α specifically binds to TNFR1 (not TNFR2) to activate NF-κB, as TNFR1-knockdown abrogates ATF4 upregulation and ferroptosis. This receptor-specificity is crucial for therapeutic development, as targeting TNFR1 could selectively inhibit the pathogenic pathway without affecting the protective functions of TNFR2.

Ferroptosis, an iron-dependent form of programmed cell death, has been implicated in various neurodegenerative diseases, including PD [40]. These studies outlined the key regulators of ferroptosis, including GPX4, SLC7A11, and ACSL4, and their involvement in DA neuronal death [41]. Our transcriptomic analysis identified ferroptosis as a key enriched pathway in both PD mouse midbrain, with ATF4 significantly upregulated. Mechanistically, we showed that ATF4 overexpression (exceeding 2-fold of control levels) acts as a switch to trigger ferroptosis, as evidenced by GSH depletion, MDA accumulation, and impaired GPX4/SLC7A11 expression. This ATF4-mediated ferroptosis pathway complements the existing regulatory network of ferroptosis in PD. Previous studies focused on mitochondrial dysfunction and iron metabolism disorders, but our study reveals an inflammation -driven ferroptosis mechanism initiated by gut microbiota dysbiosis [42]. The integration of these findings suggests that PD pathogenesis involves multiple ferroptosis-inducing pathways, and targeting ATF4 could block the inflammation -mediated branch. Fecal microbiota transplantation (FMT), which reshapes the gut microbiota, has emerged as a potential therapeutic strategy for PD; a study demonstrated that FMT exerts a more effective improvement on motor function in early PD mice [43]. Our study provides a mechanistic basis for these observations: FMT from healthy mice restores gut microbial homeostasis, reduces TNF-α levels, inhibits the NF-κB-ATF4 signaling pathway, and thereby protects dopaminergic neurons from ferroptosis. This indicates that FMT exerts its therapeutic effects in PD not only by restoring beneficial gut microbiota but also by directly suppressing the pathogenic inflammation-ferroptosis cascade.

Despite its strengths, this study has several limitations. First, the MR analysis was based on existing GWAS datasets, and the number of instrumental variables for each microbial genus was relatively small. Future studies with larger GWAS samples and more comprehensive gut microbiota profiling will improve the robustness of the causal inference. Second, the MPTP-induced PD model is one of the most widely used and well-validated models in PD research, yet it may not fully recapitulate the complexity of human PD [44]. Our study confirmed that this model exhibits intestinal barrier dysfunction and gut inflammation, in line with previous reports indicating that MPTP causes gastrointestinal lesions and gut microbiota dysbiosis preceding motor dysfunction [45, 46]. Our findings will be further validated in other PD models such as SNCA*A53T mice in future studies. Third, MN9D cells are capable of producing dopamine and expressing tyrosine hydroxylase and aromatic amino acid decarboxylase. Numerous studies have validated the use of MN9D cells for investigating mechanisms underlying dopaminergic neuronal loss and evaluating potential therapeutic strategies in PD. However, as an immortalized cell line, MN9D cells cannot fully recapitulate the physiological characteristics of primary midbrain dopaminergic neurons. Further investigations using primary neuronal cultures will be conducted in future studies to validate and extend our mechanistic findings [47, 48]. Fourth, the specific metabolites of *Clostridium sensu stricto 1* and *Candidatus Soleaferrea* that induce TNF-α elevation remain unclear. In future work, we will perform metabolomic analysis of these bacteria to identify novel pathogenic metabolites and potential therapeutic targets.

Overall, this study identifies a previously unrecognized causal mechanism by which gut microbiota dysbiosis contributes to dopaminergic neuron ferroptosis in PD. Specifically, disrupted gut microbiota homeostasis elevates TNF-α levels, thereby triggering TNFR1-NF-κB signaling activation and subsequent ATF4 upregulation, which ultimately promotes neuronal ferroptosis. This finding bridges the gap between peripheral microbial perturbations and central neurodegeneration, provides new insights into PD pathophysiology, and identifies potential therapeutic targets and strategies. The integration of MR analysis, animal models, cellular experiments, and transcriptomic profiling ensures the robustness of our findings, which may pave the way for the development of novel diagnostic and therapeutic approaches for PD.

## Materials and methods

### Study design and ethical approval

This study integrated MR analysis, animal experiments, cellular assays, and bioinformatics to dissect the gut-brain axis-mediated mechanism in PD. All animal experiments were carried out in accordance with animal ethics and were approved by Peking University Shenzhen Hospital and the animal center of Shenzhen Peking University-The Hong Kong University of Science and Technology Medical Center (No.00385362) and conducted in compliance with the Guide for the Care and Use of Laboratory Animals. More detailed experimental procedures, as well as the grouping design of cell and animal experiments, are provided in the supporting information.

### Animals and PD-model establishment

C57BL/6 J mice (male, 8 weeks) were purchased from Vitalriver (Beijing Vital River Laboratory Animal Technology Co., Ltd.). All mice were kept on a standard 12:12-h light–dark cycle, with controlled temperature (21–25 °C) and humidity (40–60%), and with freely accessible food and water. A chronic PD mice model was given 30 mg/kg 1-methyl-4-Phenyl-1,2,3,6-tetrahydropyridine (MPTP) (Biorbyt, #orb363933-1g) twice a week for 5 weeks by intraperitoneal injection, while the control group was injected with saline [49].

### Fecal microbiota transplantation (FMT)

Following the completion of PD mouse modeling, antibiotic treatment was administered. PD mice were treated with an antibiotic cocktail (neomycin 0.5 g/L, gentamicin 100 mg/L, ampicillin 1 g/L, vancomycin 0.5 g/L, and erythromycin 10 mg/L) in drinking water for 7 days to deplete gut microbiota [29]. Afterwards, fresh feces from healthy C57BL/6 mice were suspended in sterile PBS (100 mg feces/mL), homogenized, and centrifuged at 900 × *g* for 5 min. The supernatant (150 μL) was gavaged into antibiotic-pretreated PD mice (PD-FMT group) every other day for 2 weeks [50]. The PD-saline group received sterile PBS gavage.

### Behavioral tests

Open field test: Mice were placed in an open field arena (50 × 50 × 40 cm) and allowed to explore freely for 10 min. Total locomotor distance was recorded using a video camera and analyzed using behavior tracking software. Hanging test: All mice underwent adaptive training for three consecutive days to acclimate to the testing environment and procedures prior to the formal assay. For the test, mice were first suspended by their forelimbs at the center of a metal wire, and the latency to fall off the metal wire was recorded [49]. Fecal pellet count: Mice were individually housed in clean cages for a 15-min period, and fecal pellet production was counted at 5-min intervals.

### Microarray datasets

The gene expression microarrays of GSE20292 [51], GSE7621 [52] and GSE20163 [53] were acquired from the GEO database of the National Center for Biotechnology Information (NCBI). The details of those datasets can be found in Table 2.

**Table 2:** Basic information of GEO datasets.

Series name	Platform	Samples	Organization Source
GSE20292	GPL96	Parkinson’s disease: 11 Healthy people: 18	Substantia nigra
GSE7621	GPL570	Parkinson’s disease: 16 Healthy people: 9	Substantia nigra
GSE20163	GPL96	Parkinson’s disease: 8 Healthy people: 9	Substantia nigra

### RNA extraction and sequencing

Total RNA was extracted from mouse midbrain tissues using TRIzol reagent in accordance with the manufacturer’s protocol. RNA quality and integrity were evaluated using a NanoDrop 2000 spectrophotometer (Thermo Fisher Scientific) and an Agilent 2100 Bioanalyzer (Agilent Technologies), respectively. Transcriptome sequencing was performed on an Illumina next-generation sequencing platform (Illumina Inc., San Diego, CA, USA). Complementary DNA, DNA, and small RNA libraries were constructed and sequenced on the Illumina platform by Genedenovo Biotechnology Co., Ltd. (Guangzhou, China). Subsequent data analysis after sequencing was conducted by Genedenovo Biotechnology Co., Ltd, and the detailed analysis workflow and methods are provided in the supporting information.

### MN9D cells culture

MN9D cells were cultured in Dulbecco’s Modified Eagle Medium (DMEM, GIBCO) containing 10% fetal bovine serum (GIBCO), 1% penicillin with streptomycin (Beyotime Biotechnology) in an incubator at 37 °C and 5% CO_2_. MN9D cells (a mouse mesencephalic dopaminergic cell line) were purchased from American Type Culture Collection (ATCC).

### Cell model establishment

PD cell model: Cells were treated with 1 mM 1-methyl-4-phenylpyridinium ion (MPP⁺, Sigma-Aldrich, #D048-1G) for 24 h to induce PD-like phenotypes. Cells were treated with 5 μM erastin (MedChemExpress, #HY-15763) or TNF-α (MedChemExpress, #HY-P7058) for 24 h to induce ferroptosis. Inhibition experiments: Cells were pretreated with 10 μM Fer-1 (ferroptosis inhibitor, MedChemExpress, #HY-100579) or 5 μM BAY 11-7821 (NF-κB inhibitor, MedChemExpress, #HY-13453) for 2 h before TNF-α treatment. A combined treatment of MPP⁺and erastin for a total duration of 24 hours was administered to robustly induce ferroptosis in PD-model cells and validate the expression of corresponding genes.

### Statistical analysis

All statistical analyses were performed using GraphPad Prism 9.0 software and R 4.2.0. Data are presented as mean ± standard error of the mean (SEM). The normality of the data was first evaluated using the Shapiro-Wilk test. For data that satisfied the normality assumption, comparisons between two groups were conducted using the two-tailed unpaired Student’s *t* test, while multiple group comparisons were analyzed by one-way or two-way repeated measurement analysis of variance (ANOVA) followed by Tukey’s post-hoc test. For data that did not meet the normality assumption, non-parametric tests were applied: the Mann–Whitney-*U* test was used for comparisons between two groups, and the Kruskal–Wallis test followed by Dunn’s post-hoc test was used for multiple group comparisons. Linear correlation analysis (Pearson’s test) was performed to assess the relationship between ATF4 expression and ferroptosis indicators. *P* < 0.05 was considered statistically significant. *P < 0.05, **P < 0.01, ***P < 0.001, ****P < 0.0001. Each experiment was repeated independently at least three times (*n* ≥ 3).

## Supplementary information

Supporting Information

western blots

## Data Availability

The analysis data mentioned in the study are included in the supplementary materials of the article, which have been uploaded to the journal’s official submission system and verified to be live and fully accessible. Further inquiries regarding the data can be directed to the corresponding author.
