# Supplementary material for: Gut microbiota-derived TNF-α triggers dopaminergic neuron ferroptosis via TNFR1-NF-κB-ATF4 axis in Parkinson’s disease
Source: Cell Death Discov. 2026 Aug 3;12:433. doi: 10.1038/s41420-026-03281-x (PMC13720208; doi:10.1038/s41420-026-03281-x)
Supplement: Supplementary file 1 [file 41420_2026_3281_MOESM1_ESM.pdf]

Supporting information

Gut microbiota-derived TNF-α triggers dopaminergic neuron ferroptosis via TNFR1-NF-κB-ATF4 axis in Parkinson's Disease

Zixia Zhang[3,#], Sufang Zhong[1,2], Liangliang Gao[1,2], Baodong Chen[1,2,*], Peng Huang[1,2,#,*]

[1] Department of Neurosurgery, Peking University Shenzhen Hospital, Shenzhen Peking University-The Hong Kong University of Science and Technology Medical Center, Shenzhen 518000, Guangdong, P. R. China.

[2] Department of Neurosurgery, Peking University Shenzhen Hospital, Shenzhen 518000, Guangdong, P. R. China.

[3] Department of Hematology, South China Hospital of Shenzhen University, Shenzhen 518000, Guangdong, P. R. China.

[#]contributed equally.

[*]Corresponding authors:

Baodong Chen, e-mail: bdchen@pkuszh.com;

Peng Huang, email: 327203498@qq.com.

Supplementary figures:

Figure S1. Detection of cellular lipid oxidation levels.

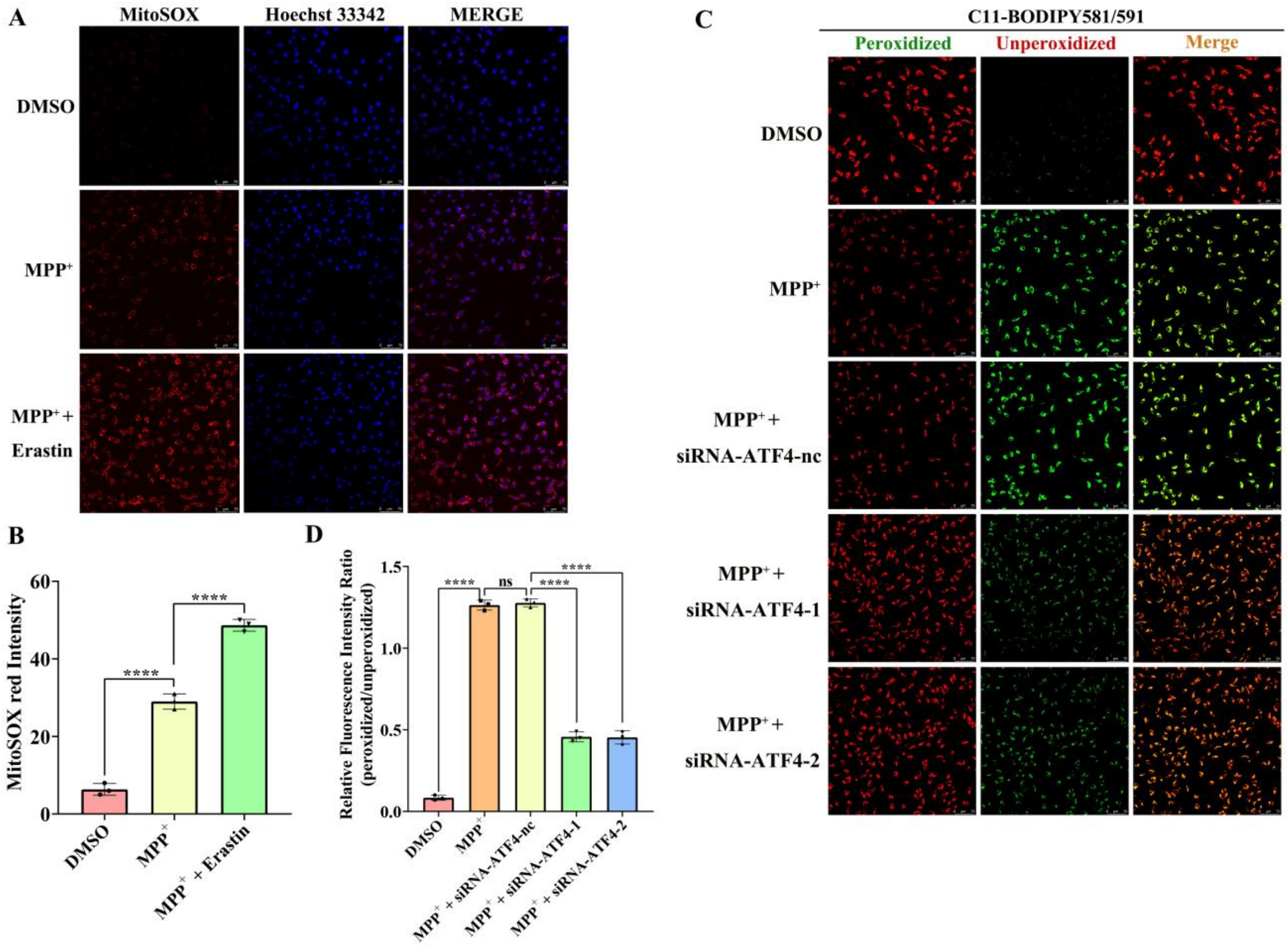

Figure S1. Detection of cellular lipid oxidation levels.

(A,B) MitoSOX assay for mitochondrial oxidative levels and corresponding statistical graphs. Scale bar: 75 μm. (C, D) C11-BODIPY581/591 assay for cell membrane lipid oxidation levels and corresponding statistical graphs. Scale bar: 75 μm. Data are represented as means ± SEM with ordinary one-way ANOVA. ****P <0.0001.

Figure S2. Detection of key gene expression levels.

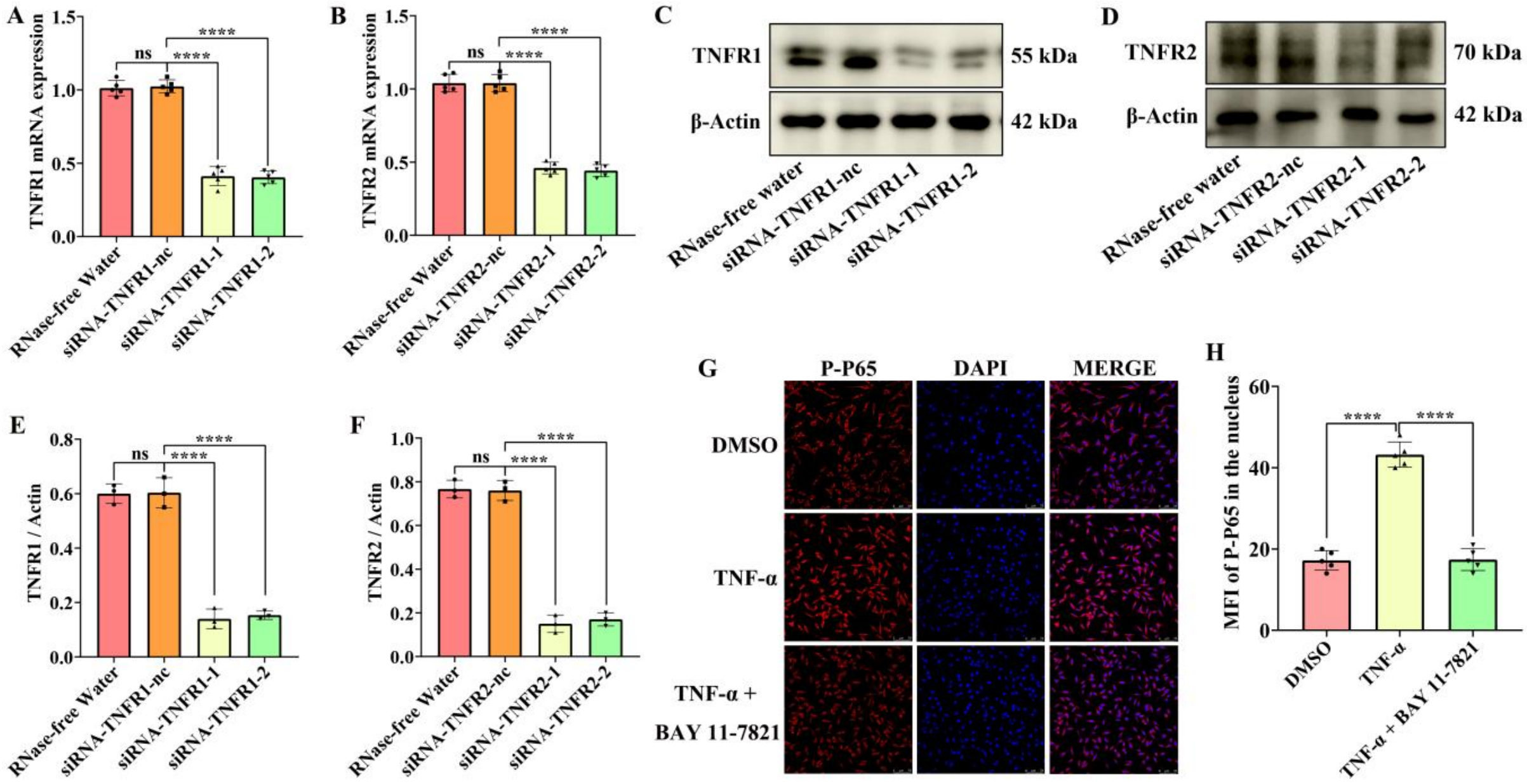

Figure S2. Detection of key gene expression levels.

(A, B) RT-qPCR was used to determine the mRNA expression levels of TNFR1 and TNFR2, respectively. (C, D, E, F) Western blotting was performed to detect the protein expression of TNFR1 and TNFR2 in knockdown cells, with corresponding statistical graphs. (G, H) Immunofluorescence assay was conducted to assess the nuclear translocation of P-P65, along with corresponding statistical graphs. Scale bar: 75 μm. Data are represented as means ± SEM with ordinary one-way ANOVA. ****P <0.0001.

Figure S3. Detection of the ferroptosis signaling pathway in mouse midbrain tissues.

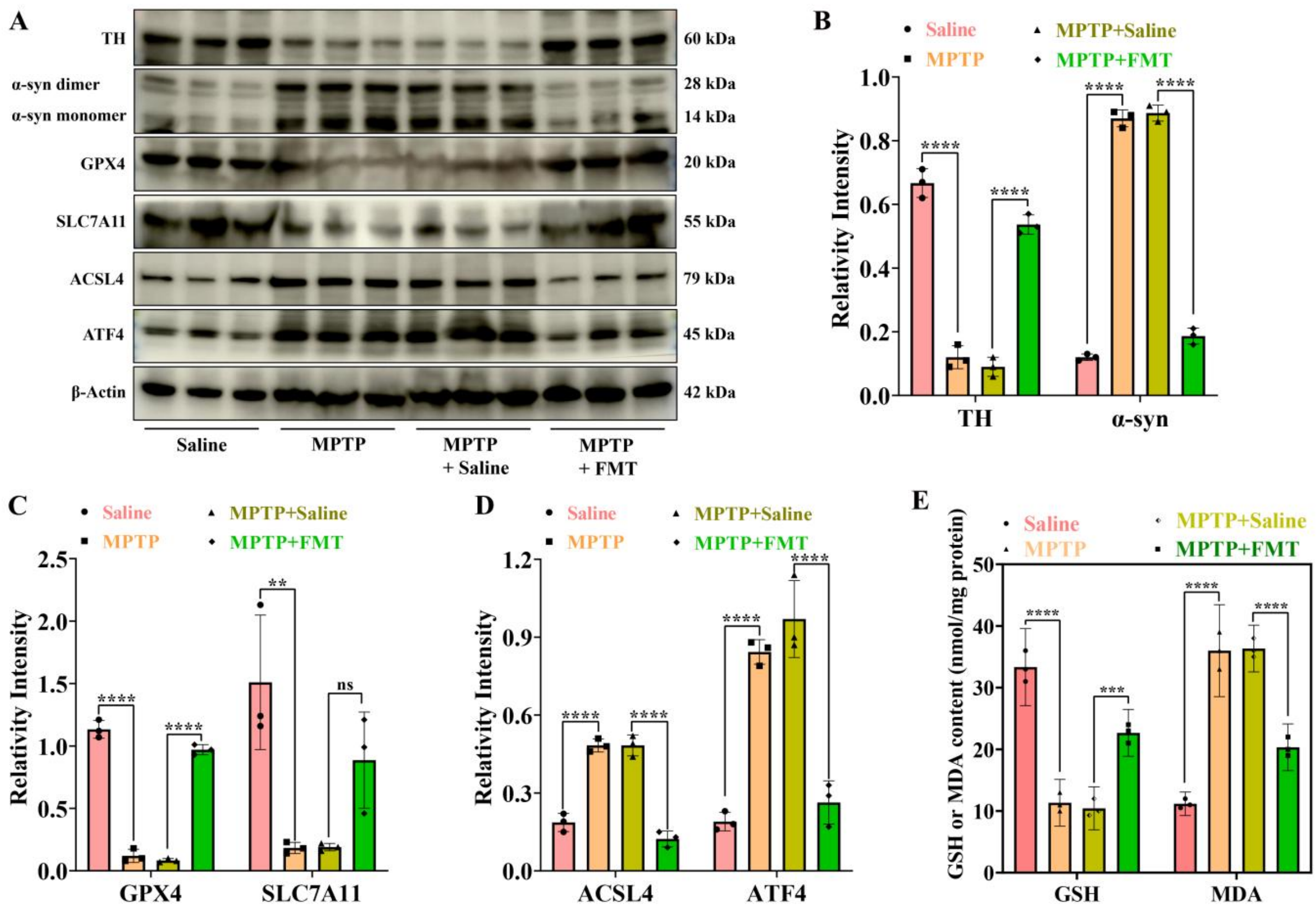

Figure S3. Detection of the ferroptosis signaling pathway in mouse midbrain tissues.

(A, B, C, D) Western blotting was performed to determine the protein expression levels of TH, α-synuclein (A, B), GPX4 (C), SLC7A11 (C), ACSL4 (D), and ATF4 (D), respectively, with corresponding statistical graphs. (E) Determination of GSH and MDA contents in mouse midbrain tissues. Data are represented as means ± SEM with ordinary one-way ANOVA. ****P <0.0001.

Materials and methods

Definition of experimental groups

All animal experiments were performed using 8-week-old male C57BL/6J mice (purchased from Beijing Vital River Laboratory Animal Technology Co., Ltd., China), weighing 22–25 g. All mice were kept on a standard 12:12-h light–dark cycle, with controlled temperature (21–25 °C) and humidity (40–60%), and with freely accessible food and water. The mice were randomly divided into 4 experimental groups (n=10 per group) based on the intervention conditions:

(1) Saline group: Mice were given 120 μl normal saline (0.9% NaCl) twice a week for 5 weeks by intraperitoneal injection.

(2) MPTP group: 1-methyl-4-Phenyl-1,2,3,6-tetrahydropyridine (MPTP) (Biorbyt, #orb363933-1g) was dissolved in normal saline to prepare a working solution. According to the body weight of mice, a chronic PD mice model was given 30 mg/kg MPTP twice a week for 5 weeks by intraperitoneal injection.

(3) MPTP + saline group: A chronic PD mice model was given 30 mg/kg MPTP twice a week for 5 weeks by intraperitoneal injection. Next, PD mice were treated with an antibiotic cocktail (neomycin 0.5g/L, gentamicin 100mg/L, ampicillin 1g/L, vancomycin 0.5g/L, and erythromycin 10mg/L) in drinking water for 7 days to deplete gut microbiota. Afterward, the normal saline (150 μL) was gavaged into antibiotic-pretreated PD mice (PD-saline group) every other day for 2 weeks.

(4) MPTP + FMT group: A chronic PD mice model was given 30 mg/kg MPTP twice a week for 5 weeks by intraperitoneal injection. Next, PD mice were treated with an antibiotic cocktail (neomycin 0.5g/L, gentamicin 100mg/L, ampicillin 1g/L, vancomycin 0.5g/L, and erythromycin 10mg/L) in drinking water for 7 days to deplete gut microbiota. Afterward, fresh feces from healthy C57BL/6J mice were suspended in sterile PBS (100 mg feces/mL), homogenized, and centrifuged at 900 × g for 5 min. The supernatant (150 μL) was gavaged into antibiotic-pretreated PD mice (PD-FMT group) every other day for 2 weeks.

Anesthesia and euthanasia of mice

According to the requirements of Association for Assessment and Accreditation of

Laboratory Animal Care (AAALAC), and with reference to the guidelines outlined in Laboratory Animal Anaesthesia and the AVMA Guidelines on euthanasia, anesthesia was performed prior to euthanasia to minimize the stress response of mice, as such stress could affect the physiological state of tissues and plasma indicators, and the specific procedures are as follows [1]:

Mice were gently placed into a transparent polycarbonate induction chamber, and the chamber was sealed tightly. The isoflurane vaporizer was adjusted to 3%–5% to initiate anesthesia induction, with continuous oxygen supply at 0.8–1.5 L/min. The mice were continuously observed for behavioral changes; induction was considered complete when the mice lost the righting reflex, showed no response to paw pinching, and exhibited a regular, slow respiratory pattern. Prior to euthanasia, the depth of anesthesia was reconfirmed by checking the absence of corneal reflex and pedal withdrawal reflex, ensuring that the mice were in a deep anesthetic state to avoid pain during euthanasia procedures.

The operator firmly grasped the base of the mouse's tail with the dominant hand, ensuring a stable grip without causing tissue damage. With the non-dominant hand, the thumb and index finger were used to firmly press the back of the mouse's neck at the base of the skull, fixing the head in a stationary position. A swift and firm motion was applied: the hand pressing the head was pushed forward and downward, while the hand holding the tail was pulled backward and upward simultaneously, creating a sharp separation between the cervical vertebrae and the skull. A palpable 2–4 mm space between the occipital condyles and the first cervical vertebra indicated successful dislocation of the cervical spine and severance of the spinal cord [2].

Collection of mouse colon tissues and colonic contents

Following euthanasia, the mouse was placed supine on a sterile dissecting tray, and the abdominal skin was disinfected with 75% ethanol. A longitudinal abdominal midline incision (≈2.5 cm) was made from the lower edge of the xiphoid process to the pubic symphysis using sterile surgical scissors, and the skin and subcutaneous fat were gently separated with ophthalmic forceps to expose the abdominal cavity. The colon, located in the posterior abdominal cavity with its proximal end connected to

the cecum and distal end to the anus, was identified; the mesentery attached to the colon was carefully dissected with curved ophthalmic forceps to avoid injury to the colonic wall and associated blood vessels. The entire colon was excised by transecting both ends (proximal to the cecum and distal to the anus) with sterile surgical scissors.

For colonic contents collection, a small incision was made on the excised colon, and the contents were gently squeezed into a sterile, pre-labeled 1.5 mL centrifuge tube, which was immediately snap-frozen in liquid nitrogen and stored at -80 °C for for subsequent 16S rRNA sequencing. For colon tissue processing, the excised colon was gently rinsed with pre-cooled sterile PBS to remove residual feces and blood, blotted dry on sterile gauze. One segment was fixed in 4 °C 4% paraformaldehyde for subsequent histopathological examination.

Collection and isolation of plasma

Plasma was isolated immediately after blood collection to prevent hemolysis and ensure protein integrity. Following euthanasia, a transverse incision was made at the lower edge of the sternum with sterile surgical scissors to open the thoracic cavity and expose the heart . The pericardium was gently torn open with ophthalmic forceps, and approximately 0.8–1.2 mL of blood per mouse was collected from the left ventricle using a sterile 1 mL pipette, which was slowly transferred into a pre-cooled sterile 5 mL anticoagulant centrifuge tube. The blood-containing tube was immediately placed on ice and centrifuged at 3000 rpm for 10 minutes at 4 °C with gentle acceleration and deceleration to avoid damaging cellular components. After centrifugation, the upper pale yellow transparent layer, corresponding to plasma, was carefully aspirated, transferred into pre-labeled sterile 1.5 mL centrifuge tubes, and stored at -80 °C for subsequent biochemical and molecular analyses.

Collection of brain tissues

Brain tissue collection was divided into two groups, with distinct perfusion and sampling protocols implemented according to subsequent experimental purposes. Following blood collection, mice were randomly assigned to two groups for tissue harvesting: For Group 1, transcardiac perfusion was performed with pre-cooled sterile PBS at a constant flow rate to flush out residual blood in the cerebral vasculature,

ensuring the purity of harvested midbrain tissue. After perfusion, the mouse's head was fixed with sterile forceps, the top scalp was incised with surgical scissors to expose the skull, and the skull was carefully fractured along the midline with a dissecting needle to avoid brain tissue damage. The brain was gently removed from the skull using curved ophthalmic forceps and placed in a sterile petri dish containing pre-cooled sterile PBS; the meninges and surface blood vessels were carefully removed under a dissecting microscope. The midbrain, located between the forebrain and hindbrain with a slightly bulging morphology, was identified and excised with a sterile dissecting knife, with surrounding non-midbrain tissues removed to ensure sample purity. The isolated midbrain tissue was immediately placed into a pre-labeled 1.5 mL centrifuge tube, snap-frozen in liquid nitrogen for 5 minutes, and stored at -80 °C for subsequent WB and RNA extraction.

For Group 2, transcardiac perfusion was first performed with pre-cooled sterile PBS to remove intravascular blood, followed by continuous perfusion with pre-cooled 4% paraformaldehyde to achieve adequate tissue fixation. After completion of perfusion, the intact brain was gently removed from the skull using the same skull-exposing and brain-extracting method as Group 1. The entire intact brain was directly placed into centrifuge tube containing pre-cooled 4% paraformaldehyde without further separation, and fixed at 4 °C for 24 hours for subsequent immunohistochemical analysis.

Immunohistochemical detection of TH expression

Immunohistochemistry was performed to detect the protein expression level of tyrosine hydroxylase (TH antibody, 1:500, Santa Cruz Biotechnology, sc-25269) in the substantia nigra and striatum of mice. The brief experimental procedure was as follows: frozen sections of midbrain tissues were first subjected to EDTA-mediated antigen retrieval, followed by incubation with 3% hydrogen peroxide solution for 10 min to block endogenous peroxidase activity. Subsequently, the sections were permeabilized with 0.1% Triton X-100 for 15 min and blocked with 5% bovine serum albumin (BSA) for 1 h at room temperature. The frozen tissue sections were then incubated with the primary TH antibody at 4 °C overnight, and equilibrated to room

temperature the next day. After gentle rinsing with phosphate-buffered saline (PBS), the sections were incubated with horseradish peroxidase (HRP)-conjugated secondary antibody. Finally, 3,3'-diaminobenzidine (DAB) was used for color development, and the stained sections were imaged under a light microscope.

Immunofluorescence (IF) Staining

Cells were seeded on confocal dish, fixed with 4% paraformaldehyde for 15 min, and permeabilized with 0.1% Triton X-100 for 10 min. After blocking with 5% BSA for 1 h, cells were incubated with primary antibody against TNFR1 (1:500, Abcam, ab223352) or P-P65 (1:500, Cell Signaling Technology, #3033) at 4°C overnight, followed by Alexa Fluor 594-conjugated secondary antibody (1:1000, Invitrogen, #A-11012) for 1 h at room temperature. Nuclei were stained with DAPI (1:1000, Beyotime Biotechnology) for 5 min. Fluorescence images were captured using a confocal laser scanning microscope, and fluorescence intensity was quantified using ImageJ software.

Western blotting analysis

Cells were lysed with cold radio-immunoprecipitation assay (RIPA) lysis buffer plus PMSF. The lysed cells were centrifuged at 15000 rpm for 15 minutes at 4°C. The supernatant was collected, and protein concentrations were measured using a bicinchoninic acid (BCA) protein kit. Equal amounts of protein (30 μg) were separated by 10–12% SDS-PAGE and transferred to polyvinylidene difluoride (PVDF) membranes (Millipore). Membranes were blocked with 5% non-fat milk in TBST for 1 h at room temperature, then incubated with primary antibodies at 4°C overnight: ATF4 (1:1000, Abcam, ab216839), TNFR1 (1:500, Abcam, ab223352), TNFR2 (1:500, Abclonal, #A1095SP), P-P65 (1:1000, Cell Signaling Technology, #3033), P-IκBα (1:1000, Cell Signaling Technology, #2859), IκBα (1:1000, Cell Signaling Technology, #4814), P65 (1:1000, Cell Signaling Technology, #8242), GPX4 (1:1000, Abcam, ab125066), SLC7A11 (1:1000, Abcam, ab307601), ACSL4 (1:1000, Abcam, ab155282), α-syn (1:500, Santa Cruz Biotechnology, sc-32280), TH (1:500, Santa Cruz Biotechnology, sc-25269), and β-actin (1:5000, Boster Biological Technology, #BA2305). Membranes were washed with TBST and incubated with HRP-conjugated

secondary antibody (1:5000, Beyotime Biotechnology) for 1 h at room temperature. Signals were visualized using an enhanced chemiluminescence (ECL) detection system, and band intensities were quantified using ImageJ software.

Hematoxylin-Eosin (HE) Staining

Colonic tissues were fixed in 4% paraformaldehyde for 24 h, dehydrated, cleared, and embedded in paraffin. Serial 5 μm-thick sections were prepared and mounted on glass slides. For staining: sections were deparaffinized in xylene (twice, 10 min each), rehydrated through a graded ethanol series (5 min per concentration), and rinsed in distilled water (5 min); stained with hematoxylin for 5 min, differentiated in 1% hydrochloric acid-ethanol for 30 s, and blued under running tap water for 10 min; counterstained with eosin for 2 min, dehydrated, cleared in xylene (twice, 10 min each), and mounted with neutral balsam. Stained sections were observed under a light microscope. Intestinal mucosal integrity and goblet cell density were independently evaluated by two blinded pathologists.

Enzyme-Linked Immunosorbent Assay (ELISA)

Tumor necrosis factor-α (TNF-α) levels in colonic contents, plasma, and midbrain tissues were measured using a mouse TNF-α ELISA kit (Finetest, Wuhan Fine Biotech Co., Ltd.) in strict accordance with the manufacturer's instructions. The concentration of activating transcription factor 4 (ATF4) protein in cells was determined with a mouse ATF4 ELISA kit (Finetest, Wuhan Fine Biotech Co., Ltd.). The absorbance values of all samples were read at a wavelength of 450 nm using a microplate reader.

Reverse transcription-quantitative PCR (RT-qPCR)

For RT-qPCR, total RNA was extracted with Trizol (Invitrogen) and then reverse-transcribed using the PrimeScript-RT reagent kit (TaKaRa, for mRNA). RT-qPCR was performed using the TB Green Premix Ex Taq II (Tli RNaseH Plus) (TaKaRa). The reaction conditions were: 95°C for 30 s, followed by 40 cycles of 95°C for 5 s and 60°C for 30 s. Actin (for mRNA expression) was used as a control for normalization, and gene expression was counted using the $2^{-\Delta\Delta CT}$ method. The primer sequences are listed in Supplementary table 1.

RNA interference

siRNAs targeting ATF4, TNFR1, TNFR2, and negative control siRNA (siRNA-nc) were synthesized by GenePharma (Shanghai, China). Detailed sequences of siRNAs are provided in Supplementary table 2. Cells were transfected with siRNAs (50 nM) using Lipofectamine 2000 (Invitrogen) according to the manufacturer's protocol.

ROS levels assay

Mitochondrial reactive oxygen species (mitoROS) production in MN9D cells was quantified using MitoSOX™ Red (Thermo Fisher Scientific, Waltham, MA, USA). Briefly, MN9D cells were incubated in serum-free medium supplemented with 5 μM MitoSOX Red fluorescent probe at 37°C for 15 min, and subsequently stained with hoechst 33342 for 5 min at 37 °C in the dark. Fluorescence intensity was subsequently measured using a microscope, with excitation and emission wavelengths set at 396 nm and 610 nm, respectively.

Labeling cells with C11-BODIPY581/591

Fluorescent indicators (C11-BODIPY) were used to measure lipid peroxidation. The fluorescent data was collected using a confocal microscope. Cells were stained with 5 μM C11-BODIPY581/591 fluorescent probe (Invitrogen) at 37°C for 30 min. Unperoxidized lipids emit red fluorescence (excitation/emission: 581/591 nm), while peroxidized lipids emit green fluorescence (excitation/emission: 488/520 nm). The green/red fluorescence ratio was quantified using ImageJ software.

Transmission Electron Microscopy (TEM)

Cells were rinsed three times with 0.1 M phosphate buffer (PB, pH 7.4) for 15 minutes each time, followed by fixation with 1% osmium tetroxide prepared in 0.1 M PB (pH 7.4) at room temperature for 2 hours in the dark. The samples were dehydrated through a graded ethanol series and embedded in embedding medium. Ultrathin sections (70 nm) were cut from the resin blocks using an ultramicrotome, and collected onto 200-mesh copper grids coated with Formvar film. The grids were stained with 2% uranyl acetate saturated alcohol solution in the dark for 10 minutes, rinsed three times with ultrapure water, then stained with lead citrate solution for 10 minutes while avoiding carbon dioxide exposure. After three additional rinses with

ultrapure water, excess liquid was gently blotted with filter paper. The grids with sections were placed in a grid box and dried overnight at room temperature. Observations were performed using a transmission electron microscope (HT7800, HITACHI). Mitochondrial morphology (volume, membrane density, and cristae structure) was analyzed by two independent researchers.

Cellular viability assay

Cell counting kit-8 (CCK-8) was used to measure MN9D cellular viability. MN9D cells suspension (≈1×10^4 cells) was added to 96-well plates. After treatment, 10 μL of CCK-8 reagent (Beyotime Biotechnology) was added to each well, and cells were incubated at 37°C for 2 h. Absorbance was read at 450 nm using a microplate reader. Cell viability was calculated as the percentage of absorbance in treated groups relative to the control group.

Glutathione (GSH) and Malondialdehyde (MDA) Detection

Intracellular GSH and MDA levels were measured using the GSH Assay Kit and MDA Assay Kit (Beyotime Biotechnology) according to the manufacturer's instructions. Briefly, cells or midbrain tissues were homogenized in lysis buffer, centrifuged at 10,000g at 4℃ for 10 minutes, and supernatants were collected. GSH content was detected by measuring absorbance at 412 nm, and MDA content was detected at 532 nm using a microplate reader.

Acquisition of GWAS datasets

The GWAS (Genome-wide association study) data on intestinal microbiota was obtained through the MiBioGen consortium database, which incorporates 16S fecal microbiome data from 18,340 individuals and a total of 211 types of GWAS data for gut microbial [3]. For the outcome dataset, the GWAS data for Parkinson's Disease was obtained from the IEU Open GWAS database (https://gwas.mrcieu.ac.uk/) [4]. The GWAS ID is ieu-b-7, which contains 17,891,936 variant loci from 33,674 cases and 449,056 controls.

Filter the instrumental variables (IVs) related to exposure and outcome

We have filtered the relevant IVs based on the following criteria: First, we selected SNPs that are significantly associated with the gut microbiome ($P < 1\times10^{-5}$); then, we

eliminated SNPs with linkage disequilibrium (LD) within the IVs by setting r2 <0.001 and distance >10,000 kb; finally, we removed the influence of pleiotropic effects, removing palindromic SNPs and SNPs with F-statistic parameters less than 10 [5].

Mendelian Randomization (MR) analysis

After determining the instrumental variables for the exposure factors, we further evaluated the causal effect of the gut microbiome on the etiology of PD through two-sample MR. Initially, MR-PRESSO was utilized to identify and adjust for outlier variables with pleiotropic biases. The two-sample MR analysis incorporated five distinct methods, which included inverse variance weighting (IVW) with a random effects model, weighted median, MR-Egger, simple mode and weighted mode. Each method assumed different things about the validity of IVs, yet the IVW approach is typically regarded as the most robust. MR findings were conveyed as Odds Ratios (ORs), along with their respective 95% confidence intervals (CI). Outcomes were deemed statistically significant when the IVW's P-value was below 0.05 and the orientation of IVW was in line with that of MR-Egger, represented visually through forest plots [6]. The analysis results revealed that there are two types of gut microbiota associated with PD, and the detailed information on the 22 SNPs concerning the two types of gut microbiota (Supplementary table 3). The data sources for our MR analysis are as follows: MR exposure: Gut microbiota GWAS data derived from the MiBioGen consortium, based on gut microbiome profiles from fecal samples rather than brain tissue. MR outcome: GWAS data for PD risk obtained from public databases, specifically the IEU Open GWAS database.

Sensitivity Analysis

We assessed the heterogeneity of each SNP through Cochran's Q test, with p-values determined by Cochran's Q test less than 0.05 being considered indicative of heterogeneity. The possibility of horizontal pleiotropy was evaluated using MR-PRESSO and MR-Egger regression, and a leave-one-out analysis was conducted to assess the potential impact of each SNP on the estimated value. All analyses were performed using R (v4.2.0) statistical software. MR analysis was carried out using the R-based software package "TwoSampleMR" [7, 8].

Differentially expressed gene (DEG) analysis

The GSE20292 dataset was pretreated with background correction, standardization and expression value calculation using R software's "limma package". |Values of log2(fold change)|≥0.5 and *p-values* <0.05 were used to define differentially expressed genes (DEGs) (Supplementary table 4). We plotted a total of 720 DEGs in the form of a volcano map using the "ggplot2 package".

Functional enrichment analysis

For functional enrichment analysis, we utilized the "clusterProfiler" package in R software to analyze the Kyoto Encyclopedia of Genes and Genomes (KEGG) pathways and identify representative biological processes, molecular functions, or cellular components associated with our differentially expressed genes. The KEGG pathway analysis of 19 DE-FRGs was performed using the R software "cluster profiler package". The screening threshold was a *P value* <0.05.

Screening method for TF-related FRGs

We began with an extensive literature review to identify transcription factors that have been reported to be associated with ferroptosis, along with their potential target genes. This step helped us construct an initial candidate list of TF-FRGs pairs based on functional associations and experimental validations reported in previous studies. Subsequently, we utilized bioinformatics tools and the GeneCards database to search for 313 ferroptosis-related genes (FRGs)(Supplementary table 5). These tools enabled us to identify genes that are potentially related to the candidates of interest.

Validation of activating transcription factor 4 (ATF4)

To further select the most significant FRGs, we used the GSE7621 and GSE20163 cohorts to validate FRGs correlation signatures. Batch effects were removed using the "sva package". We developed an expression matrix, using the "limma package" to perform background correction, standardization and expression value calculation (Supplementary table 6).

Bulk RNA Sequencing (RNA-seq) and analysis methods

Total RNA was extracted from the mouse midbrain using TRIzol Reagent, followed by reverse transcription and amplification of RNA in accordance with the

manufacturer's instructions. Sequencing libraries were constructed on the Illumina Novaseq 6000 platform (Genedenovo, Guangzhou, China), and RNA-seq analysis was performed by Genedenovo using standard and consistent procedures with a sequencing depth of 10G data volume. First, raw data were preprocessed to remove reads containing sequencing adapters and low-quality reads. Second, transcriptional reads were mapped to the mouse genome (mm10, Ensembl release 95) using HISAT2 software. Differentially expressed genes (DEGs) were identified using the edgeR package (http://www.bioconductor.org/packages/release/bioc/html/edgeR.html), and mRNAs with a fold change ≥ 2 and a false discovery rate (FDR) < 0.05 were considered significant DEGs (Supplementary table 7). For functional enrichment analysis, Kyoto Encyclopedia of Genes and Genomes (KEGG) pathway analysis of the identified mRNAs was conducted using the R package clusterProfiler; KEGG is a major public pathway-related database (http://www.kegg.jp/kegg/), and pathway enrichment analysis was used to identify significantly enriched metabolic pathways or signal transduction pathways.

16S rRNA sequencing analysis of gut microbiota

Chronic PD mice were successfully constructed and fecal samples were collected. Colonic contents were collected in separate sterile EP tubes, quickly frozen in liquid nitrogen, and then stored at -80°C. The V3-V4 region of the 16S rRNA gene was amplified. The changes in the composition of intestinal bacteria were measured by sequencing the bacterial 16S rRNA sequencing (Supplementary table 8). For 16S rRNA gene sequencing, the sequencing depth was 50,000 reads with a data size of 12.5 MB. Alpha Diversity: The number of operational taxonomic units (OTUs) and ACE index were calculated using QIIME-2 to assess within-sample diversity. Beta Diversity: Bray-Curtis distance matrix was generated, and principal co-ordinates analysis (PCoA) was performed to visualize between-group differences. Differential taxa identification: Linear discriminant analysis effect size (LEfSe) was used to identify taxa with significant differences in relative abundance. Functional prediction: Phylogenetic Investigation of Communities by Reconstruction of Unobserved States (PICRUSt) was employed to predict microbial functional pathways based on OTU

profiles, and KEGG pathway enrichment analysis was conducted [9, 10]. The sequencing and bioinformatics analysis of intestinal bacteria were conducted by Shanghai Applied Protein Technology Co., Ltd. (Shanghai, China).

Statistical analysis

All statistical analyses were performed using GraphPad Prism 9.0 software and R 4.2.0. Data are presented as mean±standard error of the mean (SEM). The normality of the data was first evaluated using the Shapiro-Wilk test. For data that satisfied the normality assumption, comparisons between two groups were conducted using the two-tailed unpaired student's t-test, while multiple group comparisons were analyzed by one-way or two-way repeated measurement analysis of variance (ANOVA) followed by Tukey's post-hoc test. For data that did not meet the normality assumption, non-parametric tests were applied: the Mann–Whitney U test was used for comparisons between two groups, and the Kruskal–Wallis test followed by Dunn's post-hoc test was used for multiple group comparisons. Linear correlation analysis (Pearson's test) was performed to assess the relationship between ATF4 expression and ferroptosis indicators. $P<0.05$ was considered statistically significant. *$P<0.05$, **$P<0.01$, ***$P<0.001$, ****$P<0.0001$. Each experiment was repeated independently at least three times (n≥3).

Reference

[1] Newcomer CE. The evolution and adoption of standards used by AAALAC. J Am Assoc Lab Anim Sci. 2012 May;51(3):293-7.

[2] Carbone L, Carbone ET, Yi EM, Bauer DB, Lindstrom KA, Parker JM, Austin JA, Seo Y, Gandhi AD, Wilkerson JD. Assessing cervical dislocation as a humane euthanasia method in mice. J Am Assoc Lab Anim Sci. 2012 May;51(3):352-6.

[3] Kurilshikov A, Medina-Gomez C, Bacigalupe R, Radjabzadeh D, Wang J, Demirkan A, et al. Large-scale association analyses identify host factors influencing human gut microbiome composition. Nat Genet. 2021 Feb;53(2):156-165.

[4] Hemani G, Zheng J, Elsworth B, Wade KH, Baird D, Haberland V, Laurin C, Burgess S, Bowden J, Langdon R, Tan VY, Yarmolinsky J, Shihab HA, Timpson NJ, Evans DM, Relton C, Martin RM, Davey Smith G, Gaunt TR, Haycock PC, The MR-Base Collaboration. eLife 2018;7:e34408.

[5] A. Kurilshikov, C. Medina-Gomez, R. Bacigalupe, D. Radjabzadeh, J. Wang, A. et al. Zhernakova, Large-scale association analyses identify host factors influencing human gut microbiome composition, Nat Genet, 53 (2021) 156-165.

[6] D. Ji, W.Z. Chen, L. Zhang, Z.H. Zhang, L.J. Chen, Gut microbiota, circulating cytokines and

dementia: a Mendelian randomization study, Journal of neuroinflammation, 21 (2024) 2.
[7] Z. Song, W. Li, Y. Han, Y. Xu, H. Ding, Y. Wang, Association of immune cell traits with Parkinson's disease: a Mendelian randomization study, Front Aging Neurosci, 16 (2024) 1340110.
[8] M. Yang, X. Wan, H. Zheng, K. Xu, J. Xie, H. Yu, J. Wang, P. Xu, No Evidence of a Genetic Causal Relationship between Ankylosing Spondylitis and Gut Microbiota: A Two-Sample Mendelian Randomization Study, Nutrients, 15 (2023).
[9] Langille MG, Zaneveld J, Caporaso JG, McDonald D, Knights D, Reyes JA, Clemente JC, Burkepile DE, Vega Thurber RL, Knight R, Beiko RG, Huttenhower C. Predictive functional profiling of microbial communities using 16S rRNA marker gene sequences. Nat Biotechnol. 2013 Sep;31(9):814-21.
[10] Wu X, Tian X, Cao G, Wang Z, Wu X, Gu Y, Yan T. Distinct profiles of bile acid metabolism caused by gut microbiota in kidney transplantation recipients revealed by 16S rRNA gene sequencing. Arch Physiol Biochem. 2024 Oct;130(5):581-590.

Supplementary tables:

Supplementary table 1. List of gene primers

**Gene name**	**Forward primer**	**Reverse primer**
ATF4	5'-TCTGCCTTCTCCAGGTGGTTCC-3'	5'-GCTGCTGTCTTGTTTTGCTCCATC-3'
TNFR1	5′-TCCGCTTGCAAATGTCACA-3′	5′-GGCAACAGCACCGCAGTAC-3′
TNFR2	5'-TTCTGGAGTTGGCTGCGTGT-3'	5'-ACTCTCCTATCCTGCCTGCT-3'
Actin	5'-GTGACGTTGACATCCGTAAAGA-3'	5'-GCCGGACTCATCGTACTCC-3'

Supplementary table 2. List of siRNA sequences

Gene symbol	Sense strand	Antisense strand
siRNA-ATF4-1	5’-UUGGCUCGCUGUGGACAGUUUA-3’	5’-UAAACUGUCCACAGCGAGCCAA-3’
siRNA-ATF4-2	5’-GUUAGGUAUGGAACAUCUGGA-3’	5’-UCCAGAUGUUCCAUACCUAAC-3’
siRNA-TNFR1-1	5′-GCCUCGUGCUUUCCAAGAUTT-3′	5’-AUCUUGGAAAGCACGAGGCTT-3’
siRNA-TNFR1-2	5′-CCCGAAGUCUACUCCAUCATT-3′	5’-UGAUGGAGUAGACUUCGGGTT-3’
siRNA-TNFR2-1	5'-CCUGGCUAUUCCCGGAAAUTT-3'	5’-AUUUCCGGGAAUAGCCAGGTT-3’
siRNA-TNFR2-2	5'-CUGGCUGGUUUGAUCAGAUTT-3'	5’-AUCUGAUCAAACCAGCCAGTT-3’
scrambled siRNA	5’-CCCACUCAUACCUGAUCUCUAAU-3’	5’-AUUAGAGAUCAGGUAUGAGUGGG-3’

Supplementary table 3. Detailed information on the 22 SNPs of gut microbiota

Microbiota name	SNP	effect_allele.exposure	other_allele.exposure	effect_allele.outcome	other_allele.outcome	beta.exposure	beta.outcome
Candidatus Soleaferrea	rs10090365	A	G	A	G	-0.083441348	-0.0213
Candidatus Soleaferrea	rs10108780	A	G	A	G	-0.09283404	2.00E-04
Candidatus Soleaferrea	rs10809135	T	C	T	C	0.08348579	0.0172
Candidatus Soleaferrea	rs11153159	G	C	G	C	-0.128050003	-0.059
Candidatus Soleaferrea	rs2193878	T	A	T	A	0.228255107	0.0051
Candidatus Soleaferrea	rs36155147	C	T	C	T	0.104966126	0.1002
Candidatus Soleaferrea	rs4294381	T	C	T	C	0.112196526	0.0316
Candidatus Soleaferrea	rs4678258	T	C	T	C	0.098613616	0.0066
Candidatus Soleaferrea	rs6489992	A	G	A	G	-0.084043749	-0.0013
Candidatus Soleaferrea	rs6494306	A	G	A	G	-0.09693036	-0.0249
Candidatus Soleaferrea	rs7400877	T	C	T	C	-0.095108487	0.0016
Candidatus Soleaferrea	rs830149	C	G	C	G	0.184646616	0.0641
Candidatus Soleaferrea	rs9973954	A	G	A	G	0.089207514	0.0102

Clostridium sensu stricto 1	rs112644403	G	A	G	A	-0.139061503	-0.0278
Clostridium sensu stricto 1	rs115807074	A	G	A	G	-0.227359786	-0.0797
Clostridium sensu stricto 1	rs11586026	A	T	A	T	0.110966819	-4.00E-04
Clostridium sensu stricto 1	rs116847295	C	T	C	T	0.110021418	0.0088
Clostridium sensu stricto 1	rs12341505	G	A	G	A	0.081071775	0.0313
Clostridium sensu stricto 1	rs124903 37	C	G	C	G	-0.061687867	-0.0392
Clostridium sensu stricto 1	rs2795528	G	A	G	A	-0.184314695	-0.1016
Clostridium sensu stricto 1	rs2817172	C	T	C	T	0.058138957	-0.0087
Clostridium sensu stricto 1	rs550843	T	C	T	C	-0.078324645	-0.0266

Supplementary table 4. List of differentially expressed genes

Gene name	AveExpr	t	P.Value	adj.P.Val
ACTN4	8.731908738	6.119340111	9.38E-07	0.009364901
PACS2	9.052332389	5.97803104	1.40E-06	0.009364901
SLC6A10P	7.71398215	5.724897797	2.86E-06	0.012747528
COBL	10.40970891	5.484152358	5.65E-06	0.012747528
PSMC1	11.04860297	-5.481307173	5.70E-06	0.012747528
CHN1	11.43373095	-5.323409395	8.93E-06	0.014978579
LOC100272216	9.002347455	5.091782512	1.73E-05	0.024934174
ZBTB16	9.184886864	5.06287865	1.87E-05	0.024934174
APLP1	10.24250029	5.016309452	2.14E-05	0.024934174
SUN2	10.06853888	5.001755356	2.23E-05	0.024934174
B3GAT1	10.11733671	4.930459435	2.73E-05	0.025664706
CDK18	7.518189299	4.910818529	2.89E-05	0.025664706
BIN1	9.656825884	4.895992812	3.01E-05	0.025664706
GABARAPL1	8.559045106	-4.890379448	3.06E-05	0.025664706
PID1	8.716932608	-4.849738293	3.44E-05	0.025844828
NDUFA6	9.690903702	-4.846444479	3.47E-05	0.025844828
ATP6V1D	9.032374731	-4.802491381	3.93E-05	0.02668761
CDC42	8.470132457	-4.772617128	4.28E-05	0.026883028
C14orf2	10.82512152	-4.750066542	4.56E-05	0.026883028
TYK2	8.643193639	4.746170107	4.61E-05	0.026883028
KIAA0319	6.906584239	-4.699563899	5.26E-05	0.02940061
HAPLN2	8.355175512	4.654698532	5.97E-05	0.032047888
CAMLG	9.77484661	-4.639585987	6.23E-05	0.032161701
FAM50A	9.161635484	4.546685188	8.10E-05	0.034899699
PLXND1	8.740297608	4.511378729	8.95E-05	0.034899699
SLCO3A1	9.217117974	4.501333217	9.21E-05	0.034899699
DCP1A	8.052952121	4.498285427	9.29E-05	0.034899699
ABCA2	7.143943016	4.494569007	9.39E-05	0.034899699
SV2B	9.580458755	-4.483339659	9.69E-05	0.034899699
PTDSS1	10.07142485	-4.47878014	9.81E-05	0.034899699

		8		
GNA11	8.837506845	4.466240379	0.000101676	0.034899699
LHPP	9.167465594	4.464660282	0.00010213	0.034899699
CXorf40A	8.753793038	-4.441668882	0.000108961	0.034899699
NDUFA8	10.26366091	-4.41603588	0.00011711	0.034899699
NFASC	9.025822525	4.415956129	0.000117137	0.034899699
BCL2L2	10.68353842	4.41108868	0.000118751	0.034899699
PHC2	9.758441157	4.409879138	0.000119156	0.034899699
GGNBP2	9.139539972	-4.408961128	0.000119464	0.034899699
LRRC49	8.395617625	-4.401492484	0.000121999	0.034899699
KCND3	7.054377889	-4.393554465	0.000124752	0.034899699
DDR1	10.61730989	4.388326212	0.000126599	0.034899699
ARL1	7.59143497	-4.382922749	0.000128536	0.034899699
NACAD	9.552166467	4.356769097	0.000138332	0.034899699
PRR14	8.69866148	4.353280865	0.000139693	0.034899699
SV2C	9.050379699	-4.352367019	0.000140052	0.034899699
CHCHD2	10.9693881	-4.333371383	0.000147719	0.034899699
PAQR4	9.383019791	4.331412191	0.000148533	0.034899699
LARP6	10.02484584	4.326870175	0.000150437	0.034899699
SASH1	10.77705105	4.321238972	0.000152831	0.034899699
VASH1	8.483952516	4.308432569	0.000158417	0.034899699
MOAP1	10.13539932	-4.306690041	0.000159193	0.034899699
SETD5	8.399680514	4.295951795	0.000164055	0.034899699
GBE1	8.963763904	-4.294791009	0.000164589	0.034899699
NDUFA1	11.3973988	-4.290724826	0.000166474	0.034899699
DENND4B	9.335964786	4.27795587	0.000172533	0.035613492
SYT1	8.154770441	-4.266650536	0.000178079	0.036201301
HMGB3	8.496125899	-4.259582621	0.000181635	0.036373104
DCLK1	7.886954904	-4.253834523	0.000184579	0.036419003
YPEL5	10.00262429	-4.222664804	0.000201378	0.038054842

RNF220	9.830628759	4.206954119	0.000210408	0.039208991
SNRPG	9.149168588	-4.128361284	0.000261917	0.042741658
ATP6V1A	8.348237568	-4.127213875	0.000262754	0.042741658
SERPINB6	9.547850398	4.125018663	0.000264363	0.042741658
RAP1GAP2	8.27855133	-4.123101703	0.000265776	0.042741658
TRIM28	7.82959552	4.109695342	0.000275867	0.042741658
NME5	7.898482582	-4.106786738	0.000278106	0.042741658
GABARAPL1	12.37704395	-4.099266396	0.000283977	0.042741658
COL18A1	7.833467787	4.092701333	0.000289202	0.042741658
DNAJB2	9.810440923	4.089905533	0.000291455	0.042741658
SLC18A2	7.552434769	-4.075969239	0.000302948	0.042741658
MYH10	7.971246266	-4.073391029	0.000305123	0.042741658
SRI	8.809100023	-4.072543457	0.000305841	0.042741658
MIR4745	8.056117308	4.070802911	0.000307321	0.042741658
PSMB7	10.16670348	-4.069685154	0.000308275	0.042741658
LRRN3	8.585956464	-4.069162187	0.000308723	0.042741658
ATPIF1	9.903433867	-4.068886795	0.000308959	0.042741658
LOC399491	10.59748076	4.057907446	0.000318508	0.042741658
NDUFB3	9.338298671	-4.05352473	0.0003224	0.042741658
ATF4	10.58114373	4.053402239	0.00032251	0.042741658
CLIP2	8.333557462	4.047675106	0.000327668	0.042741658
STAB1	8.219896692	4.043829635	0.000331177	0.042741658
RASL12	8.84885189	4.039174073	0.000335474	0.042741658
WASF2	8.692010816	4.037261016	0.000337256	0.042741658
RBX1	9.446026461	-4.035248303	0.000339141	0.042741658
PSMD14	10.3366985	-4.027761076	0.000346242	0.043014215
EHBP1	8.199225699	-4.020496978	0.000353272	0.043323681
CDK14	7.699375008	-4.012022507	0.00036165	0.043323681
CRYAB	11.99155162	4.006596737	0.000367115	0.043589262

SH3BP4	9.556534074	4.002873302	0.000370913	0.043653859
NDUFS1	7.627916515	-3.992681406	0.000381506	0.044414304
NSUN5P1	8.829386914	3.990327251	0.000383995	0.044414304
HNRNPA0	8.052892587	-3.984280768	0.00039046	0.044776088
ZDHHC14	9.551349278	3.972696492	0.000403146	0.045178045
MDH1	11.29386638	-3.969418363	0.000406808	0.045178045
KCNJ6	8.379866759	-3.968861108	0.000407434	0.045178045
SMYD3	8.15878301	-3.964011316	0.000412921	0.045411124
MICAL3	7.213883458	3.956626592	0.000421414	0.045653051
PIEZO1	7.779790277	3.954467002	0.00042393	0.045653051
RPS6KA2	9.796380448	3.947620395	0.000432005	0.046001648
ARL3	7.812520032	-3.935230377	0.000447001	0.04685479
RABGAP1L	7.623738612	-3.914724912	0.000472947	0.049190107
MAN2A1	9.650135954	3.911452268	0.000477222	0.049247405
CSNK1E	8.323798961	3.90870568	0.000480838	0.049247405
SLC6A3	9.280764369	-3.902328456	0.00048934	0.049738479
ZCCHC24	11.05443989	3.889456153	0.000506951	0.051141102
GTF2H5	8.073022545	-3.882911531	0.000516141	0.051679622
FAM49A	7.201463945	-3.876654276	0.00052508	0.051801469
RALYL	9.210132228	-3.863252111	0.000544738	0.053165548
LTBP3	7.224286291	3.861852109	0.000546832	0.053165548
TENC1	8.322177378	3.849267853	0.000566015	0.054322493
RGS4	7.666052805	-3.845774113	0.000571456	0.054322493
NPIPA1	10.92985889	3.843667408	0.000574762	0.054322493
TMEM255A	6.830021752	-3.84356276	0.000574927	0.054322493
MAP3K11	8.909889181	3.837026979	0.000585305	0.054916314
FLCN	7.500955882	3.834304653	0.000589681	0.054942714
ACADVL	10.27902027	3.829265082	0.000597867	0.055028575
ACBD3	7.613112905	-3.823099818	0.000608033	0.055028575
ARPP21	6.53935918	-3.820118293	0.000613009	0.055028575

ZRSR2	9.636384172	3.819808881	0.000613528	0.055028575
CRTC3	10.17894697	3.819680515	0.000613743	0.055028575
GHITM	10.31449032	-3.816362812	0.000619334	0.055028575
YWHAZ	8.559170215	-3.811838885	0.000627037	0.055028575
PHYH	8.249563002	-3.811559953	0.000627515	0.055028575
ATP5G3	10.18944644	-3.803578131	0.000641346	0.05538353
SNX10	7.888108273	-3.802191157	0.000643779	0.05538353
TCIRG1	7.120719527	3.800616403	0.000646553	0.05538353
VSNL1	8.417239124	-3.795086766	0.000656386	0.055497542
DHCR24	9.669720562	3.787794367	0.000669576	0.055497542
ATP5A1	12.01183224	-3.785896054	0.000673052	0.055497542
CADPS2	8.945505019	-3.785256721	0.000674227	0.055497542
ZNF226	7.940909108	-3.779639313	0.000684633	0.056010517
GTPBP6	8.921633412	3.766986343	0.000708651	0.056897479
BEX1	11.66251134	-3.762850089	0.000716678	0.056897479
PEX26	7.345607941	-3.760562068	0.000721157	0.056916264
LOC101930324	9.873293822	-3.757692345	0.000726813	0.057027196
MID1IP1	9.438382326	3.75374592	0.000734662	0.057307881
UBL5	8.712747998	-3.749623024	0.000742949	0.057434631
FAM216A	8.362527866	-3.748684857	0.000744848	0.057434631
PGAM1	11.50445091	-3.742262304	0.000757973	0.058112695
SFXN1	7.13618924	-3.730372167	0.000782868	0.059607033
FGR	7.558113896	3.728738962	0.000786349	0.059607033
CAPZA1	9.798403854	-3.726496592	0.000791153	0.059634277
NXF1	8.311470419	3.716524294	0.000812866	0.060286395
GABBR2	8.380871722	-3.714304537	0.000817778	0.060286395

AMPH	8.291532455	-3.710173241	0.000826996	0.060632799
IPO13	9.350705207	3.700840112	0.000848194	0.061514713
CPSF7	9.114366395	3.692484687	0.00086762	0.062250556
PTBP1	9.118321749	3.683511456	0.000888963	0.06291579
SLC38A10	8.51019603	3.682917336	0.000890394	0.06291579
LGALS3BP	9.23681194	3.682683088	0.000890959	0.06291579
HEG1	8.629452324	3.667318204	0.000928779	0.065243099
LCMT1	8.833114238	-3.661340316	0.000943911	0.065650538
CXorf40B	8.695270089	-3.661162299	0.000944366	0.065650538
CCNH	8.45814625	-3.659177869	0.000949444	0.065663354
NSUN5P1	9.523398782	3.653816002	0.000963299	0.065886827
NDUFC1	10.36685144	-3.645492191	0.000985197	0.065886827
ABL1	8.627294263	3.645049475	0.000986375	0.065886827
AUP1	8.029752495	3.640244169	0.000999251	0.065886827
GNAL	6.599957191	-3.640027438	0.000999835	0.065886827
ZHX2	9.174724917	3.639781714	0.001000499	0.065886827
DYNLT3	9.68169245	-3.639306536	0.001001782	0.065886827
IMPA1	9.462501986	-3.636542334	0.001009281	0.066056223
REEP1	8.882593081	-3.633281415	0.001018198	0.066316306
TBC1D30	7.109883527	-3.630262368	0.001026521	0.066535425
IDI1	9.579731778	-3.626013854	0.001038346	0.066575994
MMD	8.696351025	-3.625130962	0.00104082	0.066575994
COPS4	8.965828951	-3.624659731	0.001042143	0.066575994
ANXA13	7.411258028	3.613945962	0.00107266	0.067304339
MFSD1	9.005132285	-3.61230335	0.001077415	0.067304339
SET	9.451365671	3.608987575	0.001087075	0.067336099
LOC101930075	8.8039796	3.606429717	0.001094585	0.067336099
ABHD17A	9.694467695	3.603237331	0.001104028	0.067336099
MAGED1	10.89668009	-3.60039429	0.001112505	0.067336099
ATP5B	10.99141185	-3.59999815	0.001113691	0.067336099
PRKCSH	8.761138143	3.598185715	0.001119133	0.067336099

IGF2BP2	7.082577988	3.598172291	0.001119173	0.067336099
NAA60	9.21704819	3.593166506	0.001134339	0.067943879
LDHB	12.72163717	-3.587199924	0.001152676	0.068735364
C3orf14	8.415996724	-3.585006219	0.00115949	0.068835746
CD200	7.394490532	-3.582724967	0.001166617	0.068873754
PLEKHB1	11.82499181	3.58152067	0.001170397	0.068873754
SOX10	8.56128111	3.573103582	0.001197147	0.069634922
PPCS	6.849989481	-3.569255804	0.001209572	0.069951838
NDUFAB1	10.55276924	-3.558755363	0.001244118	0.07164091
FGF13	7.942771601	-3.555838359	0.001253883	0.071894669
ATP5C1	9.659061131	-3.544678297	0.001291934	0.072224476
PTS	9.336620008	-3.54020926	0.001307482	0.072533532
CORO2B	9.43348305	3.539368506	0.001310428	0.072533532
VRK1	6.874484906	-3.538442156	0.00131368	0.072533532
CAP2	7.764907159	-3.536126241	0.001321846	0.072685289
FAM136A	8.088254688	-3.532424143	0.001335002	0.073109065
DENR	9.870118327	-3.521222514	0.001375584	0.074420213
PPARGC1A	6.837713953	-3.514484947	0.001400566	0.075054445
ANKZF1	7.834707411	3.511865783	0.001410395	0.075092343
GPRASP1	8.936204817	-3.508914842	0.001421549	0.075387068
CERK	9.034787896	-3.502518216	0.001446022	0.076382971
RBM6	8.318188157	3.496794153	0.001468266	0.076952053
NDUFB5	9.689702685	-3.489809635	0.001495857	0.077235454
CADPS	7.649932901	-3.489598258	0.0014967	0.077235454
OPA1	8.00376988	-3.484261325	0.001518131	0.077928716
MAT2A	8.824407082	3.482797792	0.001524059	0.077928716
NAP1L2	8.81342825	-3.47534072	0.001554616	0.078120904

SMS	7.906002733	-3.466420522	0.001591946	0.078987823
SHC2	8.90288708	3.460540876	0.001617023	0.079049604
GUCY1B3	7.560413601	-3.455849231	0.001637306	0.079143092
SMAP1	8.086208921	-3.454965984	0.001641152	0.079143092
RET	7.443319943	-3.454850757	0.001641655	0.079143092
PRKAR2B	7.514696069	-3.453027319	0.001649625	0.079143092
PIP5K1B	6.980877434	-3.451224798	0.00165754	0.079143092
ADAM23	7.700208544	-3.445650674	0.001682249	0.079320397
PKIA	8.636114939	-3.444401682	0.001687834	0.079320397
FKBP1B	9.200794921	-3.443860402	0.00169026	0.079320397
SLC30A9	8.71797091	-3.437687488	0.001718166	0.07976688
RHOG	9.489745311	3.431526109	0.001746462	0.079973665
FOXO3	10.17356629	3.418080327	0.001809782	0.082074749
RABGGTB	9.081623083	-3.416022386	0.001819667	0.082074749
COPG2IT1	9.503569816	-3.415864657	0.001820426	0.082074749
HPRT1	8.466037925	-3.408280556	0.00185732	0.082767724
SLIRP	9.887089926	-3.396136502	0.001917901	0.083411482
NES	9.586035682	3.395231744	0.00192249	0.083411482
ERC2	7.077116344	-3.391765675	0.001940168	0.083411482
ISCU	10.562487	-3.390873923	0.001944741	0.083411482
NDUFS3	10.00442101	-3.390209509	0.001948155	0.083411482
TUBB2A	12.34854386	-3.390122581	0.001948603	0.083411482
GALNT11	9.068275173	-3.387274999	0.001963304	0.08362429
PFKM	10.53262959	-3.382457889	0.001988417	0.08442594

UQCRC2	9.04023584	-3.379760123	0.002002617	0.084760594
RAN	9.372269138	-3.377884721	0.002012545	0.084912946
NOP2	8.583992815	3.374356381	0.002031353	0.085225036
ACOT13	8.042596242	-3.372572534	0.002040925	0.085305599
PSMB6	9.90206072	-3.368560548	0.002062614	0.085944406
NELL2	9.133586748	-3.366568875	0.002073464	0.086128991
ACAT1	9.571481499	-3.362971276	0.0020932	0.086413756
C4orf27	8.856027685	-3.357030262	0.00212619	0.086594118
UQCRFS1	10.34420423	-3.355909511	0.002132469	0.086594118
CXorf40A	8.808056089	-3.351714363	0.002156131	0.086594118
ARHGEF18	9.59266055	3.349750921	0.002167292	0.086594118
BCAT2	7.591310897	3.348520613	0.002174314	0.086594118
ATP5F1	10.51985701	-3.345782285	0.002190022	0.086933497
APOO	8.796416213	-3.340823587	0.002218744	0.087540081
ILK	9.782215899	3.331893947	0.002271383	0.08807844
EFHD1	11.25191026	3.326565702	0.002303361	0.088900174
SMPX	6.714099751	-3.324721991	0.002314527	0.088900174
EIF4E	7.407320203	-3.324186436	0.00231778	0.088900174
LYPLA1	6.597158239	-3.317914363	0.002356207	0.089695515
PCDH8	5.635599572	-3.317319999	0.002359881	0.089695515
MYL9	7.075326612	3.307724735	0.002419944	0.091137797
CYP27A1	9.4302445	3.304921041	0.00243777	0.091361898
DDX25	7.787781437	-3.299802764	0.002470637	0.092079277
PDIA3	8.324336455	-3.292639482	0.002517352	0.093072237
RBM10	8.430909915	3.289664809	0.002536999	0.093257287
VAV3	7.112447666	-3.281824978	0.002589486	0.094926594

NME7	7.739306664	-3.268979737	0.002677743	0.096216159
GLO1	10.24890912	-3.268767794	0.002679223	0.096216159
ABCA8	9.669822218	3.268365656	0.002682034	0.096216159
NDE1	9.435414895	3.261042076	0.002733715	0.097289793
ATP8A2	8.356288472	-3.25749148	0.002759113	0.097933915
USP16	8.600611328	-3.252548671	0.002794847	0.098680159
LAPTM4B	9.155909014	-3.249462774	0.002817381	0.099214685
POLR2K	7.545572217	-3.24741653	0.002832418	0.099483136
SULT4A1	9.112334588	-3.241221663	0.002878415	0.100572108
FIBP	9.316153611	-3.238897115	0.002895858	0.100918774
SNAP25	9.393211624	-3.227000302	0.002986728	0.1035632
PSRC1	9.777667673	3.226942321	0.002987177	0.1035632
ADAP1	9.446379955	3.221210971	0.003031929	0.104306127
C5orf22	7.776917197	-3.209596603	0.003124591	0.106016289
SAMM50	9.392981407	-3.194698123	0.003247425	0.108655121
CLDN10	8.201683234	-3.185873514	0.003322344	0.110610159
PALM	9.124858181	3.180916087	0.003365153	0.110934309
DDC	6.632882341	-3.170766312	0.00345445	0.112349343
CDK5RAP3	9.553053337	3.162150439	0.003532022	0.113916208
ORC3	6.833335844	-3.15371079	0.003609618	0.115452502
KLK6	9.664862703	3.153375253	0.003612737	0.115452502
CDKN1C	8.214603451	3.153231284	0.003614075	0.115452502
BBOX1	8.728395456	-3.148767854	0.003655817	0.116200963
VCAM1	8.227741316	-3.147953013	0.003663487	0.116200963
ACTR10	9.958805116	-3.141876026	0.003721174	0.117475272
GABRB1	10.20306296	-3.137350106	0.003764699	0.118016268
NEFL	8.841972378	-3.126084602	0.003875154	0.11979364
TPBG	8.794944629	-3.12318351	0.003904095	0.11979364

		6		
ADAMTS9	7.424673394	3.122948202	0.003906451	0.11979364
MRPL15	8.118862829	-3.122639222	0.003909547	0.11979364
TMEM14B	7.175695434	-3.119007376	0.003946117	0.120588975
SNCA	8.563316998	-3.118167414	0.003954621	0.120588975
SLCO4A1	8.606309766	3.109544228	0.004042939	0.122738098
COMMD8	7.796894015	-3.106753913	0.004071918	0.123047123
IMMT	9.10583996	-3.103532852	0.004105616	0.123542372
EBLN2	8.266537301	3.101853233	0.004123293	0.123542372
MYO18A	8.485751075	3.100389189	0.00413876	0.12367425
MLLT11	11.28734581	-3.096954436	0.004175264	0.12383215
LMO3	11.05030942	-3.096420874	0.004180962	0.12383215
TRIM37	10.12310269	-3.095181995	0.004194221	0.12395123
FBXW4	9.773859561	3.092927573	0.004218451	0.124259317
AASDHPPT	7.886078246	-3.091368797	0.004235282	0.124343068
CRYZL1	7.689937865	-3.087216696	0.004280428	0.124829783
PTPRK	9.968454161	3.087033819	0.004282427	0.124829783
SUB1	8.057443206	-3.086262865	0.004290864	0.124829783
VPS26A	10.0861512	-3.085601233	0.004298117	0.124829783
DGKD	9.231531094	3.085577261	0.00429838	0.124829783
SCAF8	7.395180378	-3.084064019	0.004315013	0.124886168
SLC25A5	10.76778074	-3.083480368	0.004321445	0.124886168
PSMB5	10.26869137	-3.082864114	0.004328245	0.124886168
LOC101060275	9.488797944	3.082004009	0.004337754	0.124891953
GPM6A	10.26652132	-3.080052468	0.004359404	0.124978883
UBE2K	7.058855549	-3.077509284	0.00438777	0.125179043
ELAVL2	5.37152282	-3.07143443	0.004456241	0.126521791

		5		
PBLD	6.682768751	-3.066302704	0.004514873	0.127190452
PLEKHB2	8.987286453	-3.064864082	0.004531441	0.127193175
XPA	7.933296312	-3.062628541	0.004557301	0.127240962
BDNF	5.765184012	-3.062258584	0.004561594	0.127240962
FTCD	7.529359801	3.059199937	0.004597234	0.127488607
CCDC6	5.852519563	-3.058384848	0.004606777	0.127488607
DLK1	7.905428376	-3.058239537	0.00460848	0.127488607
ASTN2	7.125197163	-3.056275759	0.004631555	0.127863333
CCT2	8.541107292	-3.053845812	0.004660261	0.128128523
COL9A1	7.632775978	3.049796156	0.004708476	0.128925756
OXR1	5.65992536	-3.04667901	0.004745911	0.129534629
PTGDS	12.06928196	3.042175751	0.004800491	0.129710943
MYH9	8.24475248	3.042091621	0.004801517	0.129710943
RGL2	9.462782103	3.040517982	0.004820734	0.129710943
CEBPD	7.70277096	3.034760214	0.00489167	0.130221308
VBP1	10.12018234	-3.031877012	0.004927563	0.130658322
CUX2	5.868857809	-3.020401315	0.005072914	0.131766774
ATP6V1H	9.616267011	-3.018323508	0.005099663	0.131766774
CERS1	10.64138046	3.017376885	0.005111894	0.131766774
CCDC28A	8.392855337	-3.009026452	0.005220995	0.132244871
GSTM3	8.918657574	-3.001991671	0.005314617	0.133219915
XK	6.059558649	-3.001438552	0.005322045	0.133219915
SLC7A5	9.624919674	2.999107237	0.005353462	0.133556128
TPST2	7.232600927	2.99873952	0.005358433	0.133556128
ST6GALNAC5	6.196288762	-2.997498616	0.005375242	0.133556128
CKLF	6.817741434	-2.996656833	0.005386673	0.133591476
RPF1	7.693005358	-2.99502758	0.005408862	0.133894294

		3		
SRD5A1	6.646737982	-2.989531536	0.005484357	0.13476852
ALDH1A1	7.307383482	-2.986560086	0.005525587	0.135519807
DLD	8.205266585	-2.985875251	0.005535131	0.135519807
CPNE3	7.234635638	-2.983366077	0.005570233	0.135707016
HSPB1	10.37243378	2.983160295	0.005573121	0.135707016
SLC35G2	7.866944846	-2.977269479	0.005656401	0.136153643
LIN7B	8.671391267	-2.975988972	0.005674659	0.136153643
DUSP1	8.303422905	2.975419726	0.005682793	0.136153643
INSM1	6.739477468	-2.965992016	0.005819129	0.138316847
TSPAN13	9.159053655	-2.96491252	0.005834936	0.138316847
RNF128	7.072450509	-2.964099936	0.005846861	0.13835509
STAT3	9.145171813	2.962192211	0.005874949	0.138774981
HMP19	9.077134108	-2.958352745	0.005931865	0.139383251
GPC5	7.320991604	-2.953910851	0.005998362	0.139965268
PDSS2	6.827266096	-2.952536764	0.006019075	0.14020475
COX11	6.739718951	-2.95020558	0.006054371	0.140511554
UBE2V2	7.965168035	-2.948905613	0.006074137	0.140511554
NMNAT2	8.375088892	-2.945800391	0.006121602	0.1411229
RPP40	7.05892968	-2.944952415	0.006134624	0.141180529
FBXL14	8.139306161	2.94035705	0.006205651	0.141493832
GCLM	7.080754177	-2.938256405	0.006238377	0.14186493
NSG1	8.458008273	-2.928642803	0.00639024	0.14337433
PSMB4	9.891724926	-2.927425183	0.006409721	0.143571339
DIP2A	10.90420593	2.921444152	0.006506232	0.144165747
WDR7	9.609345243	-2.921041461	0.006512779	0.144165747

KIF3A	5.840131247	-2.920811208	0.006516526	0.144165747
STX7	8.103869709	-2.917336863	0.006573301	0.144165747
GRIA1	6.398160894	-2.914632247	0.006617821	0.144847145
NANS	8.072886403	-2.913271867	0.006640321	0.144866965
NEFH	10.09409097	-2.907989827	0.006728369	0.145214229
ICAM2	8.755077704	2.906590839	0.006751873	0.145214229
NIT2	7.786331864	-2.906548636	0.006752584	0.145214229
NEFM	9.940637293	-2.904768884	0.0067826	0.145214229
GTF2B	8.504852718	-2.902023332	0.006829151	0.145817218
ANKRD46	8.645093174	-2.900655967	0.006852447	0.145817218
ATP5L	10.14808346	-2.898882521	0.006882774	0.146117364
TAZ	7.926922575	2.893129555	0.006982024	0.14706093
RFK	7.207963892	-2.891518842	0.007010052	0.147419866
SIDT2	10.14641522	2.887491294	0.007080602	0.14843819
ANK1	7.131479607	-2.885429864	0.00711697	0.148735812
NECAP1	8.345896206	-2.882468724	0.007169519	0.149310024
MYRIP	7.511084335	-2.881700592	0.00718321	0.149310024
NDUFA5	7.281888139	-2.881196784	0.007192203	0.149310024
SEPP1	12.31134871	2.880459167	0.007205389	0.149310024
H1FX	6.637291647	2.875221471	0.007299675	0.150676532
OR7A5	7.849020775	2.870515447	0.007385381	0.151514485
TPM2	6.103269476	2.869895869	0.007396735	0.151514485
AP5M1	8.490971984	-2.868473223	0.007422867	0.151818
MICALL2	7.702067807	2.865355448	0.007480442	0.151956398
NPTX2	8.071645827	2.864407187	0.007498036	0.151956398
TAGLN3	9.88223013	-2.857835206	0.007621048	0.153300755
MDH2	9.09333515	-2.85542144	0.007666703	0.153528588

		2		
MRPS28	7.075954897	-2.851937279	0.007733057	0.153787014
P2RX5	7.337500491	2.847836947	0.007811838	0.154817479
COPS5	9.101471293	-2.846702242	0.007833773	0.155023194
FAM98A	9.521441012	-2.845660255	0.007853966	0.155182532
AGTR1	6.127822945	-2.844270828	0.007880968	0.155182532
LPPR4	7.335636247	-2.833189569	0.008099466	0.156215307
WDR47	8.58653052	-2.831819355	0.008126875	0.156215307
SEC22B	8.563191482	-2.826825039	0.00822752	0.157698045
PJA2	8.915671206	-2.822812418	0.008309228	0.157910638
HK1	10.28222189	-2.819868579	0.008369656	0.15883406
TUBA4A	9.625854648	-2.813385911	0.008504184	0.160224219
MTMR6	7.624244437	-2.812689609	0.008518754	0.160224219
KIAA0196	7.553414966	-2.811844116	0.008536477	0.160224219
LRP10	8.885504551	2.807158949	0.008635313	0.1609423
SH3GL2	9.744448172	-2.805681801	0.008666697	0.1609423
CNIH3	6.002316674	-2.804626924	0.008689174	0.1609423
PLCB4	6.273353741	-2.804520193	0.008691451	0.1609423
HMGB2	7.76641547	-2.803257816	0.008718428	0.1609423
GAL3ST1	7.316195604	2.802066155	0.008743966	0.1609423
AGPAT5	8.477789882	-2.79278538	0.008945271	0.163029333
SEH1L	8.825887957	-2.78998758	0.009006804	0.163029333
NDUFB1	11.44643257	-2.789570676	0.009016007	0.163029333
RAMP3	9.264847232	2.786112648	0.009092682	0.163406111
UQCRB	9.774840538	-2.782186335	0.00918048	0.164671786
DPY19L2P2	6.132303771	-2.78141073	0.009197917	0.164764288

3

CSF1R	8.896112216	2.779037816	0.009251458	0.165232772
ADIPOR2	11.40555566	2.778498902	0.009263659	0.165232772
CAPZA2	7.803486579	-2.776192469	0.009316044	0.165232772
EDNRB	8.210415386	-2.775427403	0.009333482	0.165232772
STOML2	7.761515052	-2.770940602	0.009436364	0.166370168
LDB3	8.274260112	2.768759151	0.009486768	0.166820393
FRYL	8.227719665	2.765166687	0.009570322	0.167529419
SCRN1	11.26906035	-2.753384516	0.009849213	0.171174724
RCAN2	11.07603722	-2.751498365	0.009894558	0.171175486
PREPL	8.15333573	-2.743688429	0.010084405	0.17305905
SLC31A2	9.489602645	2.743086404	0.01009918	0.17305905
EMC7	10.08492774	-2.735138691	0.010296134	0.174280832
PIEZO2	7.783954545	2.73435292	0.0103158	0.174280832
DUSP8	7.980143368	2.733918229	0.010326695	0.174280832
NLK	6.389406255	-2.727975959	0.010476703	0.17530684
UBA3	8.575749863	-2.727887533	0.010478951	0.17530684
SRP19	6.67792691	2.72311483	0.01060093	0.176030537
TPCN1	9.849577403	2.720631087	0.010664931	0.1764382
NRIP3	8.492748082	-2.718802664	0.010712276	0.177003216
PCDH9	11.34824277	2.71178666	0.010895775	0.178739733
MAFF	8.513820952	2.710766405	0.010922701	0.178739733
ESRRG	6.628920005	-2.708232686	0.010989841	0.179174664
ANKRD49	7.92827147	2.704924513	0.011078081	0.180163164
DDHD2	8.971234608	-2.70346458	0.011117232	0.180362636
TMEM5	7.226379968	-2.697875518	0.011268309	0.181496874
PAN2	7.76305692	2.696792114	0.011297814	0.181753927
PEG10	7.578824252	-2.69625485	0.011312473	0.181771797
SLC9A6	9.631474493	-2.693987614	0.011374528	0.182332181
CLASRP	9.938844613	2.692895299	0.011404537	0.182449743
IFT57	6.441960258	-2.69117180	0.011452038	0.182813897

		1		
PYGL	6.806441347	-2.686563916	0.011579941	0.183650195
HEXB	8.647958113	-2.685258201	0.011616425	0.184011305
UHRF1BP1L	5.732543164	-2.684745911	0.011630769	0.184021254
SACM1L	8.894252248	-2.683584732	0.011663342	0.184179795
PCMT1	8.923451235	-2.683009978	0.011679496	0.184179795
CDC37	6.870818216	2.681168826	0.011731384	0.184179795
CAPNS1	9.77255521	2.68037135	0.011753925	0.184179795
TENM1	6.347707853	-2.679113905	0.01178955	0.184179795
PLXDC2	5.957132159	-2.675844097	0.011882658	0.18450413
SYNJ1	6.439734725	-2.673195402	0.011958581	0.184657163
ACTR3	9.666989325	-2.672872222	0.011967875	0.184657163
CHGB	9.021514668	-2.672668259	0.011973745	0.184657163
GNG3	9.392704423	-2.668778624	0.012086189	0.185114605
COL4A2	7.0898638	2.66703073	0.012137037	0.185469962
MIR6872	8.275864408	2.661222518	0.012307437	0.187433469
PAGR1	8.690071992	2.65382695	0.012527626	0.189069917
RAPGEF3	8.162385895	2.647369365	0.012722875	0.191156571
C11orf58	9.171085444	-2.644728091	0.012803547	0.192153463
BASP1	11.38272352	-2.640643059	0.012929252	0.192746415
SLC4A2	7.20244641	2.640050001	0.012947596	0.192805662
ACOT7	7.839788435	-2.638852624	0.012984707	0.192999584
SYBU	9.386772351	-2.638701819	0.012989388	0.192999584
RBM3	5.744195359	-2.637238817	0.013034881	0.193247517
ARL6IP5	10.07941512	-2.632362365	0.013187587	0.19443721
LRRC32	9.228825053	2.631847451	0.013203809	0.194462677
RAD54L2	8.199727859	2.626881919	0.013361188	0.194643926

EBNA1BP2	7.038253612	-2.619619268	0.013594508	0.196015536
SYNGR3	10.33653631	-2.618946048	0.013616326	0.196015536
RNF14	6.924289086	-2.617601859	0.013659986	0.196015536
MKNK2	9.527754919	2.61715641	0.013674483	0.196015536
VWA1	7.417430679	2.612956121	0.013811885	0.196743532
GJA4	7.737007459	2.610710859	0.013885856	0.197358612
LRPPRC	8.615047122	-2.605909041	0.014045288	0.198155235
TMEM35	7.301642286	-2.60499303	0.014075894	0.198372396
COL9A2	8.395770177	2.602497027	0.014159604	0.19870871
DNAJA2	6.451574769	-2.595923295	0.014382281	0.199891263
CDC42BPB	7.069991644	2.595644003	0.014391813	0.199891263
PGF	8.507360803	2.593337352	0.014470762	0.200572536
CUL3	7.136038647	-2.592011473	0.014516323	0.2009964
PHIP	8.492482338	2.590315771	0.014574787	0.201073047
SNAP91	8.977355156	-2.589903025	0.01458905	0.201073047
ATP1B2	8.811871304	2.585451686	0.014743698	0.201981275
DDX1	9.595693623	-2.584643917	0.014771923	0.202033528
ZNF408	8.106285942	2.580773388	0.014907863	0.20244818
CNN3	7.799434953	-2.579824339	0.014941372	0.202698063
MYLK	10.76028484	2.57407888	0.01514572	0.204642627
SHOC2	7.60631306	-2.570869696	0.015260982	0.205372718
RGS7	8.736413189	-2.568726931	0.015338393	0.205384452
NFU1	7.060522156	-2.555141265	0.015837697	0.208758877
KAL1	8.637255508	-2.550403921	0.016015303	0.209624545
AP1AR	5.422241574	-2.546727141	0.016154413	0.210430831
HRASLS	6.374380631	-2.54305275	0.016294545	0.210769437
ANKRD34C	7.990282412	-2.541594817	0.016350456	0.210838931
AHNAK2	7.499891039	-2.540585484	0.016389268	0.210838931

MFSD12	7.306780071	2.540190063	0.016404496	0.210838931
TUBB6	8.174098415	2.538993377	0.016450661	0.210838931
EFS	8.730993706	2.538935801	0.016452885	0.210838931
STMN2	10.12484803	-2.537251893	0.016518057	0.211069308
COX5A	10.12860222	-2.530775881	0.01677092	0.212679998
MMADHC	9.189593181	-2.529973649	0.016802491	0.212685228
IMPACT	5.882942995	-2.526561481	0.016937387	0.21398203
DERA	6.982668902	-2.519621338	0.017214838	0.216299907
SDC1	6.874293989	-2.516515572	0.017340349	0.216827083
HLA-DRB4	6.397197954	2.515436702	0.017384145	0.217172324
PTOV1	8.308868924	2.513709223	0.017454484	0.217430435
TCEB1	7.516001126	-2.507411202	0.017713146	0.218896022
RCN2	7.030124647	-2.506371398	0.017756189	0.219167234
HERC2P2	10.01230377	2.504109892	0.017850136	0.219747905
EFR3A	8.736684971	-2.499840337	0.018028748	0.219909545
SPCS1	10.530601	-2.49961966	0.018038025	0.219909545
STAT6	8.070752131	2.496257607	0.018179894	0.220389747
LOC100996756	6.4822281	2.493083189	0.018314786	0.221377917
ATMIN	8.169778078	-2.490700734	0.018416629	0.222068301
INPP4B	5.63151609	-2.490539979	0.018423519	0.222068301
ARMCX1	8.443870417	-2.488274795	0.018520864	0.222068301
FGF9	7.881681418	-2.485786597	0.018628337	0.222854889
AEBP1	8.350615246	2.485234371	0.018652266	0.222854889
PFKP	9.348085818	-2.48458158	0.01868059	0.222854889
NQO2	8.248488516	-2.481401309	0.018819145	0.223070849
TSNAX	6.95484783	-2.480412745	0.018862406	0.223368844
PDHX	8.147861655	-2.478676308	0.018938615	0.223778845
UQCRH	10.08336523	-2.47518197	0.019092833	0.224811527

		6		
DNAJB9	7.699895584	-2.472823188	0.019197585	0.224955452
PSMD10	9.165214238	-2.468155482	0.019406429	0.225521066
RTCA	7.537866319	-2.467614945	0.019430749	0.225521066
CRIP2	7.22178721	2.466716093	0.01947125	0.225795821
ZNF160	10.63017999	2.465252665	0.019537357	0.226108914
MFSD6	4.843813965	-2.464592165	0.019567261	0.226127425
FRRS1L	8.165984791	-2.463330585	0.019624495	0.226388734
ACTR6	7.324559802	-2.461441694	0.019710475	0.226417334
CACNA2D3	6.120630448	-2.458273824	0.019855448	0.226966689
HMGCR	7.517585779	-2.458179134	0.019859797	0.226966689
NASP	8.05879112	2.455800164	0.019969331	0.227443556
NUPR1	7.899633154	2.453031977	0.020097481	0.228321683
CBLN1	6.152529285	-2.452011723	0.020144903	0.228473509
MYO1C	9.922293968	2.448737018	0.020297804	0.229819103
ELOVL6	6.055008531	-2.448139521	0.020325817	0.229942227
NPTN	9.8309447	-2.443403305	0.02054912	0.231368015
S100A10	10.0142928	2.440895383	0.02066827	0.231565155
SLC38A6	6.259953173	-2.438006935	0.020806283	0.232239395
PSMD6	9.638066992	-2.437294694	0.020840444	0.232239395
NDFIP1	8.11949875	-2.434431491	0.020978288	0.233001396
TH	7.188516461	-2.430566673	0.021165675	0.233973206
STYK1	5.028269765	-2.427214956	0.021329423	0.234522272
COL16A1	9.044700394	2.427070332	0.021336514	0.234522272
GABARAPL2	11.65895409	-2.425685218	0.021404542	0.234522272
CDK7	6.846430507	-2.425170927	0.021429851	0.234522272

CCT7	9.821447896	-2.422810407	0.021546366	0.235137012
CSRNP3	6.099598047	-2.421289592	0.02162174	0.235586503
CD151	8.1021776	2.416337961	0.021868822	0.236560761
FBXL15	7.96354601	-2.416274776	0.021871991	0.236560761
ACTR3B	6.380331534	-2.415804309	0.021895604	0.236560761
PCSK2	6.420899295	-2.411128956	0.022131527	0.237654669
C1QBP	8.001862323	-2.41063787	0.022156441	0.237654669
MCTS1	7.746032249	-2.410589393	0.022158902	0.237654669
CD74	10.0521386	2.409634083	0.022207447	0.237985081
IGFBP4	7.432236254	2.404173168	0.022486818	0.238480386
NME3	8.897975039	2.40289324	0.022552759	0.238480386
ARMC2-AS1	10.32764629	-2.402709871	0.02256222	0.238480386
BHMT	8.787557028	2.402544163	0.022570774	0.238480386
LENEP	7.73886079	2.399134023	0.022747452	0.238633684
UGP2	9.555372971	-2.397235696	0.022846349	0.23886556
DYNC1I1	10.40878355	-2.396191606	0.02290091	0.23886556
FAM65A	9.108477371	2.396100108	0.022905697	0.23886556
FAM171A1	10.27779906	2.394971259	0.022964832	0.23886556
INSIG2	6.582119108	-2.39424425	0.02300299	0.23886556
BCAS2	8.563331376	-2.393198082	0.023058001	0.23886556
PNMAL1	9.386986935	-2.392799163	0.023079009	0.23886556
GOT1	8.083280496	-2.389907553	0.023231811	0.23977016
NUCB2	6.999654024	-2.383672843	0.023564414	0.241715395
NAA50	8.0001955	-2.382158945	0.023645827	0.242365211
RBFOX1	7.668276047	-2.369553386	0.024333728	0.246263235
HIST1H1C	7.867440571	2.369155599	0.024355729	0.246263235
TRAPPC8	8.854836009	-2.367259156	0.024460868	0.246575106
TAGLN	8.856960268	2.362019151	0.024753523	0.247599692

RIT2	6.604777005	-2.358531888	0.024950047	0.247599692
AQP4	7.071769292	-2.356806003	0.025047833	0.247674037
TXNDC9	7.369739811	-2.356445589	0.025068297	0.247674037
MBP	11.88567708	2.354822962	0.025160618	0.248314056
ADAMTS1	8.274094057	2.354656768	0.025170091	0.248314056
MRPL3	9.275547577	-2.351033853	0.025377407	0.248568717
TTYH1	8.480360451	2.346534155	0.025637054	0.249607471
SEC61A1	8.219481856	2.34539072	0.025703417	0.249607471
BATF3	7.770986616	2.343881622	0.025791242	0.249607471
DYNC1LI1	7.458146249	-2.341231075	0.025946153	0.250609093
S100A1	8.7467278	2.339312482	0.026058812	0.250609093
MBD3	8.492376313	2.33753144	0.026163791	0.250609093
EPHX1	7.476097178	2.336945154	0.026198432	0.250609093
JMJD6	7.620831467	2.336624404	0.026217402	0.250609093
SERPINE2	10.73046006	-2.336503109	0.026224578	0.250609093
TRIM36	6.499002373	-2.335074941	0.026309213	0.250642008
RANBP6	8.438070171	-2.325668658	0.026872853	0.253514994
CEP85	8.432315731	2.320753102	0.027171734	0.254623977
EAPP	7.677471861	-2.315783	0.02747699	0.255848513
ASNSD1	8.448989083	-2.315142733	0.027516539	0.255848513
CDO1	7.468885689	-2.313926066	0.027591834	0.255995838
APMAP	10.67606317	-2.313736408	0.027603588	0.255995838
CCK	6.886918833	-2.313653891	0.027608704	0.255995838
LOC100506248	10.89274251	2.312021358	0.027710084	0.256081233
SMPDL3A	6.120640276	-2.311790496	0.027724448	0.256081233
FAM169A	5.749958928	-2.311755301	0.027726638	0.256081233
SEC14L5	10.01614773	2.310599583	0.027798651	0.256081233
SCG3	5.634943174	-2.310545337	0.027802035	0.256081233
INA	10.00120891	-2.30924734	0.027883124	0.256589765

		3		
ELAVL4	9.237635187	-2.306073194	0.028082323	0.257016678
UNC50	8.471490278	-2.305658981	0.028108412	0.257016678
PSMD12	7.808456891	-2.30266791	0.028297457	0.257899234
ATP6V1G2	8.606927006	-2.302182762	0.028328228	0.257899234
PKD1P1	7.121350812	2.301502671	0.028371415	0.257899234
KIAA1107	6.693754936	-2.300354228	0.028444477	0.258388318
MGP	6.695969527	2.29903355	0.028528707	0.258628148
CLGN	5.22508705	-2.297374064	0.028634865	0.259245181
VIPR1	8.149798167	2.297064036	0.028654737	0.259245181
ACTR2	8.046784073	-2.296004274	0.028722759	0.259685488
COL4A1	7.579521209	2.294334725	0.028830218	0.259956344
CST3	10.33782194	2.291500344	0.029013483	0.260945829
ISG20	7.047185063	2.291496412	0.029013738	0.260945829
EMP3	7.16635416	2.290813801	0.029058031	0.261121934
COX17	8.70044412	-2.288508578	0.029208064	0.261946141
THY1	8.933001076	-2.288224429	0.029226606	0.261946141
LINC00094	7.701845373	-2.285910645	0.029377986	0.26207742
PPP3CB	8.808266056	-2.28432162	0.029482358	0.262484938
SMEK2	5.432780926	-2.279059783	0.029830366	0.263675717
FKSG49	9.578279759	2.278647825	0.029857768	0.263675717
ZNF204P	6.48250839	-2.277546583	0.02993113	0.263675717
KIFAP3	8.331692927	-2.270152083	0.03042796	0.265616093
CHRM2	7.480097808	2.268078707	0.030568596	0.266496983
GNAI2	8.66937746	2.266405468	0.030682519	0.266623934
UBA1	8.773632348	2.258869152	0.031200385	0.268171403
ZNF324	8.503595148	2.25695525	0.031333148	0.268795935
TTBK2	5.797222407	-2.256036556	0.031397056	0.268881064
MIR6883	9.039890968	2.255110461	0.031461598	0.268881064
CD24	6.759737035	-2.251810313	0.031692565	0.268987905

TRAPPC10	6.987749591	2.250633207	0.031775316	0.268987905
NACC2	10.63695818	2.248146877	0.031950745	0.269781714
PHLDB1	7.563309424	2.245243785	0.032156681	0.270534794
UBQLN4	8.612570049	2.24084125	0.032471262	0.271394637
SLC25A3	11.15599895	-2.240077837	0.032526092	0.271394637
ELOVL4	5.423927536	-2.238318022	0.032652803	0.27177584
WWP1	6.730419237	-2.235888418	0.032828469	0.272010725
RWDD2A	6.173241961	-2.231721586	0.033131718	0.272215066
ZNHIT6	7.234665617	-2.231306767	0.033162045	0.272215066
CMAS	6.030642542	-2.224741846	0.033645324	0.273379866
NAP1L3	8.907339032	-2.224261642	0.033680921	0.273379866
ANKMY2	7.732378919	-2.223130995	0.033764869	0.273472879
LZTFL1	6.702517937	-2.220936416	0.033928347	0.273472879
LBH	7.857142146	-2.219656971	0.034023983	0.273472879
TAOK1	9.397723172	2.218791857	0.034088785	0.273472879
PSMC6	7.178556569	-2.218769776	0.034090441	0.273472879
ILF2	7.412650534	-2.218761353	0.034091072	0.273472879
IFITM3	11.92867335	2.217456851	0.034189003	0.273472879
TIPRL	7.711054816	-2.217453097	0.034189285	0.273472879
PCSK1	5.851966784	-2.213445796	0.0344917	0.274318396
TGM5	8.212637951	2.212324359	0.034576758	0.274400561
NOC3L	6.677175836	-2.203833891	0.035226868	0.276882768
ZNF721	11.01892225	2.202063303	0.035363812	0.277431732
MAST3	8.813598651	2.199991669	0.035524646	0.277726772
BAIAP3	8.285718338	2.193500618	0.036032837	0.279290911
DNM1	10.21377967	-2.192406879	0.036119104	0.279636478
FBXO17	7.026311434	2.191306671	0.036206068	0.279912259
ZNF862	8.876292954	2.190985586	0.036231483	0.279912259

TNXA	7.641738135	2.190533409	0.036267301	0.279912259
SCP2	8.566586378	-2.18223293	0.036930457	0.281967553
TRAPPC2	5.530029993	-2.180768638	0.037048563	0.281967553
APOD	11.24175867	2.173911513	0.037606144	0.282983361
OPHN1	10.72515543	2.166288009	0.038234828	0.28515658
VWF	10.1945688	2.165390883	0.038309424	0.285395639
QPCT	5.265954726	-2.164431653	0.038389328	0.285832195
NAA15	5.986677291	-2.163912192	0.038432661	0.285996127
TGFB1I1	7.257296714	2.162501557	0.038550556	0.286414898
RASSF2	10.61157079	2.160275845	0.038737227	0.287184304
ARL6IP1	10.07145155	-2.156474191	0.039057938	0.288270918
CCDC94	7.458014592	2.155162558	0.039169136	0.288270918
CHL1	8.264975564	-2.155036264	0.039179858	0.288270918
SPATA2L	7.641882186	2.151570355	0.039475124	0.288278972
FOCAD	7.480273134	-2.149354268	0.039664953	0.288759998
ZBTB20	9.041785238	2.148290554	0.039756359	0.288940484
PCP4	10.01507909	-2.146989647	0.039868402	0.288940484
FA2H	8.712366374	2.141135674	0.040376073	0.289259967
NDUFC2	10.11964754	-2.139329653	0.040533852	0.289259967
TSPAN5	6.907839882	-2.138567998	0.040600557	0.289259967
EN1	5.55603992	-2.132893183	0.041100633	0.289876317
MRPL13	6.041546966	-2.130186497	0.041341074	0.2899578
CDKN1A	9.533372323	2.128113245	0.041526091	0.2899578
PEG3	8.246768996	-2.127881584	0.04154681	0.2899578
FABP5	8.539881921	-2.126806355	0.041643096	0.2899578
NETO2	6.491964113	-2.125596374	0.041751685	0.290099618
AUH	7.785909331	-2.114761548	0.04273532	0.294342806
CAB39	9.264924722	-2.112751008	0.042920091	0.294989684

DPM1	7.824878876	-2.111853097	0.043002839	0.294989684
KCTD9	5.824145562	-2.102190054	0.043902344	0.297794613
RTN1	10.54342592	-2.100815048	0.044031685	0.297996377
CAPN3	9.280201579	2.100025737	0.044106085	0.298088589
HSPA6	7.451309327	2.098439518	0.044255937	0.298183101
ISOC1	7.797762617	-2.098411698	0.044258569	0.298183101
SNX7	8.110112484	-2.092778155	0.044794458	0.299157412
AKAP2	7.795435548	-2.092145368	0.044855009	0.299263876
CCT4	10.87327204	-2.086835544	0.045365964	0.299836249
PNMA1	9.209553501	-2.085582975	0.045487244	0.299836249
CDC37L1	6.268402443	-2.082984472	0.04573976	0.299836249
IFITM1	10.42528736	2.082305119	0.045805982	0.299836249
GLRB	6.46637876	-2.082035152	0.045832321	0.299836249
PPIB	9.371375977	-2.08137382	0.045896901	0.299836249
SUCLA2	7.327749787	-2.07518155	0.046505493	0.301912346
NFKBIA	10.48214262	2.073661867	0.046655936	0.302115202
ASGR2	7.729903462	2.072030558	0.046817909	0.302434224
TMEM100	6.951208813	-2.071653357	0.046855432	0.302530956
LYL1	7.447286622	2.070807063	0.046939715	0.30263823
LACTB2	6.116807023	-2.069732617	0.047046913	0.303038136
SPATA7	5.485001115	-2.068052618	0.047214961	0.303683191
TTC19	8.800919412	-2.065920067	0.04742904	0.304125517
BPGM	6.184965013	-2.057457766	0.048287006	0.305166658
EPCAM	5.469047311	-2.056327128	0.048402668	0.305326402
GAP43	7.950590641	-2.055884224	0.048448043	0.305326402
EGR3	5.244901965	-2.0523976	0.048806553	0.306314117
TMEM30A	7.561425124	-2.05079319	0.048972308	0.30650265

		7		
FAM188A	6.896051261	-2.04373672	0.049707233	0.306807729
CRISPLD2	9.130608587	2.042821392	0.049803272	0.306807729
AGL	7.922709346	-2.042812815	0.049804172	0.306807729
SUCLG2	6.444056672	-2.042809511	0.049804519	0.306807729

Supplementary table 5. List of ferroptosis-related genes

Ferroptosis-related genes 1	Ferroptosis-related genes 2	Ferroptosis-related genes 3
AKR1C1	CCT5	AIFM2
CYB5R1	HP	GPX4
ABCB10	EIF4A1	TP53
MIR326	TUBB4A	ACSL4
NEDD4	VAPA	SLC7A11
ABCB6	XRCC5	TFRC
NEDD4L	RUVBL1	HMOX1
LTF	RPA2	MAP1LC3B
PANX1	RPL10	NFE2L2
PLIN2	RPL15	BECN1
GABPB1	RPSA	ALOX15
GABPB1-AS1	FSCN1	VDAC2
ELAVL1	CBX3	VDAC3
CA9	EWSR1	ATG5
TTBK2	TRIM28	ATG7
ITGB8	KHDRBS1	NCOA4
DECR1	LRPPRC	SLC3A2
MIR761	HNRNPD	PCBP1
TNF	CNBP	MIR214
FBXW7	ACTC1	TF
ATG16L1	BANF1	PRKAA1
ZFP36	DUT	LINC00336
DNAJB6	PDIA4	ATF4
ITGA6	RPL26	LAMP2
PRKAA2	RPL13A	MDM2
FANCD2	RPS13	SAT1
ALOX12	RPS7	PARK7
CD44	RPL7	RELA
MAPK1	PTBP1	NF2
MYC	CCT3	YAP1
EGLN1	MAP4	SIRT3
MAP3K5	PFN2	BAP1
GOT1	RPLP2	CTSB
HELLS	RPS3A	CBS
FH	RPLP0	PRDX6
SOCS1	ITIH3	IDH1
OTUB1	HMGN1	PTEN
CARS1	DHX9	TNFAIP3
MIR9-1	PDIA6	SREBF1
MIR137	RPL30	HMGB1
NFS1	SNRPD2	WWTR1

RB1
G3BP1
LINC00472
EPAS1
HIF1A
G0S2
HILPDA
HSPB1
HSPA5
TIGAR
PLA2G6
PRC1
MT1G
TGFBR1
UCHL1
GSTP1
VIM
ANXA1
FASN
YWHAE
MYH9
YWHAG
ALDOA
TUBA1A
YWHAB
PGK1
HSPA9
GNB3
ENO2
RPL5
YWHAZ
DLST
LDHB
HADHA
HSP90B1
GPI
CFL1
ACVR1B
UBA1
XRCC6
PRDX2
MYH10
DPYSL2
CKB
SNRPA
CCT6A
CCT4
CCT8
KSR2
HNRNPL
HNRNPM
WTAP
YBX1
SSBP1
PLXNA4
SLC25A31
MYL6B
RPS4X
RPL34
SNRPD1
RUFY1
NOLC1
NACA
NAP1L1
HNRNPA3
ALYREF
USP44
SNRPC
RACK1
RPL38
NGB
SNRPF
ERH
FAM120A
ATP5F1A
TBC1D5
WBP11
MUC16
CNTNAP4
YTHDF3
TEX264
PCBP3
RPL36A
RUFY3
ATP5F1B
ACTBL2
SNU13
IGKC
AHCY
STYK1
SESN2
PVT1
VDAC1
IDH2
JUN
HNRNPA1
MDM4
ARNTL
CISD1
PROM2
MIR424
FTH1
FTL
CP
SLC11A2
GSS
CYBB
PRNP
SLC40A1
ACSL1
STEAP3
MAP1LC3A
GCLC
SLC39A8
SLC39A14
GCLM
ACSL5
PCBP2
ACSL3
ACSL6
SAT2
FTMT
LPCAT3
MAP1LC3C
MAP1LC3B2
LONP1
HDDC3
POR
AKR1C2
AKR1C3
PEBP1
CCL5

VAPB	KBTBD3	IREB2
SMC3	KIAA1614	KEAP1
PDIA3	SRSF8	NOX1
ALOX15B	RTL1	NQO1
RBM10	H2AC1	PGD
ANO6	ABRAXAS2	PHKG2
GUCY1A1	IGHG4	PTGS2
RNF113A	H2AC21	RPL8
MIR7-1	TUBA4B	SLC1A5
ABCC1	IGKV1-5	SQLE
ACACA	IGLV3-19	ZEB1
ACO1	SIAH2	FADS2
ACSF2	AURKA	FDFT1
ALOX5	CASP8	G6PD
ATP5MC3	CDKN2A	GLS2
CHAC1	RIPK1	HMGCR
CRYAB	MIR4715	HSBP1
CS	CFTR	MIF
DPP4	TGFB1	MUC1
EMC2		

Supplementary table 6. Validation of activating transcription factor 4 in GSE7621 and GSE20163

Gene name	GSM184354	GSM184355	GSM184356	GSM184357	GSM184358	GSM184359	GSM184360	GSM184361	GSM184362	GSM505996	GSM505997	GSM505998	GSM505999	GSM506000	GSM506001
ATF4	9.68	9.29	9.96	10.50	9.48	9.23	9.71	9.38	9.50	9.89	9.87	9.88	9.75	9.66	9.49

Gene name	GSM506003	GSM506007	GSM506008	GSM506002	GSM506004	GSM506005	GSM506006	GSM506009	GSM506010	GSM506011	GSM506012	GSM184363	GSM184364	GSM184365
ATF4	9.36	9.86	10.19	10.10	10.00	10.42	9.42	10.16	10.11	10.14	9.92	10.12	10.42	10.38

Gene name	GSM184366	GSM184367	GSM184368	GSM184369	GSM184370	GSM184371	GSM184372	GSM184373	GSM184374	GSM184375	GSM184376	GSM184377	GSM184378
ATF4	9.48	10.61	10.21	9.98	10.05	9.68	9.89	9.45	10.54	10.40	10.53	10.33	10.95

Supplementary table 7. Differentially expressed genes in RNA-sequence

Symbol	CK-1_fpkm	CK-2_fpkm	CK-3_fpkm	CK-4_fpkm	T1-1_fpkm	T1-2_fpkm	T1-3_fpkm	T1-4_fpkm	CK_mean	T_mean	log2(fc)	P-Value
Cers4	6.3	10.12	2.41	9.66	0.31	0.26	0.35	0.31	7.1225	0.3075	-4.533725401	3.87132129256651e-28
Dcafl5	5.15	4.05	6.35	5.38	0.99	1.81	1.57	1.36	5.2325	1.4325	-1.868965267	9.55067541478993e-18
Slc6a3	13.41	14.94	16.73	14.66	5.59	4.83	7.42	4.88	14.935	5.68	-1.394734403	1.47279691155974e-17
Afg1l	3.3	3.69	2.84	3.52	1.54	0.95	1.24	1.56	3.3375	1.3225	-1.335500114	9.02554031506896e-12
Gm21994	0.48	0.57	0.53	0.33	0	0	0	0	0.4775	0.001	-8.899356923	1.34224269307372e-10
Brd3	2.59	1.51	1.31	2.76	4.88	11.27	11.14	13.03	2.0425	10.08	2.30308765535423	1.91160298044628e-10
Acy1	6.36	2.23	7.59	6.66	0.73	1.51	0.72	1.42	5.71	1.095	-2.382559876	2.11299124841803e-08
Ints6l	3.92	3.6	2.93	2.6	1.64	1.38	1.93	1.42	3.2625	1.5925	-1.034684529	2.20931903178101e-08
Xpo4	4.23	7.84	2.81	0.16	0.3	0.17	0.26	0.11	3.76	0.21	-4.162271429	1.45445019253273e-07
Slc25a51	4.44	1.42	38.87	33.93	0.95	0.98	1.17	1.37	19.665	1.1175	-4.13728345	1.97561410481872e-07
Lars2	25.51	19.18	22.87	28.9	51.97	58.25	34.75	54.12	24.115	49.7725	1.04541804320044	3.63675000776172e-07
Hist1h3d	5.89	0.96	4.94	0.19	0.2	0.02	0.14	0.09	2.995	0.1125	-4.734559096	3.94466113826767e-07
Gh	0	0	0	0	0.12	1.03	6.74	28.09	0.001	8.995	13.1349075661388	7.85043146714084e-07
Kcnq5	5.52	4.89	14.97	16.28	2.68	2.61	1.89	3.17	10.415	2.5875	-2.009032072	1.38438155348999e-06
Dennd1a	20.63	3.14	17	2	1.35	1.03	1.34	1.2	10.6925	1.23	-3.119868987	1.62301439247113e-06
Zfx	1.79	1.39	0.82	2.43	0.56	0.63	0.58	0.54	1.6075	0.5775	-1.476925886	2.13904047960251e-06
Mef2d	34.47	2.36	2.56	33.2	1.29	1.19	2.08	1.05	18.1475	1.4025	-3.69369814	2.69206585905747e-06
Ttc39b	28.04	2.2	3.38	4.8	1.49	1.52	0.96	1.18	9.605	1.2875	-2.899213182	4.1993266556605e-05
Cct8l	0.04	0.1	0.1	0.05	0	0	0	0	0.07	0.0	-6.1799	4.66492

Symbol	CK-1_fpkm	CK-2_fpkm	CK-3_fpkm	CK-4_fpkm	T1-1_fpkm	T1-2_fpkm	T1-3_fpkm	T1-4_fpkm	CK_mean	T_mean	log2(fc)	P-Value
1									25	01	0909	553544694e-05
Zfp597	3.64	0.26	0.77	1.07	0.35	0.18	0.14	0.11	1.435	0.195	-2.879504708	4.89818533873098e-05
Nbl1	6.88	4.66	12.9	1.51	1.32	1.43	1.88	1.33	6.4875	1.49	-2.122350303	5.24249000352039e-05
Zcchc2	5.01	5.26	1.91	4.2	2.07	1.66	1.62	1.27	4.095	1.655	-1.307032235	5.4350786095032e-05
Tab2	1.24	10.07	19.43	1.04	0.82	0.97	1.08	0.68	7.945	0.8875	-3.162228195	5.65431786391545e-05
Capn11	0.13	0.15	0.1	0.14	0.28	0.72	0.52	0.25	0.13	0.4425	1.76716583194191	5.7695326101614e-05
Ctc1	6.9	1.46	6.28	6.6	1.97	1.45	1.64	1.54	5.31	1.65	-1.686245837	6.06335022610455e-05
Tmc7	3.46	3.17	2.05	3.19	1.53	0.33	0.44	0.22	2.9675	0.63	-2.235824296	6.12347550506505e-05
Scrib	1.58	7.86	1.83	8.89	0.82	0.98	1.48	1.02	5.04	1.075	-2.229087074	6.780243813951e-05
Grip2	5.51	1.92	0.8	0.46	0.15	0.57	0.09	0.19	2.1725	0.25	-3.119356177	7.1940796982841e-05
Otos	0	0	0	0	0.29	0.09	0.19	0.24	0.001	0.2025	7.66177809777199	7.88598740537312e-05
Cflar	2.74	9.86	4.27	4.62	0.71	1.59	2.45	1.3	5.3725	1.5125	-1.828658435	7.96485257261172e-05
Cfap300	1.5	0.74	1.24	0.87	3.17	4.58	5.11	1.28	1.0875	3.535	1.70069481407331	0.000115447404885874
Slc35f3	1.32	0.78	1.97	1.14	1.77	2.07	11.23	11.26	1.3025	6.5825	2.33735224376755	0.000193087791244394
Hdgfl2	2.54	4.59	4.82	6.4	1.97	1.93	2.74	1.21	4.5875	1.9625	-1.225015504	0.00020321333170489
Ankrd24	3.94	10.31	11.01	14.14	3.51	4.96	2.78	1.7	9.85	3.2375	-1.605243532	0.000233967524269775
Klhl23	1.56	7.77	15.58	15.21	2.41	2.73	2.59	2.17	10.03	2.475	-2.018821176	0.000277719669511545
Zdhhc7	2.38	2.34	4.06	4.19	4.05	7.91	8.92	7.28	3.2425	7.04	1.1184688543856	0.000308508838896285
Olfr690	0.3	0.23	0.29	0.29	0.15	0.11	0.1	0.04	0.2775	0.1	-1.472487771	0.000378361625263441
Rgs6	15.26	13.1	17.2	10.7	39	42.6	39.9	13.4	14.0	33.	1.2601	0.00039

Symbol	CK-1_fpkm	CK-2_fpkm	CK-3_fpkm	CK-4_fpkm	T1-1_fpkm	T1-2_fpkm	T1-3_fpkm	T1-4_fpkm	CK_mean	T_mean	log2(fc)	P-Value
		2	3	7		8	1	5	95	76	3142613676	2863893656829
Gm9887	0.49	0.03	0.2	0.22	0	0	0	0	0.235	0.001	-7.876516947	0.000414964836524719
Gm7072	0.08	0.05	0.12	0.57	0.16	1.72	1.68	3.24	0.205	1.7	3.05183893151962	0.000564149098862171
Slc16a7	3.08	0.57	0.54	3.62	0.45	0.45	0.19	0.57	1.9525	0.415	-2.234139307	0.000603354861965218
Zfyve1	7.77	6.5	15.12	9.67	2.86	5.75	3.61	5.73	9.765	4.4875	-1.121708105	0.000628817616803298
Usp31	0.23	0.04	0.91	0.83	14.77	0.87	1.06	16.65	0.5025	8.3375	4.05241935489302	0.000684958448442069
Rora	10.32	19.55	11.64	8.8	4.33	3.69	5.15	8.95	12.5775	5.53	-1.185493804	0.000712240333427123
Zscan25	0.62	0.77	0.8	0.69	1	0.57	6.92	6.7	0.72	3.7975	2.39898115305977	0.000928437081575364
Fetub	0.18	0.18	0.07	0.2	0.4	0.4	0.31	0.44	0.1575	0.3875	1.29884448177432	0.000935789217885346
Stpg2	0.04	0.08	0.05	0.04	0.17	0.13	0.16	0.13	0.0525	0.1475	1.49032562658308	0.000956003499157802
Dpp3	2.72	0.76	2.02	0.97	8.88	11.38	5.87	1.09	1.6175	6.805	2.07282944951149	0.00119779381485161
Nmu	0	0	0	0	0.15	0.07	0.08	0.1	0.001	0.1	6.64385618977473	0.001242398860928
Lcn2	0.11	0.27	0.33	0.18	0.63	0.34	0.94	0.79	0.2225	0.675	1.60108216608443	0.00139318608847289
Akap2	17.23	6.12	8.9	17.26	25.41	26.69	30.22	21.6	12.3775	25.98	1.06968148148572	0.00144370042153591
F2rl2	0.14	0.36	0.26	0.26	0.6	0.35	0.67	0.66	0.255	0.57	1.16046467219325	0.00153277388049671
Arntl	1.62	1.26	1.93	1.3	2.18	8.3	6.24	1.6	1.5275	4.58	1.58417521827821	0.00161232086450592
Klk1	0	0	0	0	0.12	0.14	0.05	0.07	0.001	0.095	6.56985560833095	0.00171494003652082
Exosc8	1.41	1.12	1.33	1.11	6.18	2.17	2.92	1.56	1.2425	3.2075	1.3682034135274	0.00177914875601234
Rgmb	18.57	4.13	25.1	9.49	4.27	6.31	4.93	5.29	14.3225	5.2	-1.461699809	0.00180112734844274
Kif28	0	0	0	0.01	0.1	0.02	0.03	0.03	0.00	0.0	4.1699	0.00184

Symbol	CK-1_fpkm	CK-2_fpkm	CK-3_fpkm	CK-4_fpkm	T1-1_fpkm	T1-2_fpkm	T1-3_fpkm	T1-4_fpkm	CK_mean	T_mean	log2(fc)	P-Value
									25	45	2500144231	141946874417
Phactr4	1.08	4.82	5.51	5.09	1.23	1.25	1.68	2.02	4.125	1.545	-1.416787281	0.00184146415734207
Eml1	13.33	8.53	1.42	1.66	1.47	1.34	1.75	1.75	6.235	1.5775	-1.982749555	0.00190489282625298
Phf12	0.3	0.71	0.21	0.43	2.3	0.65	0.95	1.02	0.4125	1.23	1.57619229109342	0.00201886763607049
Ccdc78	0.3	0.08	0.17	0.11	0.57	0.39	0.36	0.41	0.165	0.4325	1.39023410827827	0.00202279695964735
atf4	0.07	0.02	0.97	1	2.09	9.89	15.47	1.32	0.515	7.1925	3.80384897855607	0.00204801130951648
Card19	0.12	0.28	0.12	0.26	0.36	0.42	0.74	1.4	0.195	0.73	1.90442234001777	0.00218769444367036
Ipp	0.95	0.17	0.83	0.57	6.23	5.95	0.76	0.61	0.63	3.3875	2.42679721274132	0.00221229651722637
Chil3	0.11	0.12	0.06	0	0.26	0.14	1.05	0.47	0.0725	0.48	2.72698150559358	0.0025841470029897
Zfp719	7.94	1.79	1.48	12.02	1.7	1.62	1.73	1.6	5.8075	1.6625	-1.804562908	0.00268036516319946
Gm42791	0.34	0.31	0.2	0.1	0.08	0	0.03	0.03	0.2375	0.035	-2.762500686	0.00272044964611627
Furin	14.1	0.56	13.81	1.01	1.47	1.77	0.82	0.88	7.37	1.235	-2.577153578	0.00275786252933902
Evi2b	0.01	0	0	0.05	0.16	0.05	0.17	0.1	0.015	0.12	3	0.00281386002942408
Mroh1	0.82	0.72	1.1	0.53	0.82	1.49	2.48	2	0.7925	1.6975	1.09892873410532	0.00287021406905986
Acot3	0.87	0.29	0.85	0.13	0.1	0.13	0.21	0.18	0.535	0.155	-1.787270676	0.00287039183883331
Zfp644	2.51	2.76	3.18	1.73	5.32	2.33	4.04	37.74	2.545	12.3575	2.27964934506408	0.00289277484874555
4632433K11Rik	0.08	1.18	0.11	1.42	0.11	0.14	0.12	0.08	0.6975	0.1125	-2.632268215	0.00309126054619311
Mettl4	0.85	2.16	0.76	2.52	0.32	0.8	0.6	0.74	1.5725	0.615	-1.354401702	0.00318751060125348
Zswim1	9.99	2.04	4.63	12.3	1.07	0.57	1.36	4.3	7.24	1.825	-1.988093233	0.00335406208418309
Mcm	1.05	1.9	0.93	1.7	4.09	3.56	1.34	4.02	1.39	3.2	1.2212	0.00342

Symbol	CK-1_fpkm	CK-2_fpkm	CK-3_fpkm	CK-4_fpkm	T1-1_fpkm	T1-2_fpkm	T1-3_fpkm	T1-4_fpkm	CK_mean	T_mean	log2(fc)	P-Value
7									5	525	8393488861	118502536607
Traf3	3.73	2.8	2.38	11.52	1.61	1.48	2.73	1.66	5.1075	1.87	-1.449579029	0.00347723766569296
Nyap1	4.54	3.82	2.07	6.27	8.7	8.85	5.11	21.06	4.175	10.93	1.38844529831049	0.00348871559584424
Tut7	3.12	10.45	10.05	7.94	1.87	4.23	4.34	3.54	7.89	3.495	-1.174732845	0.00351201194038397
Arhgef19	1.32	1.8	10.63	2.65	1.21	1.01	1.41	1.13	4.1	1.19	-1.784662336	0.00361518728465784
Cd2bp2	8.27	6.27	12.96	8.85	24.61	11.98	11.43	27.16	9.0875	18.795	1.04839355123606	0.0036808607548267
Kcnh6	0.89	0.09	0.74	0.2	0.15	0.05	0	0.08	0.48	0.07	-2.777607579	0.00388302134831273
Gm18336	0.18	0.31	0.24	0.28	0.1	0.09	0.13	0.18	0.2525	0.125	-1.014355293	0.003927109713374
Mtmr4	8.65	2.91	2.85	20.54	26.25	20.27	36.67	31.01	8.7375	28.55	1.70819828998741	0.00397023557071666
Cyp2e1	0.27	0.66	0.15	0.17	0.12	0.1	0.06	0.07	0.3125	0.0875	-1.836501268	0.00398850210505474
Zfp819	0.14	0.24	0.2	0.14	0.31	0.42	0.52	0.27	0.18	0.38	1.07800251200127	0.00406138365016447
Prl	0	0	0	0	0	0.14	2.07	3.99	0.001	1.55	10.5980525001616	0.00407819075812857
Tktl2	0.09	0.15	0.13	0.12	0.04	0.06	0.08	0.06	0.1225	0.06	-1.029747343	0.00413582723050193
Abi2	9.19	54.83	12.11	12.57	45.05	70.24	84.9	76.29	22.175	69.12	1.64016911297504	0.0042685872851063
Sbno2	1.04	1.2	1.68	2.7	0.93	0.48	1.12	0.45	1.655	0.745	-1.151518886	0.00477381887761024
Numb	2.76	2.53	3.32	4.75	13.34	15.32	3.18	4.43	3.34	9.0675	1.44085673749623	0.0047938999994084
Nfyb	0.64	1.1	0.54	0.64	2.14	1.21	0.7	5.34	0.73	2.3475	1.68515678877376	0.00515306363899477
Katnal2	0.37	0.52	0.56	0.68	0.84	0.5	2.03	2.46	0.5325	1.4575	1.45264245297466	0.00515618856293695
Ccdc148	0.38	0.76	5.93	0.75	0.39	0.55	0.36	0.51	1.955	0.4525	-2.11117891	0.00523023570196876
Fam1	4.65	2.5	2.22	3.77	0.86	0.77	1.33	2.71	3.28	1.4	-1.2125	0.00533

Symbol	CK-1_fpkm	CK-2_fpkm	CK-3_fpkm	CK-4_fpkm	T1-1_fpkm	T1-2_fpkm	T1-3_fpkm	T1-4_fpkm	CK_mean	T_mean	log2(fc)	P-Value
14a2									5	175	44635	805745449636
Slc25a13	0.07	0.05	0.04	0.12	0.2	0.12	0.17	0.16	0.07	0.1625	1.21501289097085	0.00579636769035405
Btnl10	0	0	0	0	0.03	0.04	0.03	0.02	0.001	0.03	4.90689059560852	0.00581648718365705
Armcx2	12.21	45.96	47.36	5.09	13.84	6.03	6.25	7.09	27.655	8.3025	-1.735922619	0.00598252565703073
Cdan1	2.37	0.6	0.38	0.19	0.22	0.38	0.21	0.12	0.885	0.2325	-1.928446739	0.00608866520052356
Grid2ip	0.38	0.44	0.32	0.52	0.41	1.98	2.88	0.37	0.415	1.41	1.76451192105187	0.00612340273851092
Zkscan3	0.64	8.5	1.59	4.26	1.1	1.15	1.22	1.04	3.7475	1.1275	-1.732801045	0.00617659823361508
Pla2g2f	0.25	0.17	0.1	0.09	0.41	0.4	0.26	0.26	0.1525	0.3325	1.1245450979383	0.00636075401447906
Zfp46	13.34	20.05	6.58	12.35	4.01	4.13	10.92	4.49	13.08	5.8875	-1.151635481	0.00652082515036259
Fshr	0	0	0	0	0.03	0.02	0.03	0.03	0.001	0.0275	4.78135971352466	0.00674577453914929
Egr2	2.09	1.47	2.77	4.5	1.37	0.79	1.6	1.58	2.7075	1.335	-1.020121596	0.00681188746579875
Med14	2.56	2.75	2.5	2.85	3.13	2.13	11.42	11.53	2.665	7.0525	1.4039992278264	0.00695414439747604
Galr2	0	0	0	0	0	0.06	0.12	0.1	0.001	0.07	6.12928301694497	0.00712910916939857
Tbata	0.87	0.63	0.37	0.44	0.23	0.29	0.21	0.38	0.5775	0.2775	-1.057333175	0.00731865555850868
Gpr63	0.07	0.49	0.02	0.06	0	0.03	0.02	0.03	0.16	0.02	-3	0.00737224330545591
Rapgef4	88.26	95.03	56.39	47.38	49.75	24.72	18.4	48.65	71.765	35.38	-1.020346361	0.00744078073923353
Crcp	8.32	11.2	7.06	6.61	1.11	7.3	1.83	1.98	8.2975	3.055	-1.441504344	0.00770056195582861
Nmur1	0.07	0.09	0.04	0.12	0.16	0.19	0.31	0.24	0.08	0.225	1.49185309632967	0.00785740908194629
1700061G19Rik	0.06	0.03	0.02	0.08	0.01	0	0	0.02	0.0475	0.0075	-2.662965013	0.00788218548668691
Il13ra	0.2	0.28	0.25	0.32	0.21	0.1	0.09	0.08	0.26	0.1	-1.1292	0.00855

Symbol	CK-1_fpkm	CK-2_fpkm	CK-3_fpkm	CK-4_fpkm	T1-1_fpkm	T1-2_fpkm	T1-3_fpkm	T1-4_fpkm	CK_mean	T_mean	log2(fc)	P-Value
2									25	2	83017	645557542583
Prrt1	8.26	1.05	9.06	10.91	3.02	2.04	2.83	2.79	7.32	2.67	-1.455003907	0.00861707585076484
Tro	5.59	4.18	2.73	4.81	6.91	10.22	23.31	3.67	4.3275	11.0275	1.34950003572558	0.00880717742425305
Scaper	5.61	16.57	16.12	6.68	3.15	4.89	7.57	5.59	11.245	5.3	-1.085219396	0.00882063462407781
Katna1	1.36	1	1.32	1.11	6.28	0.72	2.43	3.61	1.1975	3.26	1.4448463084949	0.00893388736914709
Olfr316	0.35	0.19	0.33	0.36	0.04	0.19	0.15	0.16	0.3075	0.135	-1.187627003	0.00899826333368229
Tmem9b	11.93	14.41	8.96	9.9	9.48	50.82	11.66	51.91	11.3	30.9675	1.45443214659478	0.0090568721076865
Slc2a13	10.68	8.28	14.54	11.12	12.05	32.56	12.63	32.17	11.155	22.3525	1.00274568408596	0.0091409004682078
Hsd17b2	0.32	0.44	0.26	0.07	0.67	0.77	0.65	0.48	0.2725	0.6425	1.23744022441695	0.00936063322280197
Scamp1	90.41	108.9	34.18	42.18	37.49	46.21	20.59	25.1	68.9175	32.3475	-1.091216152	0.00946341282632391
8030474K03Rik	0.02	0.09	0.03	0.01	0	0	0	0	0.0375	0.001	-5.22881869	0.00964152617941515
Pdxp	6.13	53.8	8.38	61.35	11.46	12.38	7.92	7.78	32.415	9.885	-1.713348702	0.010109095942946
Rpl37	19.01	0.52	4.66	1.39	39.97	17.96	32.83	75.97	6.395	41.6825	2.70442554481335	0.0104097817246535
Mre11a	6.7	1.89	3.97	5.92	1.3	0.55	0.77	3.69	4.62	1.5775	-1.550252846	0.010724334806051
Ptp4a1	5.81	0.47	0.35	0.84	0.33	0.43	0.36	0.69	1.8675	0.4525	-2.045118546	0.0108041010955205
Cacna1f	0.01	0	0	0	0.01	0.03	0.03	0.06	0.0025	0.0325	3.70043971814109	0.0109987417069304
Tdo2	0.12	0.46	0.22	0.38	0.06	0.18	0.08	0.13	0.295	0.1125	-1.390789953	0.0110005521097666
Arid5b	3.81	9.2	5.15	3.96	0.77	3.06	3.19	2.9	5.53	2.48	-1.15693936	0.0111021139586656
Urb2	0.21	0.18	0.39	0.8	1.76	1.73	0.65	0.42	0.395	1.14	1.52910926598764	0.0113561757506163
Ect2l	0.03	0.07	0.12	0.08	0.04	0	0	0	0.07	0.0	-2.9068	0.01151

Symbol	CK-1_fpkm	CK-2_fpkm	CK-3_fpkm	CK-4_fpkm	T1-1_fpkm	T1-2_fpkm	T1-3_fpkm	T1-4_fpkm	CK_mean	T_mean	log2(fc)	P-Value
									5	1	90596	9139227909
Ppp1r26	0.3	0.34	1.22	0.36	0.24	4.78	0.74	3.45	0.555	2.3025	2.05264147974123	0.0115739467076972
Upk1a	0.27	0.12	0.16	0.26	0.09	0.1	0.06	0.09	0.2025	0.085	-1.252387162	0.0116408041365538
Ptch1	0.92	3.16	1.19	4.12	4.97	5	4.69	6.81	2.3475	5.3675	1.193125128	0.0116795768268454
Rock1	1.2	2.6	3.69	4.47	1.11	1.06	2.24	1.45	2.99	1.465	-1.02924482	0.0117151176892955
Nefh	2.71	1.13	2.3	0.44	1.33	17.18	8.27	1.21	1.645	6.9975	2.08875199783767	0.0118650128929278
Nrg3	2.61	1.28	2.76	5.91	2.24	0.72	1.22	1.2	3.14	1.345	-1.223158386	0.012280964127 25
Kctd19	0.04	0	0.05	0.1	0	0	0	0	0.0475	0.001	-5.569855608	0.0125055361558365
Lipo2	0.22	0.17	0.08	0.2	0.05	0.03	0.09	0.11	0.1675	0.07	-1.258734268	0.0125332248931304
Bpifb3	0.02	0	0	0	0	0.09	0.14	0.24	0.005	0.1175	4.55458885167764	0.0127357893116413
Usp36	0.57	3.65	4.73	6.32	0.52	1.06	0.62	2.53	3.8175	1.1825	-1.690787973	0.0130381681094837
Gpr4	0.27	1.71	2	2.08	0.52	0.98	0.25	0.27	1.515	0.505	-1.584962501	0.0130868338939183
Mkl2	54.11	21.24	55.88	59.86	26.44	28.84	10.33	29.64	47.7725	23.8125	-1.004461284	0.0132407782335281
Ugt2a2	0	0	0	0	0.14	0.12	0	0.02	0.001	0.07	6.12928301694497	0.0133045196428166
Crocc	0.84	3.69	1.26	1.38	0.43	0.73	0.99	1.02	1.7925	0.7925	-1.177490279	0.0137668336658464
Padi4	0	0	0.03	0	0.03	0.08	0.03	0.19	0.0075	0.0825	3.4594316186373	0.0137735443266406
Nhs	0.48	0.34	1.17	0.51	3.67	3.09	0.51	0.6	0.625	1.9675	1.6544355408454	0.0138474394314161
B430218F22Rik	0.15	0.07	0.08	0.03	0.22	0.18	0.26	0.17	0.0825	0.2075	1.33064531198847	0.0138608427607774
Tmsb15b2	0.06	0.24	0.14	0.07	0.35	0.27	1.2	0.43	0.1275	0.5625	2.14135584924554	0.0139105572446202
Elp5	51.33	42.7	43.2	54.7	46.9	12.7	7.93	15.1	48.0	20.	-1.2147	0.01455

Symbol	CK-1_fpkm	CK-2_fpkm	CK-3_fpkm	CK-4_fpkm	T1-1_fpkm	T1-2_fpkm	T1-3_fpkm	T1-4_fpkm	CK_mean	T_mean	log2(fc)	P-Value
		3	8	7	5	5		4	275	6925	52756	14714716098
Gm44973	0	0	0.42	0.88	0	0	0	0	0.325	0.001	-8.344295908	0.0146720478978469
Smco3	1.57	2.61	1.66	6.16	6.94	6.54	5.79	5.81	3	6.27	1.06350294230616	0.0149132027624635
Col10a1	0.04	0.09	0.07	0.09	0.03	0.01	0.05	0.02	0.0725	0.0275	-1.398549376	0.0150649916496632
Rps6ka3	6.7	1.02	8.5	0.97	1.37	1.28	1.69	1.38	4.2975	1.43	-1.587482493	0.0152914771689083
Tssk3	0	0.03	0	0	0.06	0.02	0.1	0.18	0.0075	0.09	3.58496250072116	0.0154365579932637
Casd1	9.64	16.31	57.53	8.4	9.8	9.34	8.89	7.91	22.97	8.985	-1.354160448	0.0158160661610132
Hoxd4	0	0	0	0	0.06	0.07	0	0.02	0.001	0.0375	5.22881869049588	0.0159257165856652
Trim34b	0.02	0.05	0	0	0.05	0.08	0.17	0.09	0.0175	0.0975	2.47804729680464	0.015953683519435
Pgap1	4.63	13.81	6.61	4.53	2.36	2.58	4.56	5.22	7.395	3.68	-1.006844381	0.0169714521332999
Gm12666	0	0	0	0	0.37	0.04	0	0.22	0.001	0.1575	7.29920801838728	0.0173327181847211
Ltf	0.88	0.49	1.05	0.14	1.66	0.97	2.4	1.35	0.64	1.595	1.31741261376487	0.017442877258715
Ap5b1	1.98	0.94	0.18	0.17	0.36	0.15	0.23	0.23	0.8175	0.2425	-1.753233983	0.017569908346354
Lypd6	7.42	8.48	8.63	7.66	5.36	1.21	0.46	4.49	8.0475	2.88	-1.48247186	0.017600924817956
Iqch	0.03	0.02	0.05	0.04	0.08	0.08	0.05	0.13	0.035	0.085	1.28010791919274	0.0176864860064981
AB124611	0.18	0.17	0.18	0.15	0.07	0.05	0	0.12	0.17	0.06	-1.502500341	0.0177226876359989
Trim15	0.13	0.19	0.03	0.15	0.02	0.05	0.05	0	0.125	0.03	-2.058893689	0.0178080708128209
Als2cr12	0.05	0	0	0	0.17	0.16	0.04	0.06	0.0125	0.1075	3.10433665981474	0.0187482295899556
Arxes2	35	42.28	35.68	34.36	33.57	5.52	6.58	17.28	36.83	15.7375	-1.226675019	0.0188576044190913
Spata	0.12	0.35	0.11	0.21	0.34	0.65	0.47	0.42	0.19	0.4	1.2508	0.01898

Symbol	CK-1_fpkm	CK-2_fpkm	CK-3_fpkm	CK-4_fpkm	T1-1_fpkm	T1-2_fpkm	T1-3_fpkm	T1-4_fpkm	CK_mean	T_mean	log2(fc)	P-Value
19									75	7	0810350053	45013837453
Pigr	0	0	0	0	0.09	0.12	0	0.02	0.001	0.0575	5.84549005094438	0.0190569279725574
Bmp15	0	0.03	0.02	0.05	0.09	0.06	0.05	0.08	0.025	0.07	1.48542682717024	0.0199841309012403
Bclaf3	3.46	2.99	2.9	2.93	1.11	0.65	0.01	1.59	3.07	0.84	-1.869777423	0.0204007959482506
Ptprm	2.73	9.41	2.61	2.42	2.39	1.94	2.04	1.97	4.2925	2.085	-1.041770751	0.0204176219593543
Pirb	0.03	0.1	0.1	0.05	0.14	0.16	0.17	0.11	0.07	0.145	1.05062607306997	0.0208804670875861
Ackr2	0.64	0.14	0.84	0.14	0.04	0.09	0.25	0.14	0.44	0.13	-1.7589919	0.0210105799084392
Kif21b	3.73	3.96	2.32	7.07	12.43	3.81	6.19	13.74	4.27	9.0425	1.08248562303528	0.0215249333646619
S100a9	0.3	0.32	1.11	0.14	0.67	0.86	1.91	1.98	0.4675	1.355	1.53525458146623	0.0218813791407411
Clec12b	0.04	0	0	0	0.1	0.04	0.05	0.13	0.01	0.08	3	0.0223072775612194
Samd10	2.09	0.77	1.76	0.39	4.51	0.92	3.48	4	1.2525	3.2275	1.36560649211198	0.0225332328622479
Zfp329	2.71	7.91	2.87	1.5	0.96	1.98	0.83	2.58	3.7475	1.5875	-1.239171886	0.0228394379979249
Ppip5k2	2.15	2.92	2.5	2.21	2.64	6.98	9.29	1.89	2.445	5.2	1.08867715807622	0.0231460238854324
Bbx	2.07	2.38	2.48	2.47	2.31	2.14	14.15	4.42	2.35	5.755	1.29215517156723	0.0234105831703283
Sycp3	0.38	1.04	0.32	0.2	0.16	0.27	0.21	0.15	0.485	0.1975	-1.296132094	0.0235540819710019
Tubb5	226.93	218.21	231.56	237.47	240.21	79.9	41.56	61.39	228.5425	105.765	-1.111600189	0.0236050641360062
Nipa1	1.96	1.2	0.98	0.85	1.33	1.92	0.59	14.79	1.2475	4.6575	1.90051595360438	0.0243555343754173
Gm49369	3.15	1.66	2.19	1.01	2.72	1.58	9.71	5.64	2.0025	4.9125	1.29465516473726	0.0250541817169368
Vmn2r56	0.04	0.02	0.04	0.02	0.02	0.02	0.01	0.01	0.03	0.015	-1	0.0253457504965785
Vwa5	0.84	0.94	3.92	1.25	2.99	3.46	4.89	3.59	1.73	3.7	1.1031	0.02541

Symbol	CK-1_fpkm	CK-2_fpkm	CK-3_fpkm	CK-4_fpkm	T1-1_fpkm	T1-2_fpkm	T1-3_fpkm	T1-4_fpkm	CK_mean	T_mean	log2(fc)	P-Value
b2									75	325	2928252367	20371694923
Shbg	0.15	0.07	0.16	0.15	0.07	0.02	0.08	0.03	0.1325	0.05	-1.40599236	0.0255247057171723
Osbpl6	1.29	1.24	5.82	1.24	0.94	0.89	1.29	1.17	2.3975	1.0725	-1.160553168	0.0257419338184744
Azgp1	0.06	0.06	0.1	0	0	0	0	0	0.055	0.001	-5.781359714	0.0259843312987323
Gm29609	0.53	0.34	1.33	0.3	0.18	0.46	0.27	0.13	0.625	0.26	-1.265344567	0.026929381466092
Zfp980	0.01	0.03	0.04	0.01	0	0	0.01	0	0.0225	0.0025	-3.169925001	0.0276064610529343
Rbp7	0	0	0	0	0	0.1	0.32	0.14	0.001	0.14	7.12928301694497	0.0277657701242151
C130050O18Rik	0.07	0.03	0.03	0.07	0.1	0.24	0.11	0.09	0.05	0.135	1.43295940727611	0.0279487113534884
Qtrt2	0	0.71	0.77	0	0	0	0	0	0.37	0.001	-8.531381461	0.0283395701039345
Lrg1	0.1	0.16	0.21	0.46	0.75	0.59	0.34	0.39	0.2325	0.5175	1.15432814639129	0.0284176673412296
Cngb3	0	0.02	0	0	0.03	0.03	0.02	0.06	0.005	0.035	2.8073549220576	0.0285038087380702
Sp2	0.3	1.55	2.12	1.25	0.21	0.74	0.69	0.57	1.305	0.5525	-1.240003437	0.0289867032435289
Gas2l1	2.53	1.61	3.14	6.48	0.75	1.83	1.72	2.44	3.44	1.685	-1.029659974	0.0290027888879267
Elane	0	0	0	0.05	0.08	0.06	0.18	0.04	0.0125	0.09	2.84799690655495	0.0294048308746105
Zdhhc20	15.72	4.85	15.3	2.84	3.66	3.3	2.96	7.13	9.6775	4.2625	-1.182934568	0.0300194208522629
Rab19	0.13	0.11	0.06	0.1	0.03	0.07	0	0.03	0.1	0.0325	-1.621488377	0.0300794262931189
Lalba	0.1	0.22	0.06	0	0	0	0	0	0.095	0.001	-6.569855608	0.0304414606482292
A830005F24Rik	0.66	1.14	0.32	0.35	0.29	0.35	0.2	0.3	0.6175	0.285	-1.115477217	0.0306357168265401
Areg	0.05	0.2	0.14	0.15	0.07	0	0	0.05	0.135	0.03	-2.169925001	0.031072871094793
Serpi	0.1	0.02	0.1	0.08	0.34	0.09	0.12	0.2	0.07	0.1	1.3219	0.03130

Symbol	CK-1_fpkm	CK-2_fpkm	CK-3_fpkm	CK-4_fpkm	T1-1_fpkm	T1-2_fpkm	T1-3_fpkm	T1-4_fpkm	CK_mean	T_mean	log2(fc)	P-Value
nf2									5	875	2809488736	38401295385
Amdhd1	0	0.1	0.02	0.02	0	0	0	0	0.035	0.001	-5.129283017	0.0316740245734693
Slc25a15	4.26	0.02	4.23	0.37	0.3	0.15	0.68	0.26	2.22	0.3475	-2.675474794	0.0325638764556614
Csnk1e	8.42	8.41	26.84	46.48	14.87	8.05	9.62	7.76	22.5375	10.075	-1.161547653	0.0329583751026475
Numa1	2.72	24.69	11.85	2.51	2.88	2.14	2.96	7	10.4425	3.745	-1.47942952	0.0345673722389346
Gm7347	0.04	0.05	0.02	0.02	0	0	0	0.02	0.0325	0.005	-2.700439718	0.0345725970895417
Asic1	28.86	3.53	27.94	29.11	5.23	13.47	4.82	13.44	22.36	9.24	-1.274955431	0.0346465941462231
Chd2	32.16	14.32	27.54	22.34	2.44	2	19.33	13.32	24.09	9.2725	-1.377404126	0.0355945775427534
Scn2a	9.46	9.13	14.09	71.18	57.11	78.55	76.27	50.37	25.965	65.575	1.33657767706455	0.0362453453366019
Rab25	0.06	0.08	0.05	0	0.1	0.15	0.24	0.11	0.0475	0.15	1.65896308216493	0.0368485197397315
Tnni3k	0.05	0.15	0.05	0.04	0.03	0.01	0.04	0.01	0.0725	0.0225	-1.688055994	0.0374059949170087
Sgsm1	4.08	6.66	20.86	5.03	2.85	6.73	3	4.49	9.1575	4.2675	-1.101562643	0.0377924333703723
Nat8f5	0.02	0.13	0.03	0.02	0.37	0.02	0.18	0.37	0.05	0.235	2.23266075679027	0.038305852364828
Gm6525	0.04	0.04	0.05	0.02	0	0	0	0	0.0375	0.001	-5.22881869	0.0383068944727465
Dusp9	0.15	0.18	0.31	0.27	0.19	0.05	0.08	0.13	0.2275	0.1125	-1.015941544	0.0389759112413499
Rimkla	9.7	9.59	1.09	7.53	2.67	3.99	1.94	3.97	6.9775	3.1425	-1.150797475	0.0394267419146958
Cd6	0.13	0.16	0.14	0.14	0	0.07	0.1	0.07	0.1425	0.06	-1.247927513	0.039828621379032
Gm826	0.24	0	0.21	0.13	0	0.04	0	0	0.145	0.01	-3.857980995	0.0400517234442893
Sult5a1	0.14	0.05	0.03	0.03	0.17	0.11	0.11	0.24	0.0625	0.1575	1.33342373372519	0.0402201672740124
Foxd	0	0	0	0	0.05	0.02	0	0.05	0.00	0.0	4.9068	0.04117

Symbol	CK-1_fpkm	CK-2_fpkm	CK-3_fpkm	CK-4_fpkm	T1-1_fpkm	T1-2_fpkm	T1-3_fpkm	T1-4_fpkm	CK_mean	T_mean	log2(fc)	P-Value
4									1	3	9059560852	34043357853
Upk3bl	0	0	0	0.09	0.08	0.1	0.1	0.2	0.0225	0.12	2.41503749927884	0.0419195068758315
Tmem189	0.32	3.05	0.69	0.29	14.71	1.01	3.1	1.79	1.0875	5.1525	2.24425719880317	0.0420266884707436
Gm8281	0.1	0.03	0.02	0.04	0	1.33	0.17	0.02	0.0475	0.38	3	0.0425702344353338
Zbtb34	1.72	1.12	0.52	1.58	3.35	0.66	2.78	3.68	1.235	2.6175	1.08367849534613	0.0439575077010362
Itprid2	7.54	11.38	10.07	6.1	8.8	0.93	1.41	3.7	8.7725	3.71	-1.241568856	0.0444495442275821
Wnt8b	0.03	0.08	0.08	0.1	0.19	0.21	0.12	0.07	0.0725	0.1475	1.02466205423427	0.0449907796038663
Dscam	4.85	3.2	2.55	4.01	12.15	3.64	1.67	17.1	3.6525	8.64	1.24214703935997	0.0456057716767243
Slfn4	0	0	0	0.02	0.02	0.01	0.08	0.03	0.005	0.035	2.8073549220576	0.0457202167754432
Lilrb4a	0.04	0	0	0.07	0.02	0.06	0.17	0.39	0.0275	0.16	2.5405683813627	0.0458828793904077
Ubl4b	0.06	0.12	0.11	0.07	0	0.04	0.05	0	0.09	0.0225	-2	0.0463024386587071
Pfas	8.08	6.56	3	1.17	0.51	1.88	1.32	3.84	4.7025	1.8875	-1.316951299	0.0464422974658693
Ppm1n	0	0	0	0.04	0.04	0.07	0.08	0.04	0.01	0.0575	2.52356195605701	0.0469445384541455
Guca1a	0.09	0	0.09	0	0.23	0.32	0.1	0.07	0.045	0.18	2	0.0484572589830815
Scaf8	7.32	5.99	6.71	9.7	0.7	7.08	3.73	2.81	7.43	3.58	-1.053402623	0.0494577654037799

Supplementary table 8. The top 100 ASV based on 16S rRNA gene sequencing

ASV_ID	MPTP1	MPTP2	MPTP3	MPTP4	MPTP5	Saline1	Saline2	Saline3	Saline4	Saline5
ASV1	3142	0	583	6390	5142	3649	3181	2048	6485	3473
ASV2	2568	6778	1393	2576	4847	5728	426	2140	1677	1434
ASV3	329	2199	1906	1252	2143	3950	1387	3905	2531	3479
ASV4	512	1371	2505	509	1069	401	2745	2767	1925	1294
ASV5	1550	1480	2300	737	823	4954	1103	1601	3912	1353
ASV6	17	0	165	0	0	2099	215	0	0	0
ASV7	679	0	1981	239	140	2677	5757	64	0	1448
ASV8	1457	5203	321	1915	3280	2677	584	480	930	790
ASV9	385	1063	964	1101	1614	1872	1106	1277	1195	1295
ASV10	785	878	551	679	1034	2990	852	634	531	958
ASV11	380	471	279	303	449	3131	1512	1243	701	480
ASV12	5561	94	85	222	166	377	0	94	13	0
ASV13	707	246	400	42	91	11	59	278	103	777
ASV14	88	76	63	38	98	1247	1809	389	100	599
ASV15	435	130	1790	66	133	101	84	462	367	4546
ASV16	0	0	750	0	0	1559	6114	0	0	16
ASV17	49	0	879	1061	891	1512	839	124	57	16
ASV18	405	470	259	304	555	1606	415	325	278	433
ASV19	16	0	307	243	440	3266	155	773	133	26
ASV	79	815	456	357	445	408	566	898	1104	718

20										
ASV 21	30	0	188	0	0	446	0	21	80	46
ASV 22	366	261	160	202	456	1970	378	286	384	278
ASV 23	0	497	437	249	434	866	313	636	571	787
ASV 24	59	252	505	79	226	74	645	533	300	258
ASV 25	110	637	336	484	728	968	268	1140	434	212
ASV 26	146	353	203	223	383	1115	347	191	233	362
ASV 27	1219	151	116	122	154	479	15	514	85	1360
ASV 28	0	0	227	512	329	629	697	1643	1057	72
ASV 29	199	199	272	123	130	1273	258	200	507	298
ASV 30	1428	12	18	139	105	126	0	8	34	28
ASV 31	838	157	82	31	70	159	523	246	301	107
ASV 32	191	235	129	153	303	916	321	248	155	302
ASV 33	124	136	346	100	105	1111	202	197	665	297
ASV 34	382	602	112	266	326	925	461	566	400	454
ASV 35	0	1856	0	0	0	0	0	45	1396	0
ASV 36	1864	0	0	0	0	179	0	0	12	0
ASV 37	631	0	18	5	3	284	142	52	10	12
ASV 38	486	543	68	246	329	944	458	496	330	398
ASV 39	143	349	260	313	417	633	128	290	367	147
ASV 40	754	287	246	0	25	720	1868	43	90	36
ASV 41	16	36	0	0	0	104	537	313	98	134
ASV	25	0	0	0	0	506	76	0	19	0

42										
ASV 43	4954	0	0	0	0	0	0	0	0	0
ASV 44	58	143	88	65	163	872	310	379	220	122
ASV 45	0	0	167	0	0	434	1516	0	0	0
ASV 46	119	90	97	89	110	543	321	296	314	231
ASV 47	136	166	91	179	235	602	301	484	363	188
ASV 48	94	298	189	212	314	465	149	563	282	141
ASV 49	3612	0	0	10	11	0	0	0	0	0
ASV 50	315	352	90	146	211	490	168	253	186	172
ASV 51	394	17	0	742	699	0	0	0	0	49
ASV 52	455	245	652	232	268	372	0	0	0	59
ASV 53	1468	0	9	0	0	895	317	615	109	67
ASV 54	417	217	29	50	45	1508	85	75	16	20
ASV 55	2382	0	0	29	0	0	0	0	0	0
ASV 56	582	176	274	77	92	41	341	190	282	224
ASV 57	115	76	175	115	126	168	206	387	232	93
ASV 58	49	0	45	523	712	399	331	228	139	105
ASV 59	444	11	14	0	0	20	118	0	0	70
ASV 60	86	153	161	85	51	325	218	393	188	112
ASV 61	0	0	0	560	760	378	308	211	152	102
ASV 62	150	168	0	71	165	778	65	101	115	68
ASV 63	0	270	120	189	295	310	106	470	229	93
ASV	29	224	34	319	649	0	202	0	107	9

64										
ASV 65	2699	351	0	0	0	0	0	0	0	0
ASV 66	660	336	100	91	226	352	0	38	87	37
ASV 67	1293	32	53	22	0	110	21	23	0	0
ASV 68	210	98	86	98	136	253	189	226	318	91
ASV 69	72	0	169	0	59	0	221	277	180	148
ASV 70	5	135	102	47	163	369	39	184	120	63
ASV 71	520	301	175	70	49	74	68	82	183	100
ASV 72	134	192	128	116	190	435	95	171	136	90
ASV 73	0	90	67	101	138	590	165	234	232	85
ASV 74	241	198	91	35	43	258	151	351	190	59
ASV 75	0	0	0	0	0	225	431	87	0	134
ASV 76	133	127	0	53	146	675	61	53	97	48
ASV 77	210	200	107	56	99	190	68	127	243	169
ASV 78	2433	0	0	0	0	27	0	0	0	0
ASV 79	814	30	278	14	12	143	114	51	48	146
ASV 80	0	48	0	99	101	31	123	0	0	24
ASV 81	458	157	45	40	45	174	0	318	127	41
ASV 82	294	1208	27	111	89	113	143	135	0	20
ASV 83	8	0	0	40	25	421	119	320	0	72
ASV 84	256	199	87	67	75	163	169	130	73	77
ASV 85	35	165	63	49	105	394	239	213	206	143
ASV	29	54	42	171	166	46	60	83	193	567

86										
ASV 87	147	169	80	122	117	318	40	273	196	134
ASV 88	0	31	55	54	77	466	132	196	109	112
ASV 89	92	162	79	66	155	42	172	0	208	156
ASV 90	35	193	72	146	229	310	118	225	125	70
ASV 91	37	164	173	0	0	133	57	108	127	16
ASV 92	0	46	62	0	0	107	539	6	0	4
ASV 93	0	0	0	0	0	216	52	33	122	133
ASV 94	140	104	103	66	100	290	92	153	96	60
ASV 95	1269	0	0	0	0	0	0	0	9	0
ASV 96	183	116	101	132	216	321	39	18	83	71
ASV 97	0	0	514	221	407	21	0	576	42	0
ASV 98	54	154	99	88	166	176	121	131	117	149
ASV 99	141	235	68	91	134	239	104	151	135	112
ASV 100	891	17	0	0	17	75	14	15	0	0

663
