# Supplementary material for: Gut microbiota-derived TNF-α triggers dopaminergic neuron ferroptosis via TNFR1-NF-κB-ATF4 axis in Parkinson’s disease
Source: Cell Death Discov. 2026 Aug 3;12:433. doi: 10.1038/s41420-026-03281-x (PMC13720208; doi:10.1038/s41420-026-03281-x)
Supplement: Supplementary file 2 [file 41420_2026_3281_MOESM2_ESM.docx]

**Western blot**

**Figure 6H**

**Original bands: ATF4 45KDa**

**Original bands: β-Actin 42KDa**

**Figure 7D**

**Original bands: ATF4 45KDa**

**Original bands: ACSL4 79KDa**

**Original bands: β-Actin 42KDa**

**Original bands: α-Syn 14-28-42KDa**

**Original bands: GPX4 20KDa**

**Original bands: SLC7A11 55KDa**

**Figure 7O**

**Original bands: ACSL4 79KDa**

**Original bands: β-Actin 42KDa**

**Original bands: ATF4 45KDa**

**Original bands: GPX4 20KDa**

**Original bands: SLC7A11 55KDa**

**Figure 8E**

**Original bands: β-Actin 42KDa**

**Original bands: ATF4 45KDa**

**Original bands: TNFR1 55KDa**

**Figure 8G**

**Original bands: β-Actin 42KDa**

**Original bands: TNFR2 70KDa**

**Original bands: ATF4 45KDa**

**Figure 8L**

**Original bands: β-Actin 42KDa**

**Original bands: ACSL4 79KDa**

**Original bands: IκBα 39KDa**

**Original bands: P-IκBα 40KDa**

**Original bands: P-P65 65KDa**

**Original bands: GPX4 20KDa**

**Original bands: ATF4 45KDa**

**Original bands: SLC7A11 55KDa**

**Original bands: P65 65KDa**

**Figure S2C**

**Original bands: β-Actin 42KDa**

**Original bands: TNFR1 55KDa**

**Figure S2D**

**Original bands: TNFR2 70KDa**

**Original bands: β-Actin 42KDa**

**Figure S3A**

**Original bands: β-Actin 42KDa**

**Original bands: SLC7A11 55KDa**

**Original bands: GPX4 20KDa**

**Original bands: ATF4 45KDa**

**Original bands: α-Syn 14-28KDa**

**Original bands: TH 60KDa**

**Original bands: ACSL4 79KDa**
